# Correction: Potential public health impacts of gonorrhea vaccination programmes under declining incidences: A modeling study

**DOI:** 10.1371/journal.pmed.1004573

**Published:** 2025-04-01

**Authors:** Lin Geng, Lilith K. Whittles, Borame L. Dickens, Martin T. W. Chio, Yihao Chen, Rayner Kay Jin Tan, Azra Ghani, Jue Tao Lim

The Funding statement for this paper is incorrect. The correct Funding statement is as follows: LKW acknowledges funding from the Wellcome Trust (grant number 218,669/Z/19/Z). AG and LKW acknowledge funding from the Medical Research Council (MRC) Centre for Global Infectious Disease Analysis (MR/X020258/1), funded by the UK MRC and carried out within the framework of the Global Health EDCTP3 Joint Undertaking, supported by the EU. This work is also supported by Nanyang Technological University, Singapore—Imperial Research Collaboration Fund (INCF-2023–007 to JTL and AG). LJT is an Assistant Professor at Nanyang Technological University. AG is a Professor, and LKW is a Lecturer at Imperial College London. The funders have no role in study design, data collection and analysis, decision to publish, or preparation of the manuscript.
