# Supplementary material for: Potential public health impacts of gonorrhea vaccination programmes under declining incidences: A modeling study
Source: PLoS Med. 2025 Feb 7;22(2):e1004521. doi: 10.1371/journal.pmed.1004521 (PMC11805383; doi:10.1371/journal.pmed.1004521)
Supplement: S1 Text — (DOCX) [file pmed.1004521.s001.docx]

**Supplementary Information**

**1. Model details**

**1.1 Model of gonorrhea transmission**

The force of infection, i.e. the rate of acquisition of infection for an uninfected individual, in sexual activity group $j$, $\lambda_{j} \left( t \right)$, depends on the rate of partner change per year in each sexual activity group ($c_{j}$); the level of assortativity in sexual mixing between groups ($\epsilon$, where $\epsilon$ denotes proportionate mixing and $\epsilon=1$ denotes fully assortative contact [1]; the prevalence of infectees in each group ($\frac{C_{i}\left( t \right)}{N_{i}\left( t \right)}$); and the rate of transmission, which we allow to change linearly over time from the $\beta$ at $t_{0}$ = 2004, to last calibrated data year β at $t_{1}$ = 2018. A linear trend was used as it could characterise the historical changes in gonorrhea incidence well (See Fig A3 below), in comparison to an exponentially decaying functional form. The force of infection is calculated as follows:

$$\begin{aligned} \lambda_{j}\left( t \right)=c_{j}\left( \beta_{t_{0}}+t\frac{\left( \beta_{t_{1}}-\beta_{t_{0}} \right)}{\left( t_{1}-t_{0} \right)} \right)\left( \epsilon\frac{C_{j}\left( t \right)}{N_{j}\left( t \right)}+\left( 1-\epsilon\right)\left( \sum_{i\in\left\{ L,H \right\}} \pi_{i}\left( t \right)\frac{C_{i}\left( t \right)}{N_{i}\left( t \right)} \right) \right)\#\left( 1 \right) \end{aligned}$$

Where $\pi_{i}\left( t \right)=\frac{c_{j}N_{j}\left( t \right)}{\sum_{i\in\left\{ L,H \right\}} c_{i}N_{i}\left( t \right)}$ is the proportion of all partnerships in the population that involve a member of group $j$.

The rate of screening (i.e. testing in the absence of symptoms, which applies to asymptomatic and uninfected individuals), $\eta_{j}\left( t \right)$ depends on the sexual activity group. The rate of screening in both sexual activity groups is assumed to remain constant over time:

$$\begin{aligned} \eta_{L}=\omega\eta_{H}\#\left( 2 \right) \end{aligned}$$

Where 0 < ω < 1, to reflect the fact that individuals in the low-activity group seek screening less often than those in the high-activity group.

**1.2 Compartmental model equations**

The model depicted in Fig A1 is characterized by a set of differential equations and is adapted from a previous study [2]. Each compartment is stratified by sexual activity group $j\epsilon\left\{ H,L \right\}$. Full definition of model parameters is elaborated in Table A2 and Table A3.

$$\begin{aligned} \frac{dU_{j}\left( t \right)}{d_{t}}= q_{j}\alpha-\left( \lambda_{j}\left( t \right)+\gamma\right)U_{j}\left( t \right)+\nu A_{j}\left( t \right)+\rho T_{j}\left( t \right)\#\left( 3 \right) \end{aligned}$$

$$\begin{aligned} \frac{dI\left( t \right)}{d_{t}}= \lambda_{j}\left( t \right)U_{j}\left( t \right)-\left( \sigma+\gamma\right)I_{j}\left( t \right)\#\left( 4 \right) \end{aligned}$$

$$\begin{aligned} \frac{dA_{j}\left( t \right)}{d_{t}}=\left( 1-\psi\right)\sigma I_{j}\left( t \right)-\left( \nu+\eta_{j}\left( t \right)+\gamma\right)A_{j}\left( t \right)\#\left( 5 \right) \end{aligned}$$

$$\begin{aligned} \frac{dS_{j}\left( t \right)}{d_{t}}= \psi\sigma I_{j}\left( t \right)-\left( \mu+\gamma\right)S_{j}\left( t \right)\#\left( 6 \right) \end{aligned}$$

$$\begin{aligned} \frac{dT_{j}\left( t \right)}{d_{t}}= \eta_{j}\left( t \right)A_{j}\left( t \right)+\mu S_{j}\left( t \right)-\left( \rho+\gamma\right)T_{j}\left( t \right)\#\left( 7 \right) \end{aligned}$$

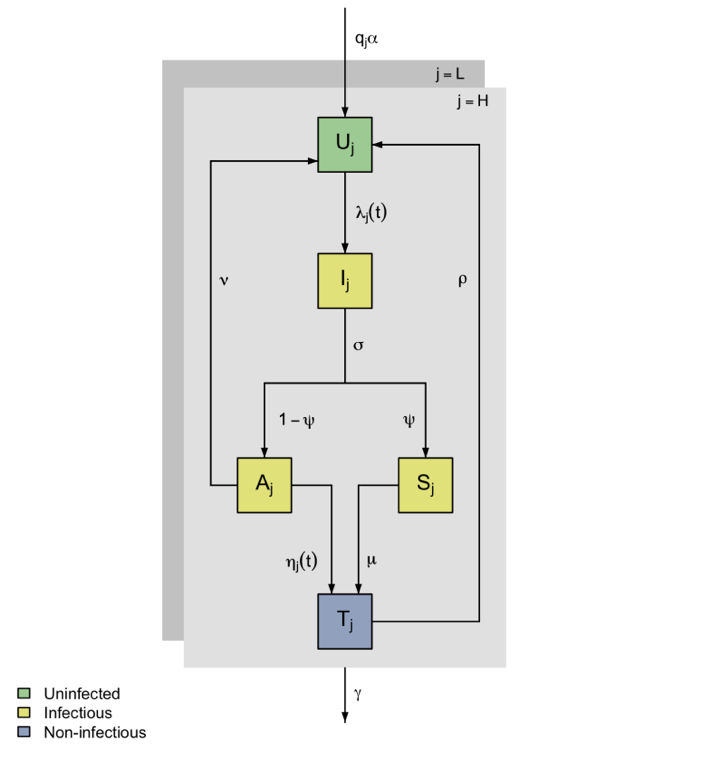


**Fig A1 Model-structure diagram for characterizing gonorrhea transmission.** The population is divided into compartments representing different states of infection. Individuals entering the sexually active population are uninfected ($U_{j}$). Individuals who become infected first pass through an incubating state ($I_{j}$), before either developing symptoms ($S_{j}$) or remain as asymptomatic individuals ($A_{j}$). Symptomatic individuals seek treatment and enter the treatment state ($T_{j}$). Asymptomatic infections can be identified through screening, with individuals entering the treatment state ($T_{j}$), or there can be natural recovery, returning individuals to the uninfected state ($U_{j}$). All treated infections are cured. Individuals leave the sexually active population through ageing from any state. Note that there are separate sets of compartments for those in the low and high sexual activity groups ($j\epsilon\left\{ H,L \right\}$ ), represented by the darker- and lighter-grey layers of the diagram, respectively, which have an identical arrangement of compartments but with different sets of parameter values; for clarity, only flows in and out of the upper layer (the high-activity group) are shown.

**2. Model calibration**

**2.1 Data source**

The publicly available data from communicable disease surveillance Singapore 2004-2020 [3] reflected the diagnosed new cases in the population among Singapore citizens, permanent residents, and temporary residents (foreign residents who have not been granted permanent residence) every year. As the actual incidence of gonorrhea in the MSM population is unknown, it was proxied using 3 separate case series, namely, (**1**) male incidence less the female incidence of gonorrhea (**2**) the male incidence of gonorrhea, i.e the upper bound to the annual gonorrhea MSM incidence (**3**) the male incidence multiplied by the proportion of reported gonorrhea cases in the MSM population in other study settings. Thereafter, the case time series of gonorrhea from each group in the period of 2004-2018, $Z_{D}\left( t \right)$, were used to seperately calibrate the transmission dynamic model. (Table A1).

**Table A1: Annual gonorrhea diagnosed in Singapore MSM under different scenarios**

| **Year** | **Main scenario**  **(Singapore MSM)** | **Upper bound**  **(Singapore Male)** | **Lower bound**  **(UK MSM portion of Singapore Male)** |
| --- | --- | --- | --- |
| 2004 | 1800 | 2209 | 1546 |
| 2005 | 1658 | 2111 | 1478 |
| 2006 | 1552 | 1967 | 1377 |
| 2007 | 1531 | 2022 | 1415 |
| 2008 | 1592 | 2012 | 1409 |
| 2009 | 1221 | 1684 | 1179 |
| 2010 | 1049 | 1433 | 1003 |
| 2011 | 1094 | 1484 | 1039 |
| 2012 | 1082 | 1400 | 980 |
| 2013 | 1037 | 1322 | 925 |
| 2014 | 1104 | 1390 | 973 |
| 2015 | 996 | 1335 | 934 |
| 2016 | 1133 | 1468 | 1027 |
| 2017 | 1474 | 1909 | 1336 |
| 2018 | 1311 | 1651 | 1155 |

**2.2 Observation process**

We used a negative binomial distribution to characterise the assumed observation process as it was count data, and hypothesis testing indicated that there was significant overdispersion in this dataset [4]. If $x$ ∼ NegBinom($m$, $\kappa$), with mean $m$ and shape parameter $\kappa$, then:

$$\begin{aligned} f_{X}\left( x | m,\kappa\right)=\frac{\Gamma\left( \kappa+x \right)}{x!\Gamma\left( \kappa\right)}\left( \frac{\kappa}{\kappa+m} \right)^{\kappa}\left( \frac{m}{\kappa+m} \right)^{x}\#\left( 8 \right) \end{aligned}$$

where the shape parameter characterises the level of clustering or heterogeneity in the observation process, and Γ(·) is the Gamma function. Under the Negative-Binomial distribution Var$(x)=m+ \frac{m^{2}}{k}$.

The likelihoods of $z_{D}(t)$ was:

$$\begin{aligned} \begin{aligned} Z_{D}\left( t \right)\sim Negbinom\left( Y_{D}\left( t \right),\kappa_{D} \right)\#(9) \end{aligned}\# \end{aligned}$$

where:

$$\begin{aligned} Y_{D}\left( t \right)=\sum_{j\in\left\{ L,H \right\}} \left( \int_{t}^{t+1} \rho T_{j}\left( \tau\right)d\tau\right)\#\left( 10 \right) \end{aligned}$$

The likelihood of the observation given the modelled trajectories produced by parameter set $\Theta$ was calculated as the product of the likelihoods of the data stream in each year t = 2004, …, 2018.

$$\begin{aligned} \begin{aligned} L\left( Z | \Theta\right)=\prod_{t=2004}^{2019} f_{D}\left( Z_{D}\left( t \right) | \Theta\right)\#(11) \end{aligned}\#\# \end{aligned}$$

**2.3 Fixed model parameters**

We calibrated the model using a previous estimate of the Singapore MSM population [5]. In line with this previous study, we fixed the sexually active population age at 15 to 65, yielding a population exit rate of $\gamma=1/50$ per year, and assumed a constant population size by allowing for $\alpha=2780$ new population entrants each year (Table A2). Due to the lack of prior knowledge on local MSM sexual behaviour, we proxied sexual attitudes using an UK MSM survey [2] and fixed the proportion of the population in the low *(*$q_{L})$and high sexual activity group ($q_{H}$) as 0.85 and 0.15 respectively [6], and the annual rate of partner change in the low and high sexual activity groups to be 0.6 and 15.6 respectively [7]. For simulating school-based vaccination programmes, we set the annual number of male adolescents aged 15 to be 19783 following Singapore demographic surveys.

**Table A2: Fixed parameters for Singapore gonorrhea transmission model**

|  | **Definition** | **Value** | **Source** |
| --- | --- | --- | --- |
| $N$ | Initial MSM population (15-65) size | 139,000 | [5] |
| $\alpha$ | Annual population entrants (at age 15) | 2780 | - |
| 1/$\gamma$ | Years spent in the sexually active population | 50 | - |
| $q_{L}$ | Proportion of the population in group L | 0.85 | [6] |
| $q_{H}$ | Proportion of the population in group H | 0.15 | [6] |
| $c_{L}$ | Annual rate of partner change in group L | 0.6 | [7] |
| $c_{H}$ | Annual rate of partner change in group H | 15.6 | [7] |
| $M$ | Male adolescents at age 15 | 19783 | [8] |

**2.4 Prior distribution of model fitted parameters**

We set priors for the model parameters that reflected existing knowledge and uncertainty about their likely range of values, as set out in Table A3.

We adopted uniform priors with a range that reflected reality for parameters whose values are highly uncertain and therefore best determined from the data, namely: the rate of per-partnership transmission in 2004 and 2018 ($\beta_{2004},\beta_{2018}$), the initial prevalence of asymptomatic gonorrhea in the low and high activity groups ($\frac{{A_{L}}_{(t0)}}{N_{L}}, \frac{{A_{H}}_{(t0)}}{N_{H}}$), the level of assortativity in sexual mixing between activity groups ($\varepsilon$), the screening rate of asymptomatic infection incidence in high activity group($\eta_{H}$), and the shape parameters relating the observation distributions ($\kappa_{D}$).

We set prior distribution values for the Singapore context using the estimated posterior distributions from a previous modelling study [2] to improve parameter calibration. These priors were for the (**1**) the screening rate in low/high sexual activity groups, (**2**) rate of natural recovery (from asymptomatic to uninfected$,$ $\nu$), (**3**) rate of leaving incubation period (from infected to symptomatic or asymptomatic$, \sigma$), (**4**) rate of seeking treatment due to symptoms (symptomatic to treated$, \mu$), (**5**) the rate of recovery after treatment (from treated to uninfected$, \rho$), and the (**6**) probability of an infectious individual becoming symptomatic ($\psi$).

**2.5 Calibration process**

We utilized Markov chain Monte Carlo (MCMC) methods to estimate the posterior distribution of the model parameters given the observed data, using a Metropolis-Hastings sampling algorithm. In each MCMC iteration, we sample a set of parameters which possibly constitute their respective posterior distributions. The proposal kernel was set as a multivariate Gaussian centered on the previous iteration’s parameter values, with a tuned covariance structure to facilitate efficient Markov chain mixing. For parameters with finite support, we set boundaries for the proposal kernel to ensure that proposal values remained epidemiologically plausible.

We parallelly ran eight independent MCMC chains for 500,000 iterations, discarding the first 1,000 iterations as burn-in. We assessed convergence by computing the Gelman-Rubin (GR) statistic for each parameter value’s MCMC chain, and deemed the MCMC convergent if GR≤1.1 for the respective inferred parameters, and that the effective sample size (ESS) for the combined chains was sufficiently large (Fig A2). We randomly selected a sample of 1,000 posterior parameter sets and fitted the model to the epidemic trajectory of gonorrhea incidence in Singapore from 2004 and 2018 as an additional verification of model fit (Fig A3).

All computations were performed in R version 4.3.1. The differential equation model was implemented using odin in gonovax package version 0.4.16 [2].

**3. Results of calibration**

Trace plots (Fig A2) and the Gelman-Rubin statistic showed that the MCMC algorithm was convergent. The calibrated model fitted annual observed diagnosed case series of gonorrhea well, with most observations falling within the range of the posterior predictive intervals (Fig A3).

**Table A3: Fitted demographic parameters: notation, definition, prior distribution, and posterior estimates.**

| **θ** | **Definition** | **Prior distribution** | **Parameter bounds** | **Posterior estimate** | | |
| --- | --- | --- | --- | --- | --- | --- |
|  |  |  |  | Main scenario | Upper bound | Lower bound |
| $\beta_{2004}$ | Probability of transmission  per-partnership in 2004 | U [0, 100%] | [0, 100%] | 44.44% (33.39%, 52.81%) | 41.05% (30.44%, 52.28%) | 39.94% (29.65%, 50.76%) |
| $\beta_{2018}$ | Probability of transmission  per-partnership in 2018 | U [0, 100%] | [0, 100%] | 44.30% (32.66%, 53.05%) | 40.21% (28.97%, 51.86%) | 39.65% (29.25%, 50.68%) |
| $A_{L}\left( t0 \right)/N_{L}$ | Initial prevalence of  asymptomatic infection in group L | U [0, 1.5%](Main)  U [0, 1.8%](Upper)  U [0, 1.3%](Lower) | [0, 1.5%](Main)  [0, 1.8%](Upper)  [0, 1.3%](Lower) | 0.74% (0.03%, 1.46%) | 0.77% (0.03%, 1.74%) | 0.55% (0.02%, 1.26%) |
| $A_{H}\left( t0 \right)/N_{H}$ | Initial prevalence of asymptomatic infection in group H | U [0, 8.6%](Main)  U [0, 10.6%](Upper)  U [0, 7.4%](Lower) | [0, 8.6%](Main)  [0, 10.6%](Upper)  [0, 7.4%](Lower) | 7.70% (5.65%, 8.57%) | 9.38% (6.99%, 10.56%) | 6.63 (4.96%, 7.37%) |
| $\varepsilon$ | Level of assortativity in sexual mixing | U [0, 1] | [0, 1] | 0.40 (0.02, 0.96) | 0.40 (0.01, 0.94) | 0.45 (0.02, 0.96) |
| $\eta_{H}$ | Rate of asymptomatic screening  in group H | U [0,4] | [0, 4] | 0.17 (0.08, 0.26) | 0.17 (0.08, 0.27) | 0.17 (0.08, 0.26) |

**Table A4: Fitted transmission parameters: notation, definition, prior distribution, parameter bounds, and posterior estimates.** Transition rate parameters ($\theta\epsilon\{\nu, \sigma, \mu,\rho\}$) are presented on an annual basis, giving a mean time to transition of $365/\theta$ days.

| **θ** | **Definition** | **Prior distribution** | **Parameter bounds** | **Posterior estimate** | | |
| --- | --- | --- | --- | --- | --- | --- |
|  |  |  |  | Main scenario | Upper bound | Lower bound |
| $\omega$ | Ratio of screening rate  in group L vs H | N(0.475, 0.168)* | [0, 1] | 0.55 (0.25, 0.89) | 0.55 (0.22, 0.87) | 0.61 (0.29, 0.93) |
| $\nu$ | Rate of natural recovery  (A → U) | N (3.08, 1.13) * | [3, 6] | 5.17 (3.75, 5.97) | 4.54 (3.24, 5.85) | 4.63 (3.33, 5.84) |
| $\sigma$ | Rate of leaving incubation period (I → S/A) | N (99.9, 30.587) * | [26, 180] | 109.62 (53.57, 163.92) | 107.48 (54.12, 166.41) | 104.35 (49.62, 161.74) |
| $\mu$ | Rate of seeking treatment  due to symptoms (S → T) | N (218, 109.21) * | [52, 365] | 217.93 (72.60, 347.18) | 216.79 (70.97, 350.30) | 221.16 (75.53, 351.02) |
| $\psi$ | Probability that incident infection is symptomatic | N (0.15, 0.0419) * | [0.1, 0.2] | 0.14 (0.11, 0.18) | 0.14 (0.10, 0.19) | 0.14 (0.11, 0.19) |
| $\rho$ | Rate of recovery after treatment  (T → U) | N (54, 5.816) * | [12, 90] | 53.99 (43.34, 65.58) | 55.42 (44.48, 66.58) | 54.84 (43.86, 65.99) |
| $\kappa_{D}$ | Shape parameter of communicable disease surveillance data | U [0, 1] | [0, 1] | 0.03 (0.01, 0.09) | 0.03 (0.01, 0.09) | 0.03 (0.01, 0.10) |

** Gonorrhea transmissional parameters’ prior distributions referring previous study in a separate UK context* [2]*.*

**(A)**

**
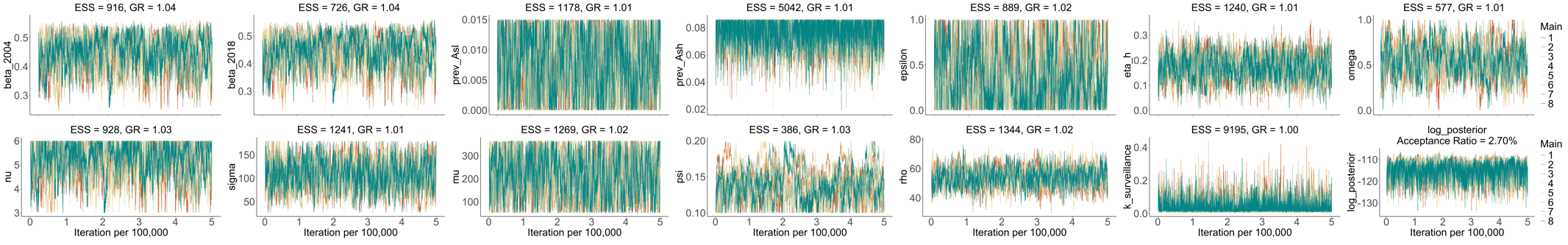
**

**(B)**

**
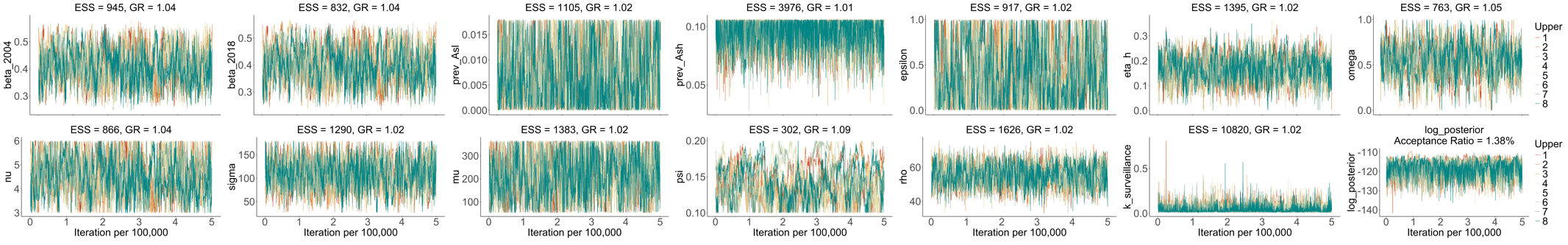
**

**(C)**


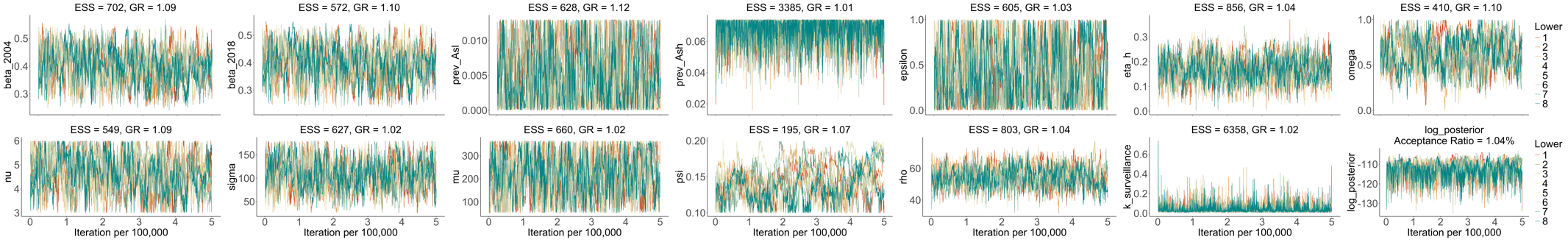


**Fig A2: Trace plots of posterior parameter estimates under different observation scenarios.** Trace plots shows the MCMC posterior parameter estimates from eight chains, each with 500,000 iterations. Colored lines represent individual chains. Effective sample size (ESS) and Gelman-Rubin (GR) diagnostics accompany each plot to quantify convergence. The acceptance rate is printed with posterior traces. The first 100,000 iterations for each chain were discarded as burn-in. Model fitted to different observation scenarios, namely, the (**1**) male incidence less the female incidence of gonorrhea (**2**) the male incidence of gonorrhea, (**3**) the male incidence multiplied by the proportion of reported gonorrhea cases in the MSM population in the UK setting.

**Fig A3: Comparison of gonorrhea annual diagnosed projection from calibrated model and observation under different scenarios.** The mean of each box is marked as a line, and the 95% credible interval is indicated by the boundaries of the box. Simulated epidemic trajectories (based on a sample of 1,000 parameter sets from the joint posterior) are in boxes, and annual gonorrhea incidence of Singapore MSM under different observation scenarios are in squares. All three observation scenarios were created based on communicable disease surveillance Singapore data. From left to right: modelled estimates and case series of (**1**) male incidence less the female incidence of gonorrhea (**2**) the male incidence of gonorrhea, (**3**) the male incidence multiplied by the proportion of reported gonorrhea cases in the MSM population in the UK setting.

**4. Simulations**

**4.1 Simulation scenarios**

Using 1,000 sets of parameters sampled from the joint posterior of the transmission model (described in Equation 3-7), we performed forward simulations of gonorrhea transmission in MSM using a previously published vaccination model [2]. As the future trajectory of the epidemic is uncertain, we considered two scenarios where gonorrhea epidemiology can develop, namely (**1**) assuming that transmission of gonorrhea in 2024 – 2034 follow the transmission of gonorrhea in 2018, i.e a constant transmissibility scenario, set at the transmission rate of the last year where the model was fitted, and (**2**) assuming that the downward trend of gonorrhea transmissibility from 2004 – 2018 continued from 2024 to 2034.

We compared the raw public health impact by number of gonorrhea cases averted; reported programme efficiency by percentage of averted cases and averted cases per dose; computed vaccine resource including number of vaccinations administered and the primary uptake ratio for each vaccination programme, over multiple levels of vaccine uptake, efficacy and duration of protection:

- Three models calibrated to

(**1**) male incidence less the female incidence of gonorrhea

(**2**) the male incidence of gonorrhea,

(**3**) the male incidence multiplied by the proportion of reported gonorrhea cases in the MSM population in the UK setting.

- Two scenarios on forward transmissibility:

(**1**) transmission of gonorrhea in 2024 – 2034 followed the transmission of gonorrhea in 2018, i.e a constant transmissibility scenario, set at the estimated transmission rate of the last year where the model was fitted

(**2**) the downward trend in gonorrhea transmissibility from 2004 – 2018 continues in 2024 – 2034

For each vaccination stratum$i\in\left\{ X, V, W \right\}$ and calendar year $t$, the total number of diagnosed cases, $Y_{D}^{i}\left( t \right)$ is given by:

$$\begin{aligned} Y_{D}^{i}\left( t \right)=\sum_{j\in\left\{ L,H \right\}} \int_{t}^{t+1} \rho T_{j}^{i}\left( \tau\right)d\tau\#\left( 14 \right) \end{aligned}$$

**4.2 Vaccination set up and calculation**

In addition to the vaccination parameters used in the main text (Table 2), we have included sensitivity analyses based on various parameters to illustrate the impact of the vaccination program under different vaccine profiles (Table A4). The efficacy of the vaccines considered were mild = 22%, normal = 31% and strong = 47%, respectively. The duration of protection was defined as a short period of 1.5 years, a normal period of 4 years and a long period of 7.5 years. Regarding the uptake rate, we simplified the simulation by assuming that each vaccinated individuals will complete the regiment dose to achieve a low overall uptake rate of 10%, a normal uptake rate of 33% and a high uptake rate of 100%.

**Table A5: Vaccination parameters used in sensitivity analysis**

|  | **Definition** | **Value** | **Source** |
| --- | --- | --- | --- |
| $e$ | Vaccine efficacy | 22% [9], 31%, 47% | [10–12] |
| $D_{v}$ | Protection of duration | 1.5, 4, 7.5 years | [13,14] |
| $p^{VbE}$ | Uptake rate of adolescent vaccination | 10%, 33%, 100% * | [15] |
| $p_{j}^{VoD}$ | Uptake of vaccination on diagnosis by infected in group j | 10%, 33%, 100% * | [15] |
| $p_{j}^{VoS}$ | Uptake of vaccination on screening by uninfected in group j | 10%, 33%, 100% * | [15] |

** The parameter values are 0 in scenarios where the mode of vaccination does not apply*

We assumed that primary vaccination required two doses, and that revaccination after protection has waned required a single booster dose. We assumed that all individuals complete their vaccination regiment. Vaccination in sexual health clinics is offered to unvaccinated MSM and those whose protection has waned, i.e. those in strata $i \in\left\{ X, W \right\}.$ We denote the total number of vaccine doses administered in each stratum in year $t$as $V^{i}\left( t \right),$ where:

$$\begin{aligned} \begin{aligned} V^{X}\left( t \right)=2\left( Mp^{VbE}+p_{j}^{VoS}Y_{U}^{X}\left( t \right)+p_{j}^{VoD}Y_{D}^{X}\left( t \right) \right)\#(15) \end{aligned} \end{aligned}$$

$$\begin{aligned} \begin{aligned} V^{W}\left( t \right)=p_{j}^{VoS}Y_{U}^{W}\left( t \right)+p_{j}^{VoD}Y_{D}^{W}\left( t \right)\#(16) \end{aligned} \end{aligned}$$

Therefore, the total number of doses administered in year $t$ across the whole population is:

$$\begin{aligned} \begin{aligned} V\left( t \right)=V^{X}\left( t \right)+V^{W}\left( t \right)\#(17) \end{aligned} \end{aligned}$$

**4.3 Comparison against baseline**

For each of the vaccines profiles set out in Table A5, we calculated the number of diagnosed cases in year $t$ across all vaccination-status strata $i\in\{X,V,W\}$, $Y_{D}\left( t \right)=\sum_{i} Y_{D}^{i}(t)$, and compare this to the baseline ($\hat{Y}_{D}(t)$) to yield the

(1) total cases averted over $M$ years starting from year $t_{0}$:

$$\begin{aligned} \sum_{t=0}^{M-1} \hat{Y}_{D}\left( t_{0}+t \right)-Y_{D}\left( t_{0}+t \right)\#\left( 18 \right) \end{aligned}$$

Similarly, using the definition set out in the Equation 17, the number of vaccine doses administered over $M$ years, relative to the baseline ($\hat{V}(t)$) is:

$$\begin{aligned} \sum_{t=0}^{M-1} V\left( t_{0}+t \right)-\hat{V}\left( t_{0}+t \right)\#\left( 19 \right) \end{aligned}$$

To reflect vaccine programme performance over $M$ years, we calculated:

(2) Averted cases by percentage, in comparison to baseline diagnosed ($\hat{Y}_{D}(t)$):

$$\begin{aligned} \sum_{t=0}^{M-1} \frac{\left( \hat{Y}_{D}\left( t_{0}+t \right)-Y_{D}\left( t_{0}+t \right) \right)}{\hat{Y}_{D}\left( t_{0}+t \right)} \times100\#\left( 20 \right) \end{aligned}$$

(3) Averted cases per dose, in comparison to baseline number of diagnosed individuals ($\hat{Y}_{D}(t)$) and vaccine doses ($\hat{V}_{D}(t)$):

$$\begin{aligned} \sum_{t=0}^{M-1} \frac{\left( \hat{Y}_{D}\left( t_{0}+t \right)-Y_{D}\left( t_{0}+t \right) \right)}{\left( V\left( t_{0}+t \right)-\hat{V}\left( t_{0}+t \right) \right)}\#\left( 21 \right) \end{aligned}$$

To characterise the vaccine resources required over $M$ years to implement each vaccination programme, we refer to individuals who were conferred primary vaccination as $\hat{V}^{X}$ and individuals who were revaccinated as $\hat{V}^{W}$. We generate the following summaries:

(4) Number of vaccinations administered:

$$\begin{aligned} \sum_{t=0}^{M-1} \frac{\left( V^{X}\left( t_{0}+t \right)-\hat{V}^{X}\left( t_{0}+t \right) \right)}{2}+\left( V^{W}\left( t_{0}+t \right)-\hat{V}^{W}\left( t_{0}+t \right) \right)\#\left( 22 \right) \end{aligned}$$

(5) Primary uptake ratio:

$$\begin{aligned} \sum_{t=0}^{M-1} \frac{\left( V^{X}\left( t_{0}+t \right)-\hat{V}^{X}\left( t_{0}+t \right) \right)}{\left( V^{X}\left( t_{0}+t \right)-\hat{V}^{X}\left( t_{0}+t \right) \right)+2\left( V^{W}\left( t_{0}+t \right)-\hat{V}^{W}\left( t_{0}+t \right) \right)}\#\left( 23 \right) \end{aligned}$$

**5. Supplementary results**

**5.1 Projections of gonorrhea epidemiology** **under decreasing transmission probability from 2024 – 2034, calibrated to case series of male incidence less the female incidence of gonorrhea**

| **Category** | **High risk** | **Low risk** | **Total** |
| --- | --- | --- | --- |
| Screened | 33933 (9006-51973) | 111550 (20961-202987) | 145483 (32075-248349) |
| Screened (%) | 24.62 (14.65-40.15) | 75.38 (43.83-84.09) |  |
| Incidence | 62335 (12011-103034) | 8236 (44-19161) | 70571 (12744-120161) |
| Incidence (%) | 88.62 (81.13-99.30) | 11.38 (0.07-18.35) |  |
| Treated | 10375 (2165-17369) | 1257 (7-2980) | 11632 (2288-19419) |
| Treated (%) | 89.37 (82.19-99.32) | 10.63 (0.07-17.46) |  |
| Asymptomatic and diagnosed | 1723 (241-3366) | 124 (0-340) | 1846 (260-3527) |
| Asymptomatic diagnosed (%) | 93.35 (84.02-99.57) | 6.65 (0.04-13.51) |  |
| Symptomatic and diagnosed | 8653 (1694-14622) | 1133 (7-2632) | 9786 (1918-16383) |
| Symptomatic and diagnosed (%) | 88.62 (81.13-99.30) | 11.38 (0.07-18.35) |  |
| Treated/Incidence (%) | 16.68 (12.25-21.05) | 15.45 (11.08-19.86) | 16.54 (12.08-20.88) |
| Asymptomatic and treated (%) | 16.88 (4.74-27.88) | 10.20 (1.69-19.87) | 16.17 (4.40-26.83) |
| Symptomatic and treated (%) | 83.12 (68.39-92.51) | 89.80 (76.84-96.73) | 83.83 (69.52-93.05) |

**Table A6 Summary of individuals entering each model compartment in the projected scenario of decreasing transmission probabilities.** Model was calibrated to case series of male incidence less the female incidence of gonorrhea. We report posterior means and accompanying 95% credible intervals in parentheses. Estimates presented were the cumulative number of individuals transitioning to each compartment in the transmission model by risk group from 2024 – 2034 under the scenario where transmission probabilities decrease linearly in 2024 – 2034 following the trend from 2004 to 2018.

| Strategy | Averted cases  (total) | Averted cases  (%) | Averted cases  per dose | Number  vaccinated | Primary  uptake ratio |
| --- | --- | --- | --- | --- | --- |
| VbE | 1601 (241-2414) | 14.03 (8.17-17.14) | 0.005 (0.001-0.007) | 172701 (172701-172701) | 100.00 (100.00-100.00) |
| VoD(H) | 1440 (76-2861) | 11.75 (3.34-15.83) | 0.24 (0.06-0.34) | 2920 (684-4711) | 96.83 (94.10-98.71) |
| VoD | 1441 (76-2864) | 11.75 (3.34-15.84) | 0.21 (0.05-0.30) | 3284 (725-5315) | 97.17 (94.57-98.85) |
| VoA | 4633 (475-8284) | 39.70 (17.54-51.82) | 0.06 (0.01-0.11) | 45700 (13639-73378) | 91.62 (85.37-95.58) |
| VoA(H) | 4613 (474-8248) | 39.53 (17.39-51.65) | 0.21 (0.03-0.33) | 11620 (5299-15811) | 87.13 (79.52-92.50) |
| VaR | 4613 (474-8249) | 39.53 (17.40-51.65) | 0.21 (0.03-0.32) | 11875 (5724-16064) | 87.40 (79.72-92.72) |

**Table A7 Summary of population health impact for various vaccination programmes from 2024 to 2034**

We report posterior means and accompanying 95% credible intervals in parentheses. Estimates presented were relative to the baseline case where transmission probabilities decrease linearly in 2024 – 2034 following the trend from 2004 to 2018.

**Fig A4 Projected number of annual diagnosed cases and evaluation metrics by vaccination programme over 2024 – 2034 with decreasing transmission probability, by model calibrated to the case series of male incidence less the female incidence of gonorrhea.** The posterior mean is marked with a square, and the 95% credible interval is indicated by the boundaries of the box. Model was calibrated to case series of the male incidence of gonorrhea. Projections assumed that transmission probabilities decrease linearly in 2024 – 2034 following the trend from 2004 to 2018. 1000 posterior draws were used to generate future projections and calculate programme-specific metrics. The series of boxplots illustrate the annual future number of diagnosed cases under different vaccination strategies compared to a no-vaccination programme baseline. Each column represented a different vaccination programme. Respectively, these were denoted (**A**) vaccination before entry [VbE], (**B**) VoD strategies by offering vaccinations in these groups to only high sexual activity individuals (VoD[H]), (**C**) vaccination on diagnoses with gonorrhea [VoD], (**D**) vaccination on attendance [VoA], (**E**) VoA strategies by offering vaccinations in these groups to only high sexual activity individuals (VoA[H]) and (**F**) vaccination according to risk [VaR].

The radar graph visualized the impact, efficiency, and uptake of each vaccination programme from 2024 – 2034. Metrics were averted cases by percentage (A.P.), averted cases per dose (A.D.), number of vaccinations administered (N.V.), and primary uptake ratio (P.U.). Each sample’s metric value was normalized by the maximum and minimum across strategies for visual clarity, points closer to the center are closer to the overall strategy minimum metric score, and the radar border represents the overall maximum score each strategy can achieve.

**5.2 Results of model calibrated to the case series of male incidence of gonorrhea, with projected transmission probabilities in 2024 – 2034 set to that of 2018**

| **Category** | **High risk** | **Low risk** | **Total** |
| --- | --- | --- | --- |
| Screened | 32805 (8376-52313) | 108971 (16245-205502) | 141776 (35567-248906) |
| Screened (%) | 24.54 (14.10-42.63) | 75.46 (39.78-84.26) |  |
| Incidence | 74134 (40422-107296) | 9805 (89-18596) | 83938 (42688-121124) |
| Incidence (%) | 88.43 (81.06-98.86) | 11.57 (0.11-18.56) |  |
| Treated | 12730 (7149-16499) | 1540 (15-2728) | 14270 (7962-18435) |
| Treated (%) | 89.24 (81.72-99.00) | 10.76 (0.11-17.58) |  |
| Asymptomatic and diagnosed | 2283 (656-3944) | 168 (1-407) | 2451 (675-4228) |
| Asymptomatic diagnosed (%) | 93.26 (82.94-99.47) | 6.74 (0.07-14.35) |  |
| Symptomatic and diagnosed | 10447 (5969-13878) | 1372 (14-2474) | 11819 (6573-15813) |
| Symptomatic and diagnosed (%) | 88.43 (81.06-98.86) | 11.57 (0.11-18.56) |  |
| Treated/Incidence (%) | 17.46 (11.82-22.34) | 16.09 (11.19-20.98) | 17.30 (11.78-22.14) |
| Asymptomatic and treated (%) | 17.99 (5.64-29.97) | 10.95 (1.33-21.31) | 17.23 (5.54-28.65) |
| Symptomatic and treated (%) | 82.01 (64.96-91.03) | 89.05 (68.57-96.47) | 82.77 (65.98-91.47) |

**Table A8 Summary of individuals entering each model compartment in projected scenario of stable transmission probabilities.** Model was calibrated to case series of the male incidence of gonorrhea. We report posterior means and accompanying 95% credible intervals in parentheses. Estimates presented were the cumulative number of individuals transitioning to each compartment in the transmission model by risk group from 2024 – 2034 under the scenario where transmission probabilities remained stable in 2024 – 2034 at 2018 levels.

| Strategy | Averted cases  (total) | Averted cases  (%) | Averted cases  per dose | Number  vaccinated | Primary  uptake ratio |
| --- | --- | --- | --- | --- | --- |
| VbE | 1665 (961-2274) | 11.76 (7.13-15.15) | 0.005 (0.003-0.007) | 172701 (172701-172701) | 100.00 (100.00-100.00) |
| VoD(H) | 1716 (644-2587) | 11.92 (7.52-15.59) | 0.24 (0.14-0.33) | 3582 (2141-4625) | 96.10 (94.03-97.10) |
| VoD | 1716 (644-2589) | 11.92 (7.52-15.59) | 0.22 (0.13-0.29) | 4031 (2388-5120) | 96.52 (94.27-97.48) |
| VoA | 5073 (1756-7653) | 35.59 (15.30-48.72) | 0.06 (0.03-0.11) | 45287 (14509-74096) | 91.56 (83.56-95.05) |
| VoA(H) | 5048 (1751-7632) | 35.42 (15.27-48.64) | 0.23 (0.14-0.30) | 11880 (5848-16328) | 86.84 (80.14-91.50) |
| VaR | 5048 (1752-7632) | 35.42 (15.27-48.64) | 0.22 (0.13-0.29) | 12211 (6161-16532) | 87.19 (80.47-91.97) |

**Table A9** **Summary of population health impact for various vaccination programmes from 2024 to 2034.**

We report posterior means and accompanying 95% credible intervals in parenthesis. Model was calibrated to case series of the male incidence of gonorrhea. Projections assumed that transmission probabilities remained stable in 2024 – 2034 at 2018 levels. We report posterior means and accompanying 95% credible intervals in parentheses.

**Fig A5 Projected number of annual diagnosed cases and evaluation metrics by vaccination programme over 2024 – 2034 with transmission probability set to that of 2018, by model calibrated to the case series of male incidence of gonorrhea.** The posterior mean is marked with a square, and the 95% credible interval is indicated by the boundaries of the box. Model was calibrated to case series of the male incidence of gonorrhea. Projections assumed that transmission probabilities remained stable in 2024 – 2034 at 2018 levels. 1000 posterior draws were used to generate future projections and calculate programme-specific metrics. The series of boxplots illustrate the annual future number of diagnosed cases under different vaccination strategies compared to a no-vaccination programme baseline. Each column represented a different vaccination programme. Respectively, these were denoted (**A**) vaccination before entry [VbE], (**B**) VoD strategies by offering vaccinations in these groups to only high sexual activity individuals (VoD[H]), (**C**) vaccination on diagnoses with gonorrhea [VoD], (**D**) vaccination on attendance [VoA], (**E**) VoA strategies by offering vaccinations in these groups to only high sexual activity individuals (VoA[H]) and (**F**) vaccination according to risk [VaR].

The radar graph visualized the impact, efficiency, and uptake of each vaccination programme from 2024 – 2034. Metrics were averted cases by percentage (A.P.), averted cases per dose (A.D.), number of vaccinations administered (N.V.), and primary uptake ratio (P.U.). Each sample’s metric value was normalized by the maximum and minimum across strategies for visual clarity, points closer to the center are closer to the overall strategy minimum metric score, and the radar border represents the overall maximum score each strategy can achieve.

**5.3 Results of model calibrated to the case series of male incidence of gonorrhea, with projected transmission probabilities in 2024 – 2034 set to decrease following the trend of 2004 to 2018**

| **Category** | **High risk** | **Low risk** | **Total** |
| --- | --- | --- | --- |
| Screened | 33160 (8562-52670) | 108997 (16251-205500) | 142157 (35800-249158) |
| Screened (%) | 24.74 (14.26-43.04) | 75.26 (39.80-83.96) |  |
| Incidence | 62993 (12831-104863) | 8383 (72-18118) | 71376 (13864-120553) |
| Incidence (%) | 88.53 (81.11-98.88) | 11.47 (0.11-18.48) |  |
| Treated | 10948 (2198-18345) | 1334 (12-3008) | 12283 (2377-20776) |
| Treated (%) | 89.33 (81.72-99.03) | 10.67 (0.10-17.51) |  |
| Asymptomatic and diagnosed | 1953 (307-3954) | 144 (1-391) | 2097 (317-4284) |
| Asymptomatic diagnosed (%) | 93.31 (82.97-99.46) | 6.69 (0.07-14.10) |  |
| Symptomatic and diagnosed | 8996 (1797-15777) | 1190 (11-2723) | 10186 (1958-17976) |
| Symptomatic and diagnosed (%) | 88.52 (81.11-98.88) | 11.48 (0.11-18.48) |  |
| Treated/Incidence (%) | 17.49 (11.84-22.32) | 16.10 (11.20-20.98) | 17.33 (11.79-22.14) |
| Asymptomatic and treated (%) | 18.08 (5.68-30.11) | 11.01 (1.34-21.40) | 17.32 (5.62-28.99) |
| Symptomatic and treated (%) | 81.92 (64.72-90.99) | 88.99 (68.33-96.45) | 82.68 (65.55-91.42) |

**Table A10 Summary of individuals entering each model compartment in projected scenario of decreasing transmission probabilities.** Model was calibrated to case series of the male incidence of gonorrhea. We report posterior means and accompanying 95% credible intervals in parentheses. Estimates presented were the cumulative number of individuals transitioning to each compartment in the transmission model by risk group from 2024 – 2034 under the scenario where transmission probabilities decrease in 2024 – 2034 following trends from 2004 to 2018.

| Strategy | Averted cases  (total) | Averted cases  (%) | Averted cases  per dose | Number  vaccinated | Primary  uptake ratio |
| --- | --- | --- | --- | --- | --- |
| VbE | 1462 (230-2374) | 12.09 (6.48-15.51) | 0.004 (0.001-0.007) | 172701 (172701-172701) | 100.00 (100.00-100.00) |
| VoD(H) | 1434 (79-3020) | 11.03 (3.29-15.81) | 0.22 (0.06-0.34) | 3101 (700-4974) | 96.65 (92.79-98.81) |
| VoD | 1435 (79-3020) | 11.04 (3.29-15.81) | 0.20 (0.05-0.30) | 3491 (757-5745) | 97.00 (93.10-98.89) |
| VoA | 4372 (481-8133) | 35.21 (13.04-47.97) | 0.05 (0.01-0.11) | 45010 (13891-74053) | 91.67 (83.61-95.33) |
| VoA(H) | 4351 (480-8100) | 35.04 (13.01-47.79) | 0.20 (0.03-0.32) | 11637 (4944-16159) | 87.11 (80.15-92.16) |
| VaR | 4351 (480-8100) | 35.04 (13.02-47.79) | 0.19 (0.03-0.31) | 11924 (5162-16501) | 87.42 (80.63-92.38) |

**Table A11 Summary of population health impact for various vaccination programmes from 2024 to 2034.**

We report posterior means and accompanying 95% credible intervals in parenthesis. Model was calibrated to case series of the male incidence of gonorrhea. Projections assumed that transmission probabilities decrease in 2024 – 2034 following trends from 2004 to 2018. We report posterior means and accompanying 95% credible intervals in parentheses.

**Fig A6 Projected number of annual diagnosed cases and evaluation metrics by vaccination programme over 2024 – 2034 with decreasing transmission probability, by model calibrated to the case series of male incidence of gonorrhea.** Posterior means are marked with a square, and the 95% credible interval is indicated by the boundaries of the box. Model was calibrated to case series of the male incidence of gonorrhea. Projections assumed that transmission probabilities decrease in 2024 – 2034 following trends from 2004 to 2018. 1000 posterior draws were used to generate future projections and calculate programme-specific metrics. The series of boxplots illustrate the annual future number of diagnosed cases under different vaccination strategies compared to a no-vaccination programme baseline. Each column represented a different vaccination programme. Respectively, these were denoted (**A**) vaccination before entry [VbE], (**B**) VoD strategies by offering vaccinations in these groups to only high sexual activity individuals (VoD[H]), (**C**) vaccination on diagnoses with gonorrhea [VoD], (**D**) vaccination on attendance [VoA], (**E**) VoA strategies by offering vaccinations in these groups to only high sexual activity individuals (VoA[H]) and (**F**) vaccination according to risk [VaR].

The radar graph visualized the impact, efficiency, and uptake of each vaccination programme from 2024 – 2034. Metrics were averted cases by percentage (A.P.), averted cases per dose (A.D.), number of vaccinations administered (N.V.), and primary uptake ratio (P.U.). Each sample’s metric value was normalized by the maximum and minimum across strategies for visual clarity, points closer to the center are closer to the overall strategy minimum metric score, and the radar border represents the overall maximum score each strategy can achieve.

**5.4 Results of model calibrated to the case series of male incidence multiplied by the proportion of reported gonorrhea cases in the UK MSM population, with projected transmission probabilities in 2024 – 2034 set to that of 2018**

| **Category** | **High risk** | **Low risk** | **Total** |
| --- | --- | --- | --- |
| Screened | 33501 (8612-51813) | 120949 (19220-221922) | 154450 (33429-268487) |
| Screened (%) | 22.83 (14.70-36.91) | 77.17 (50.06-84.69) |  |
| Incidence | 53452 (22033-75591) | 6450 (33-13220) | 59902 (25036-83551) |
| Incidence (%) | 89.37 (81.42-99.17) | 10.63 (0.06-18.33) |  |
| Treated | 9228 (3934-12653) | 1031 (5-2046) | 10258 (4294-13565) |
| Treated (%) | 90.00 (82.17-99.21) | 10.00 (0.05-17.33) |  |
| Asymptomatic and diagnosed | 1603 (422-2776) | 118 (1-317) | 1721 (464-2949) |
| Asymptomatic diagnosed (%) | 93.19 (84.63-99.60) | 6.81 (0.03-13.91) |  |
| Symptomatic and diagnosed | 7625 (3094-10732) | 912 (4-1831) | 8537 (3328-11606) |
| Symptomatic and diagnosed (%) | 89.37 (81.42-99.17) | 10.63 (0.06-18.33) |  |
| Treated/Incidence (%) | 17.48 (12.12-22.17) | 16.31 (11.34-20.83) | 17.36 (12.08-22.00) |
| Asymptomatic and treated (%) | 17.49 (4.65-26.73) | 11.54 (1.66-20.74) | 16.90 (4.41-26.24) |
| Symptomatic and treated (%) | 82.51 (69.11-91.50) | 88.46 (74.09-95.82) | 83.10 (69.48-91.84) |

**Table A12 Summary of individuals entering each model compartment in projected scenario of stable transmission probabilities.** Model was calibrated to case series of male incidence multiplied by the proportion of reported gonorrhea cases in the UK MSM population. We report posterior means and accompanying 95% credible intervals in parentheses. Estimates presented were the cumulative number of individuals transitioning to each compartment in the transmission model by risk group from 2024 – 2034 under the scenario where transmission probabilities remained stable in 2024 – 2034 at 2018 levels.

| Strategy | Averted cases  (total) | Averted cases  (%) | Averted cases  per dose | Number  vaccinated | Primary  uptake ratio |
| --- | --- | --- | --- | --- | --- |
| VbE | 1419 (567-1907) | 13.92 (9.31-17.08) | 0.004 (0.002-0.006) | 172701 (172701-172701) | 100.00 (100.00-100.00) |
| VoD(H) | 1115 (223-1773) | 10.67 (5.21-13.91) | 0.21 (0.09-0.28) | 2652 (1206-3544) | 97.12 (95.75-98.05) |
| VoD | 1115 (223-1774) | 10.67 (5.21-13.91) | 0.19 (0.08-0.25) | 2956 (1331-3839) | 97.41 (95.88-98.30) |
| VoA | 3952 (1396-6021) | 38.51 (17.56-52.06) | 0.05 (0.02-0.08) | 48013 (13583-78326) | 91.45 (84.54-95.18) |
| VoA(H) | 3935 (1392-5994) | 38.34 (17.42-51.72) | 0.19 (0.07-0.25) | 11356 (5075-15728) | 87.43 (81.27-92.21) |
| VaR | 3935 (1392-5994) | 38.34 (17.43-51.72) | 0.18 (0.07-0.24) | 11569 (5593-15904) | 87.66 (81.45-92.47) |

**Table A13 Summary of population health impact for various vaccination programmes from 2024 to 2034.**

We report posterior means and accompanying 95% credible intervals in parenthesis. Model was calibrated to case series of male incidence multiplied by the proportion of reported gonorrhea cases in the UK MSM population. Projections assumed that transmission probabilities remained stable in 2024 – 2034 at 2018 levels. We report posterior means and accompanying 95% credible intervals in parentheses.

**Fig A7 Projected number of annual diagnosed cases and evaluation metrics by vaccination programme over 2024 – 2034 with transmission probability set to that of 2018, by model calibrated to the case series of male incidence multiplied by the proportion of reported gonorrhea cases in the UK MSM population.** Posterior means are marked with a square, and the 95% credible interval are indicated by the boundaries of the box. Model was calibrated to case series of male incidence multiplied by the proportion of reported gonorrhea cases in the UK MSM population. Projections assumed that transmission probabilities remained stable in 2024 – 2034 at 2018 levels. 1000 posterior draws were used to generate future projections and calculate programme-specific metrics. The series of boxplots illustrate the annual future number of diagnosed cases under different vaccination strategies compared to a no-vaccination programme baseline. Each column represented a different vaccination programme. Respectively, these were denoted (**A**) vaccination before entry [VbE], (**B**) VoD strategies by offering vaccinations in these groups to only high sexual activity individuals (VoD[H]), (**C**) vaccination on diagnoses with gonorrhea [VoD], (**D**) vaccination on attendance [VoA], (**E**) VoA strategies by offering vaccinations in these groups to only high sexual activity individuals (VoA[H]) and (**F**) vaccination according to risk [VaR].

The radar graph visualized the impact, efficiency, and uptake of each vaccination programme from 2024 – 2034. Metrics were averted cases by percentage (A.P.), averted cases per dose (A.D.), number of vaccinations administered (N.V.), and primary uptake ratio (P.U.). Each sample’s metric value was normalized by the maximum and minimum across strategies for visual clarity, points closer to the center are closer to the overall strategy minimum metric score, and the radar border represents the overall maximum score each strategy can achieve.

**5.5 Results of model calibrated to the case series of male incidence multiplied by the proportion of reported gonorrhea cases in the UK MSM population, with projected transmission probabilities in 2024 – 2034 set to decrease following the trend of 2004 to 2018**

| **Category** | **High risk** | **Low risk** | **Total** |
| --- | --- | --- | --- |
| Screened | 33603 (8626-51967) | 120955 (19218-221923) | 154557 (33362-268778) |
| Screened (%) | 22.89 (14.56-37.00) | 77.11 (49.98-84.56) |  |
| Incidence | 50285 (5919-82118) | 6164 (24-14531) | 56450 (6215-92974) |
| Incidence (%) | 89.39 (81.38-99.17) | 10.61 (0.06-18.32) |  |
| Treated | 8779 (1011-15143) | 997 (4-2382) | 9776 (1056-16625) |
| Treated (%) | 90.02 (82.20-99.21) | 9.98 (0.05-17.34) |  |
| Asymptomatic and diagnosed | 1508 (218-3183) | 113 (0-321) | 1621 (225-3379) |
| Asymptomatic diagnosed (%) | 93.20 (84.71-99.60) | 6.80 (0.03-13.80) |  |
| Symptomatic and diagnosed | 7271 (793-12618) | 884 (3-2147) | 8155 (831-14181) |
| Symptomatic and diagnosed (%) | 89.39 (81.38-99.17) | 10.61 (0.06-18.32) |  |
| Treated/Incidence (%) | 17.50 (12.14-22.17) | 16.33 (11.35-20.82) | 17.38 (12.09-22.02) |
| Asymptomatic and treated (%) | 17.55 (4.63-26.94) | 11.59 (1.66-20.91) | 16.96 (4.42-26.35) |
| Symptomatic and treated (%) | 82.45 (69.00-91.52) | 88.41 (74.03-95.82) | 83.04 (69.37-91.82) |

**Table A14 Summary of individuals entering each model compartment in projected scenario of stable transmission probabilities.** Model was calibrated to case series of male incidence multiplied by the proportion of reported gonorrhea cases in the UK MSM population. We report posterior means and accompanying 95% credible intervals in parentheses. Estimates presented were the cumulative number of individuals transitioning to each compartment in the transmission model by risk group from 2024 – 2034 under the scenario where transmission probabilities in 2024 – 2034 set to decrease following the trend of 2004 to 2018

| Strategy | Averted cases  (total) | Averted cases  (%) | Averted cases  per dose | Number  vaccinated | Primary  uptake ratio |
| --- | --- | --- | --- | --- | --- |
| VbE | 1331 (79-2142) | 13.75 (6.62-17.20) | 0.004 (0.000-0.006) | 172701 (172701-172701) | 100.00 (100.00-100.00) |
| VoD(H) | 1071 (15-2337) | 10.17 (1.35-14.54) | 0.20 (0.02-0.30) | 2519 (330-4128) | 97.28 (94.31-99.11) |
| VoD | 1072 (15-2337) | 10.17 (1.35-14.54) | 0.18 (0.02-0.27) | 2813 (344-4599) | 97.55 (94.74-99.16) |
| VoA | 3735 (207-7211) | 37.70 (16.62-51.23) | 0.04 (0.00-0.09) | 47957 (13456-78342) | 91.47 (84.53-95.25) |
| VoA(H) | 3718 (206-7196) | 37.53 (16.54-50.90) | 0.17 (0.01-0.29) | 11305 (5190-15866) | 87.49 (81.23-92.12) |
| VaR | 3718 (206-7196) | 37.53 (16.54-50.91) | 0.17 (0.01-0.28) | 11511 (5516-16018) | 87.71 (81.42-92.34) |

**Table A15 Summary of population health impact for various vaccination programmes from 2024 to 2034.**

We report posterior means and accompanying 95% credible intervals in parenthesis. Model was calibrated to case series of male incidence multiplied by the proportion of reported gonorrhea cases in the UK MSM population. Projections assume transmission probabilities in 2024 – 2034 were set to decrease following the trend of 2004 to 2018. We report posterior means and accompanying 95% credible intervals in parentheses.

**Fig A8 Projected number of annual diagnosed cases and evaluation metrics by vaccination programme over 2024 – 2034 with decreasing transmission probability, by model calibrated to the case series of male incidence multiplied by the proportion of reported gonorrhea cases in the UK MSM population.** Posterior means are marked with a square, and the 95% credible intervals are indicated by the boundaries of the box. Model was calibrated to case series of male incidence multiplied by the proportion of reported gonorrhea cases in the UK MSM population. Projections assume transmission probabilities in 2024 – 2034 were set to decrease following the trend of 2004 to 2018. 1000 posterior draws were used to generate future projections and calculate programme-specific metrics. The series of boxplots illustrate the annual future number of diagnosed cases under different vaccination strategies compared to a no-vaccination programme baseline. Each column represented a different vaccination programme. Respectively, these were denoted (**A**) vaccination before entry [VbE], (**B**) VoD strategies by offering vaccinations in these groups to only high sexual activity individuals (VoD[H]), (**C**) vaccination on diagnoses with gonorrhea [VoD], (**D**) vaccination on attendance [VoA], (**E**) VoA strategies by offering vaccinations in these groups to only high sexual activity individuals (VoA[H]) and (**F**) vaccination according to risk [VaR].

The radar graph visualized the impact, efficiency, and uptake of each vaccination programme from 2024 – 2034. Metrics were averted cases by percentage (A.P.), averted cases per dose (A.D.), number of vaccinations administered (N.V.), and primary uptake ratio (P.U.). Each sample’s metric value was normalized by the maximum and minimum across strategies for visual clarity, points closer to the center are closer to the overall strategy minimum metric score, and the radar border represents the overall maximum score each strategy can achieve.

**6. Sensitivity analysis of using other functional forms to fit annual incidence of gonorrhea diagnoses**

We have explored using an exponentially decaying functional form for transmissibility, such that the force of infection is calculated as follows:

$$\begin{aligned} \lambda_{j}\left( t \right)=c_{j}\left( \beta_{t_{0}}+t\exp(-(\beta_{t_{1}}/\beta_{t_{0}})/ \left( t_{1}-t_{0} \right)) \right)\left( \epsilon\frac{C_{j}\left( t \right)}{N_{j}\left( t \right)}+\left( 1-\epsilon\right)\left( \sum_{i\in\left\{ L,H \right\}} \pi_{i}\left( t \right)\frac{C_{i}\left( t \right)}{N_{i}\left( t \right)} \right) \right)\#\left( 24 \right) \end{aligned}$$

and refitted the model using the same procedure as the main results on annual incidence of gonorrhea diagnoses. We found that the exponentially decaying functional form for rate of transmission did not characterise observed historical patterns well and did not use this for further evaluation of vaccination programmes post-2020.

**Fig A9:** **Comparison of gonorrhea annual diagnosed projection from calibrated model and observation under an exponentially decaying rate of transmission.** The mean of each box is marked as a line, and the 95% credible interval is indicated by the boundaries of the box. Simulated epidemic trajectories (based on a sample of 1,000 parameter sets from the joint posterior) are in boxes, and annual gonorrhea incidence of Singapore MSM are in squares.

**Sensitivity analysis on increasing rate of new entrants by 1%**

**Fig A10 Projected number of annual diagnosed cases and evaluation metrics by vaccination programme over 2024 – 2034 under hypothetically increasing number of population entrants (1% increase per year).** Posterior means are marked with a square, and the 95% credible intervals are indicated by the boundaries of the box. Model was calibrated to case series of male incidence multiplied by the proportion of reported gonorrhea cases in the UK MSM population. Projections assume transmission probabilities in 2024 – 2034 were set to decrease following the trend of 2004 to 2018. 1000 posterior draws were used to generate future projections and calculate programme-specific metrics. The series of boxplots illustrate the annual future number of diagnosed cases under different vaccination strategies compared to a no-vaccination programme baseline. Each column represented a different vaccination programme. Respectively, these were denoted (**A**) vaccination before entry [VbE], (**B**) VoD strategies by offering vaccinations in these groups to only high sexual activity individuals (VoD[H]), (**C**) vaccination on diagnoses with gonorrhea [VoD], (**D**) vaccination on attendance [VoA], (**E**) VoA strategies by offering vaccinations in these groups to only high sexual activity individuals (VoA[H]) and (**F**) vaccination according to risk [VaR].

The radar graph visualized the impact, efficiency, and uptake of each vaccination programme from 2024 – 2034. Metrics were averted cases by percentage (A.P.), averted cases per dose (A.D.), number of vaccinations administered (N.V.), and primary uptake ratio (P.U.). Each sample’s metric value was normalized by the maximum and minimum across strategies for visual clarity, points closer to the center are closer to the overall strategy minimum metric score, and the radar border represents the overall maximum score each strategy can achieve.

**Sensitivity analysis on decreasing rate of new entrants by 1%**

**Fig A11 Projected number of annual diagnosed cases and evaluation metrics by vaccination programme over 2024 – 2034 under hypothetically decreasing number of population entrants (-1% increase per year).** Posterior means are marked with a square, and the 95% credible intervals are indicated by the boundaries of the box. Model was calibrated to case series of male incidence multiplied by the proportion of reported gonorrhea cases in the UK MSM population. Projections assume transmission probabilities in 2024 – 2034 were set to decrease following the trend of 2004 to 2018. 1000 posterior draws were used to generate future projections and calculate programme-specific metrics. The series of boxplots illustrate the annual future number of diagnosed cases under different vaccination strategies compared to a no-vaccination programme baseline. Each column represented a different vaccination programme. Respectively, these were denoted (**A**) vaccination before entry [VbE], (**B**) VoD strategies by offering vaccinations in these groups to only high sexual activity individuals (VoD[H]), (**C**) vaccination on diagnoses with gonorrhea [VoD], (**D**) vaccination on attendance [VoA], (**E**) VoA strategies by offering vaccinations in these groups to only high sexual activity individuals (VoA[H]) and (**F**) vaccination according to risk [VaR].

The radar graph visualized the impact, efficiency, and uptake of each vaccination programme from 2024 – 2034. Metrics were averted cases by percentage (A.P.), averted cases per dose (A.D.), number of vaccinations administered (N.V.), and primary uptake ratio (P.U.). Each sample’s metric value was normalized by the maximum and minimum across strategies for visual clarity, points closer to the center are closer to the overall strategy minimum metric score, and the radar border represents the overall maximum score each strategy can achieve.

**Validation of model predictive accuracy in future horizons using block cross-validation**

**Fig A12: Fitted and predicted values of gonorrhea incidences from the calibrated model.** Results were generated through an expanding cross-validation scheme, where the data was split to initial testing and training sets (70%, 30%), and the model was calibrated to the training set and annual gonnorhoea incidence rates projected forward in time to assess concordance with the test-set observations. In the subsequent cross-validation steps, an additional year of data was added in and the procedure was repeated till the testing set was depleted.

| Final year of training set | Mean absolute percentage error (%) |
| --- | --- |
| 2014 | 41.05% |
| 2015 | 38.45% |
| 2016 | 38.58% |
| 2017 | 12.74% |

**Table A16: Mean absolute percentage errors of model in out-of-sample prediction of annual gonnorhea incidences.** Results were generated through an expanding cross-validation scheme, where the data was split to initial testing and training sets (70%, 30%), and the model was calibrated to the training set and annual gonnorhoea incidence rates projected forward in time to assess concordance with the test-set observations. In the subsequent cross-validation steps, an additional year of data was added in and the procedure was repeated till the testing set is depleted.

**Sensitivity analysis on varying the assortativity parameter (**$\boldsymbol{\epsilon}$**) by** $\boldsymbol{\pm}$**25 and 50% in value on the prospective impact of each vaccination strategy from 2024 – 2034**

| **Strategy** | **Averted cases**  **(total)** | **Averted cases**  **(%)** | **Averted cases**  **per dose** | **Number of vaccinations administered** | **Primary**  **uptake ratio** |
| --- | --- | --- | --- | --- | --- |
| VbE | 618  (0-1813) | 16.00  (6.32-21.78) | 0.002  (0.000-0.005) | 172701  (172701-172701) | 100.00  (100.00-100.00) |
| VoD(H) | 340  (0-1448) | 4.63  (0.00-12.35) | 0.09  (0.00-0.27) | 909  (0-2836) | 99.02  (96.29-100.00) |
| VoD | 340  (0-1449) | 4.63  (0.00-12.36) | 0.08  (0.00-0.22) | 1093  (0-3468) | 99.18  (96.93-100.00) |
| VoA | 1549  (0-5335) | 37.53  (11.56-54.56) | 0.02  (0.00-0.07) | 44641  (12405-72189) | 92.00  (85.52-96.17) |
| VoA(H) | 1539  (0-5292) | 37.29  (11.49-54.36) | 0.08  (0.00-0.24) | 10665  (3813-15111) | 88.20  (80.25-93.77) |
| VaR | 1539  (0-5292) | 37.30  (11.49-54.36) | 0.07  (0.00-0.23) | 10786  (3835-15282) | 88.33  (80.29-93.99) |

**Table A17 Summary of population health impact for various vaccination programmes from 2024 to 2034, with a -50% change in the assortativity** **parameter.** We report posterior means and accompanying 95% credible intervals. Estimates presented were relative to the baseline scenario of no intervention, with transmission probabilities from 2024 to 2034 kept stable at 2018 levels.

| **Strategy** | **Averted cases**  **(total)** | **Averted cases**  **(%)** | **Averted cases**  **per dose** | **Number of vaccinations administered** | **Primary**  **uptake ratio** |
| --- | --- | --- | --- | --- | --- |
| VbE | 948  (11-1900) | 17.50  (10.75-21.81) | 0.003  (0.000-0.006) | 172701  (172701-172701) | 100.00  (100.00-100.00) |
| VoD(H) | 549  (0-1635) | 7.17  (0.17-12.97) | 0.14  (0.00-0.28) | 1434  (22-3040) | 98.45  (96.12-99.93) |
| VoD | 549  (0-1636) | 7.18  (0.17-12.98) | 0.12  (0.00-0.23) | 1693  (23-3674) | 98.68  (96.78-99.94) |
| VoA | 2409  (23-5596) | 42.15  (18.31-54.51) | 0.03  (0.00-0.08) | 44906  (12652-72313) | 91.91  (85.49-96.04) |
| VoA(H) | 2395  (23-5561) | 41.95  (18.21-54.38) | 0.12  (0.00-0.25) | 10892  (4158-15231) | 87.95  (80.06-93.39) |
| VaR | 2396  (23-5562) | 41.95  (18.22-54.38) | 0.11  (0.00-0.24) | 11062  (4376-15475) | 88.13  (80.16-93.73) |

**Table A18 Summary of population health impact for various vaccination programmes from 2024 to 2034, with a -25% change in the assortativity** **parameter.** We report posterior means and accompanying 95% credible intervals. Estimates presented were relative to the baseline scenario of no intervention, with transmission probabilities from 2024 to 2034 kept stable at 2018 levels.

| **Strategy** | **Averted cases**  **(total)** | **Averted cases**  **(%)** | **Averted cases**  **per dose** | **Number of vaccinations administered** | **Primary**  **uptake ratio** |
| --- | --- | --- | --- | --- | --- |
| VbE | 1906  (1067-2530) | 11.37  (7.04-15.70) | 0.006  (0.003-0.007) | 172701  (172701-172701) | 100.00  (100.00-100.00) |
| VoD(H) | 2426  (713-4395) | 13.71  (7.97-17.66) | 0.28  (0.15-0.37) | 4364  (1945-6481) | 95.24  (92.28-97.22) |
| VoD | 2426  (714-4395) | 13.71  (7.97-17.66) | 0.26  (0.14-0.35) | 4722  (2324-6605) | 95.57  (92.35-97.67) |
| VoA | 6265  (2461-9736) | 36.58  (17.83-49.13) | 0.07  (0.03-0.13) | 46535  (13603-74063) | 91.26  (85.12-94.92) |
| VoA(H) | 6246  (2461-9716) | 36.46  (17.67-48.87) | 0.27  (0.15-0.36) | 12450  (6331-16380) | 86.19  (78.89-91.45) |
| VaR | 6246  (2461-9716) | 36.46  (17.68-48.87) | 0.26  (0.14-0.36) | 12712  (6563-16554) | 86.47  (79.04-91.91) |

**Table A19 Summary of population health impact for various vaccination programmes from 2024 to 2034, with a +25% change in the assortativity** **parameter** We report posterior means and accompanying 95% credible intervals. Estimates presented were relative to the baseline scenario of no intervention, with transmission probabilities from 2024 to 2034 kept stable at 2018 levels.

| **Strategy** | **Averted cases**  **(total)** | **Averted cases**  **(%)** | **Averted cases**  **per dose** | **Number of vaccinations administered** | **Primary**  **uptake ratio** |
| --- | --- | --- | --- | --- | --- |
| VbE | 1975  (1084-2661) | 9.99  (5.96-15.25) | 0.006  (0.003-0.008) | 172701  (172701-172701) | 100.00  (100.00-100.00) |
| VoD(H) | 3053  (783-5572) | 14.25  (8.34-18.48) | 0.29  (0.16-0.39) | 5282  (2073-8035) | 94.22  (90.47-97.00) |
| VoD | 3054  (784-5572) | 14.25  (8.34-18.48) | 0.27  (0.14-0.38) | 5593  (2497-8073) | 94.51  (90.47-97.47) |
| VoA | 7021  (2461-11414) | 34.33  (16.97-47.79) | 0.08  (0.03-0.15) | 47101  (14848-74162) | 90.98  (85.07-94.72) |
| VoA(H) | 7004  (2461-11395) | 34.23  (16.97-47.68) | 0.29  (0.15-0.40) | 13035  (6473-17173) | 85.53  (78.28-91.23) |
| VaR | 7004  (2461-11396) | 34.23  (16.97-47.68) | 0.28  (0.15-0.39) | 13270  (6563-17349) | 85.78  (78.38-91.65) |

**Table A20 Summary of population health impact for various vaccination programmes from 2024 to 2034, with a +50% change in the assortativity** **parameter.** We report posterior means and accompanying 95% credible intervals. Estimates presented were relative to the baseline scenario of no intervention, with transmission probabilities from 2024 to 2034 kept stable at 2018 levels.

| **Strategy** | **-50%** | **-25%** | **Main analysis** | **+25%** | **+50%** |
| --- | --- | --- | --- | --- | --- |
| VbE | 618  (0-1813) | 948  (11-1900) | 1648 (942-2120) | 1906  (1067-2530) | 1975  (1084-2661) |
| VoD(H) | 340  (0-1448) | 549  (0-1635) | 1427 (551-2176) | 2426  (713-4395) | 3053  (783-5572) |
| VoD | 340  (0-1449) | 549  (0-1636) | 1427 (551-2176) | 2426  (714-4395) | 3054  (784-5572) |
| VoA | 1549  (0-5335) | 2409  (23-5596) | 4704 (1948-6947) | 6265  (2461-9736) | 7021  (2461-11414) |
| VoA(H) | 1539  (0-5292) | 2395  (23-5561) | 4685 (1938-6922) | 6246  (2461-9716) | 7004  (2461-11395) |
| VaR | 1539  (0-5292) | 2396  (23-5562) | 4685 (1939-6922) | 6246  (2461-9716) | 7004  (2461-11396) |

**Table A21 Summary of averted cases (total)** **for various vaccination programmes from 2024 to 2034, by varying the assortativity parameter (**$\boldsymbol{\epsilon}$**).** We report posterior means and accompanying 95% credible intervals. Estimates presented were relative to the baseline scenario of no intervention, with transmission probabilities from 2024 to 2034 kept stable at 2018 levels.

| **Strategy** | **-50%** | **-25%** | **Main analysis** | **+25%** | **+50%** |
| --- | --- | --- | --- | --- | --- |
| VbE | 16.00  (6.32-21.78) | 17.50  (10.75-21.81) | 14.18  (9.11-17.08) | 11.37  (7.04-15.70) | 9.99  (5.96-15.25) |
| VoD(H) | 4.63  (0.00-12.35) | 7.17  (0.17-12.97) | 12.04  (7.12-15.00) | 13.71  (7.97-17.66) | 14.25  (8.34-18.48) |
| VoD | 4.63  (0.00-12.36) | 7.18  (0.17-12.98) | 12.04  (7.12-15.00) | 13.71  (7.97-17.66) | 14.25  (8.34-18.48) |
| VoA | 37.53  (11.56-54.56) | 42.15  (18.31-54.51) | 40.26  (18.32-52.57) | 36.58  (17.83-49.13) | 34.33  (16.97-47.79) |
| VoA(H) | 37.29  (11.49-54.36) | 41.95  (18.21-54.38) | 40.10  (18.14-52.55) | 36.46  (17.67-48.87) | 34.23  (16.97-47.68) |
| VaR | 37.30  (11.49-54.36) | 41.95  (18.22-54.38) | 40.10  (18.14-52.55) | 36.46  (17.68-48.87) | 34.23  (16.97-47.68) |

**Table A22 Summary of averted cases by percentage** **for various vaccination programmes from 2024 to 2034, by varying the assortativity parameter (**$\boldsymbol{\epsilon}$**).** We report posterior means and accompanying 95% credible intervals. Estimates presented were relative to the baseline scenario of no intervention, with transmission probabilities from 2024 to 2034 kept stable at 2018 levels.

| **Strategy** | **-50%** | **-25%** | **Main analysis** | **+25%** | **+50%** |
| --- | --- | --- | --- | --- | --- |
| VbE | 0.002  (0.000-0.005) | 0.003  (0.000-0.006) | 0.005  (0.003-0.006) | 0.006  (0.003-0.007) | 0.006  (0.003-0.008) |
| VoD(H) | 0.09  (0.00-0.27) | 0.14  (0.00-0.28) | 0.24  (0.14-0.31) | 0.28  (0.15-0.37) | 0.29  (0.16-0.39) |
| VoD | 0.08  (0.00-0.22) | 0.12  (0.00-0.23) | 0.22  (0.12-0.28) | 0.26  (0.14-0.35) | 0.27  (0.14-0.38) |
| VoA | 0.02  (0.00-0.07) | 0.03  (0.00-0.08) | 0.06  (0.02-0.10) | 0.07  (0.03-0.13) | 0.08  (0.03-0.15) |
| VoA(H) | 0.08  (0.00-0.24) | 0.12  (0.00-0.25) | 0.22  (0.12-0.28) | 0.27  (0.15-0.36) | 0.29  (0.15-0.40) |
| VaR | 0.07  (0.00-0.23) | 0.11  (0.00-0.24) | 0.21 (0.12-0.27) | 0.26  (0.14-0.36) | 0.28  (0.15-0.39) |

**Table A23 Summary of averted cases per dose** **for various vaccination programmes from 2024 to 2034, by varying the assortativity parameter (**$\boldsymbol{\epsilon}$**).** We report posterior means and accompanying 95% credible intervals. Estimates presented were relative to the baseline scenario of no intervention, with transmission probabilities from 2024 to 2034 kept stable at 2018 levels.

**Sensitivity analysis on varying the screening rate of asymptomatics in high risk group(**$\boldsymbol{\eta}_{\boldsymbol{h}}$**) by** $\boldsymbol{\pm}$**25 and 50% in value on the prospective impact of each vaccination strategy from 2024 – 2034**

| **Strategy** | **Averted cases**  **(total)** | **Averted cases**  **(%)** | **Averted cases**  **per dose** | **Number of vaccinations administered** | **Primary**  **uptake ratio** |
| --- | --- | --- | --- | --- | --- |
| VbE | 1697  (955-2214) | 12.51  (8.18-15.21) | 0.005  (0.003-0.006) | 172701  (172701-172701) | 100.00  (100.00-100.00) |
| VoD(H) | 1659  (693-2507) | 12.03  (7.26-15.14) | 0.25  (0.14-0.33) | 3386  (2088-4295) | 96.31  (94.96-97.27) |
| VoD | 1659  (693-2507) | 12.03  (7.26-15.14) | 0.22  (0.12-0.28) | 3849  (2337-4864) | 96.73  (95.10-97.65) |
| VoA | 3621  (1540-5478) | 26.39  (14.12-33.96) | 0.05  (0.02-0.08) | 41970  (11478-68276) | 92.90  (86.56-96.46) |
| VoA(H) | 3589  (1529-5413) | 26.16  (13.92-33.79) | 0.24  (0.14-0.31) | 7788  (4174-10286) | 91.43  (86.96-94.48) |
| VaR | 3590  (1529-5414) | 26.17  (13.92-33.79) | 0.23  (0.13-0.30) | 8179  (4668-10709) | 91.83  (87.47-94.77) |

**Table A24 Summary of population health impact for various vaccination programmes from 2024 to 2034, with a -50% change in the asymptomatic screening rate**. We report posterior means and accompanying 95% credible intervals. Estimates presented were relative to the baseline scenario of no intervention, with transmission probabilities from 2024 to 2034 kept stable at 2018 levels.

| **Strategy** | **Averted cases**  **(total)** | **Averted cases**  **(%)** | **Averted cases**  **per dose** | **Number of vaccinations administered** | **Primary**  **uptake ratio** |
| --- | --- | --- | --- | --- | --- |
| VbE | 1685  (973-2183) | 13.30  (8.62-16.14) | 0.005  (0.003-0.006) | 172701  (172701-172701) | 100.00  (100.00-100.00) |
| VoD(H) | 1561  (625-2370) | 12.11  (7.42-15.13) | 0.25  (0.14-0.32) | 3187  (1918-4096) | 96.54  (95.22-97.44) |
| VoD | 1561  (626-2371) | 12.11  (7.42-15.13) | 0.22  (0.12-0.28) | 3600  (2106-4536) | 96.91  (95.36-97.80) |
| VoA | 4287  (1799-6396) | 33.56  (16.35-43.86) | 0.05  (0.02-0.09) | 43861  (12061-70829) | 92.32  (86.06-96.03) |
| VoA(H) | 4262  (1790-6367) | 33.37  (16.17-43.65) | 0.23  (0.13-0.30) | 9735  (4872-13042) | 89.25  (83.17-93.34) |
| VaR | 4263  (1790-6368) | 33.37  (16.18-43.65) | 0.22  (0.13-0.29) | 10051  (5254-13387) | 89.58  (83.46-93.66) |

**Table A25 Summary of population health impact for various vaccination programmes from 2024 to 2034, with a -25% change in the asymptomatic screening rate**. We report posterior means and accompanying 95% credible intervals. Estimates presented were relative to the baseline scenario of no intervention, with transmission probabilities from 2024 to 2034 kept stable at 2018 levels.

| **Strategy** | **Averted cases**  **(total)** | **Averted cases**  **(%)** | **Averted cases**  **per dose** | **Number of vaccinations administered** | **Primary**  **uptake ratio** |
| --- | --- | --- | --- | --- | --- |
| VbE | 1579  (856-2058) | 15.13  (9.65-18.34) | 0.005  (0.002-0.006) | 172701  (172701-172701) | 100.00  (100.00-100.00) |
| VoD(H) | 1260  (372-1969) | 11.77  (6.74-14.71) | 0.23  (0.13-0.30) | 2687  (1360-3698) | 97.09  (95.59-98.01) |
| VoD | 1260  (372-1970) | 11.78  (6.74-14.71) | 0.21  (0.11-0.27) | 3001  (1508-3994) | 97.38  (95.80-98.25) |
| VoA | 4837  (2031-6885) | 46.25  (20.41-59.58) | 0.06  (0.02-0.10) | 47488  (14086-75559) | 90.82  (84.49-95.06) |
| VoA(H) | 4823  (2022-6878) | 46.11  (20.29-59.50) | 0.20  (0.10-0.27) | 13452  (6086-18205) | 85.06  (76.15-91.24) |
| VaR | 4823  (2022-6878) | 46.11  (20.29-59.50) | 0.19  (0.10-0.26) | 13650  (6463-18359) | 85.28  (76.28-91.53) |

**Table A26 Summary of population health impact for various vaccination programmes from 2024 to 2034, with a +25% change in the asymptomatic screening rate**. We report posterior means and accompanying 95% credible intervals. Estimates presented were relative to the baseline scenario of no intervention, with transmission probabilities from 2024 to 2034 kept stable at 2018 levels.

| **Strategy** | **Averted cases**  **(total)** | **Averted cases**  **(%)** | **Averted cases**  **per dose** | **Number of vaccinations administered** | **Primary**  **uptake ratio** |
| --- | --- | --- | --- | --- | --- |
| VbE | 1474  (639-1979) | 16.16  (10.14-19.68) | 0.004  (0.002-0.006) | 172701  (172701-172701) | 100.00  (100.00-100.00) |
| VoD(H) | 1067  (227-1774) | 11.26  (5.75-14.22) | 0.22  (0.10-0.29) | 2387  (1030-3429) | 97.42  (95.78-98.45) |
| VoD | 1067  (227-1775) | 11.26  (5.75-14.23) | 0.20  (0.09-0.26) | 2655  (1149-3725) | 97.66  (96.06-98.63) |
| VoA | 4688  (1933-6723) | 51.40  (22.72-64.48) | 0.05  (0.02-0.10) | 49224  (15113-77844) | 89.96  (83.50-94.48) |
| VoA(H) | 4677  (1925-6712) | 51.28  (22.60-64.35) | 0.17  (0.06-0.25) | 15222  (6663-20682) | 83.06  (73.03-90.26) |
| VaR | 4677  (1925-6712) | 51.29  (22.60-64.35) | 0.17  (0.06-0.24) | 15375  (7018-20739) | 83.23  (73.11-90.47) |

**Table A27 Summary of population health impact for various vaccination programmes from 2024 to 2034, with a +50% change in the asymptomatic screening rate**. We report posterior means and accompanying 95% credible intervals. Estimates presented were relative to the baseline scenario of no intervention, with transmission probabilities from 2024 to 2034 kept stable at 2018 levels.

**Sensitivity analysis on varying the rate of seeking treatment due to symptoms** **(**$\boldsymbol{\mu}$**) by** $\boldsymbol{\pm}$**25 and 50% in value on the prospective impact of each vaccination strategy from 2024 – 2034**

| **Strategy** | **Averted cases**  **(total)** | **Averted cases**  **(%)** | **Averted cases**  **per dose** | **Number of vaccinations administered** | **Primary**  **uptake ratio** |
| --- | --- | --- | --- | --- | --- |
| VbE | 1713  (994-2236) | 13.67  (8.99-16.36) | 0.005  (0.003-0.006) | 172701  (172701-172701) | 100.00  (100.00-100.00) |
| VoD(H) | 1582  (620-2431) | 12.38  (7.36-15.51) | 0.25  (0.14-0.33) | 3160  (1870-4117) | 96.57  (95.12-97.51) |
| VoD | 1583  (620-2431) | 12.39  (7.36-15.52) | 0.22  (0.12-0.29) | 3552  (2135-4523) | 96.93  (95.38-97.83) |
| VoA | 5001  (2219-7304) | 39.70  (18.16-52.19) | 0.06  (0.03-0.10) | 45830  (13149-73427) | 91.57  (85.34-95.50) |
| VoA(H) | 4980  (2215-7298) | 39.54  (18.01-52.17) | 0.23  (0.14-0.30) | 11736  (5676-15792) | 87.00  (79.48-92.16) |
| VaR | 4980  (2215-7298) | 39.54  (18.01-52.17) | 0.22  (0.13-0.29) | 12010  (6063-15959) | 87.29  (79.69-92.57) |

**Table A28 Summary of population health impact for various vaccination programmes from 2024 to 2034, with a -50% change in the rate of seeking treatment due to symptoms**. We report posterior means and accompanying 95% credible intervals. Estimates presented were relative to the baseline scenario of no intervention, with transmission probabilities from 2024 to 2034 kept stable at 2018 levels.

| **Strategy** | **Averted cases**  **(total)** | **Averted cases**  **(%)** | **Averted cases**  **per dose** | **Number of vaccinations administered** | **Primary**  **uptake ratio** |
| --- | --- | --- | --- | --- | --- |
| VbE | 1671  (974-2163) | 14.00  (9.07-16.84) | 0.005  (0.003-0.006) | 172701  (172701-172701) | 100.00  (100.00-100.00) |
| VoD(H) | 1479  (574-2265) | 12.16  (7.24-15.18) | 0.25  (0.14-0.32) | 3023  (1745-3983) | 96.72  (95.35-97.64) |
| VoD | 1480  (574-2265) | 12.17  (7.24-15.18) | 0.22  (0.12-0.28) | 3396  (1963-4356) | 97.06  (95.53-97.91) |
| VoA | 4805  (2074-7124) | 40.08  (18.26-52.45) | 0.06  (0.03-0.10) | 45743  (13053-73360) | 91.60  (85.35-95.55) |
| VoA(H) | 4786  (2066-7095) | 39.91  (18.08-52.43) | 0.22  (0.13-0.29) | 11660  (5565-15700) | 87.09  (79.52-92.20) |
| VaR | 4786  (2066-7095) | 39.91  (18.09-52.43) | 0.21  (0.12-0.28) | 11919  (5959-15879) | 87.36  (79.72-92.60) |

**Table A29 Summary of population health impact for various vaccination programmes from 2024 to 2034, with a -25% change in the rate of seeking treatment due to symptoms.** We report posterior means and accompanying 95% credible intervals. Estimates presented were relative to the baseline scenario of no intervention, with transmission probabilities from 2024 to 2034 kept stable at 2018 levels.

| **Strategy** | **Averted cases**  **(total)** | **Averted cases**  **(%)** | **Averted cases**  **per dose** | **Number of vaccinations administered** | **Primary**  **uptake ratio** |
| --- | --- | --- | --- | --- | --- |
| VbE | 1633  (930-2101) | 14.28  (9.13-17.36) | 0.005  (0.003-0.006) | 172701  (172701-172701) | 100.00  (100.00-100.00) |
| VoD(H) | 1396  (528-2122) | 11.96  (7.02-14.81) | 0.24  (0.14-0.31) | 2912  (1656-3862) | 96.84  (95.54-97.75) |
| VoD | 1396  (528-2123) | 11.97  (7.02-14.81) | 0.21  (0.12-0.28) | 3270  (1809-4205) | 97.17  (95.67-98.04) |
| VoA | 4642  (1906-6825) | 40.37  (18.35-52.69) | 0.06  (0.02-0.10) | 45673  (12975-73308) | 91.63  (85.35-95.58) |
| VoA(H) | 4623  (1895-6803) | 40.21  (18.17-52.62) | 0.21  (0.12-0.28) | 11600  (5458-15646) | 87.15  (79.56-92.26) |
| VaR | 4623  (1896-6803) | 40.21  (18.17-52.62) | 0.21  (0.12-0.27) | 11847  (5875-15822) | 87.42  (79.74-92.62) |

**Table A30 Summary of population health impact for various vaccination programmes from 2024 to 2034, with a +25% change in the rate of seeking treatment due to symptoms.** We report posterior means and accompanying 95% credible intervals. Estimates presented were relative to the baseline scenario of no intervention, with transmission probabilities from 2024 to 2034 kept stable at 2018 levels.

| **Strategy** | **Averted cases**  **(total)** | **Averted cases**  **(%)** | **Averted cases**  **per dose** | **Number of vaccinations administered** | **Primary**  **uptake ratio** |
| --- | --- | --- | --- | --- | --- |
| VbE | 1623  (925-2088) | 14.35  (9.15-17.50) | 0.005  (0.003-0.006) | 172701  (172701-172701) | 100.00  (100.00-100.00) |
| VoD(H) | 1375  (508-2096) | 11.91  (7.00-14.75) | 0.24  (0.14-0.31) | 2884  (1644-3828) | 96.87  (95.57-97.79) |
| VoD | 1375  (508-2097) | 11.91  (7.01-14.75) | 0.21  (0.12-0.27) | 3238  (1779-4183) | 97.19  (95.69-98.06) |
| VoA | 4600  (1879-6771) | 40.44  (18.37-52.87) | 0.06  (0.02-0.10) | 45656  (12955-73294) | 91.63  (85.35-95.59) |
| VoA(H) | 4581  (1868-6732) | 40.28  (18.19-52.85) | 0.21  (0.12-0.28) | 11585  (5428-15639) | 87.17  (79.56-92.28) |
| VaR | 4582  (1868-6732) | 40.28  (18.20-52.85) | 0.21  (0.12-0.27) | 11829  (5854-15811) | 87.43  (79.75-92.64) |

**Table A31 Summary of population health impact for various vaccination programmes from 2024 to 2034, with a +50% change in the rate of seeking treatment due to symptoms.** We report posterior means and accompanying 95% credible intervals. Estimates presented were relative to the baseline scenario of no intervention, with transmission probabilities from 2024 to 2034 kept stable at 2018 levels.

**Sensitivity analysis on varying the natural recovery rate**

**(**$\boldsymbol{\nu}$**) by** $\boldsymbol{\pm}$**25 and 50% in value on the prospective impact of each vaccination strategy from 2024 – 2034**

| **Strategy** | **Averted cases**  **(total)** | **Averted cases**  **(%)** | **Averted cases**  **per dose** | **Number of vaccinations administered** | **Primary**  **uptake ratio** |
| --- | --- | --- | --- | --- | --- |
| VbE | 1558  (863-2117) | 2.42  (1.66-4.35) | 0.005  (0.002-0.006) | 172701  (172701-172701) | 100.00  (100.00-100.00) |
| VoD(H) | 5936  (1920-9080) | 8.83  (5.87-11.84) | 0.22  (0.13-0.30) | 14485  (5392-18299) | 83.87  (78.00-90.42) |
| VoD | 5965  (1921-9157) | 8.87  (5.92-11.85) | 0.18  (0.11-0.24) | 17783  (5667-24148) | 86.61  (79.93-92.09) |
| VoA | 8076  (3219-12387) | 12.25  (7.25-19.49) | 0.08  (0.04-0.13) | 55061  (24732-80740) | 87.81  (83.60-91.22) |
| VoA(H) | 7837  (3183-11850) | 11.92  (7.04-19.03) | 0.23  (0.13-0.31) | 18840  (11421-22816) | 78.93  (72.88-84.83) |
| VaR | 7865  (3184-11906) | 11.96  (7.05-19.05) | 0.20  (0.12-0.27) | 22047  (12002-27912) | 81.77  (75.14-86.84) |

**Table A32 Summary of population health impact for various vaccination programmes from 2024 to 2034, with a -50% change in the natural recovery rate.** We report posterior means and accompanying 95% credible intervals. Estimates presented were relative to the baseline scenario of no intervention, with transmission probabilities from 2024 to 2034 kept stable at 2018 levels.

| **Strategy** | **Averted cases**  **(total)** | **Averted cases**  **(%)** | **Averted cases**  **per dose** | **Number of vaccinations administered** | **Primary**  **uptake ratio** |
| --- | --- | --- | --- | --- | --- |
| VbE | 1943  (1112-2585) | 3.88  (3.24-4.35) | 0.006  (0.003-0.007) | 172701  (172701-172701) | 100.00  (100.00-100.00) |
| VoD(H) | 6114  (1920-9545) | 11.98  (7.95-14.99) | 0.28  (0.16-0.38) | 11297  (5392-13641) | 87.49  (83.89-90.72) |
| VoD | 6124  (1921-9559) | 12.00  (7.97-15.01) | 0.24  (0.14-0.32) | 13239  (5667-16554) | 89.20  (85.15-92.22) |
| VoA | 9420  (3533-14186) | 18.60  (10.75-23.58) | 0.10  (0.04-0.16) | 52253  (22566-78177) | 88.89  (84.15-91.74) |
| VoA(H) | 9287  (3492-14090) | 18.34  (10.63-23.38) | 0.30  (0.17-0.40) | 17006  (11006-20542) | 81.02  (75.18-85.65) |
| VaR | 9296  (3493-14094) | 18.36  (10.64-23.39) | 0.27  (0.16-0.35) | 18815  (11772-22841) | 82.76  (76.68-87.40) |

**Table A33 Summary of population health impact for various vaccination programmes from 2024 to 2034, with a -25% change in the natural recovery rate.** We report posterior means and accompanying 95% credible intervals. Estimates presented were relative to the baseline scenario of no intervention, with transmission probabilities from 2024 to 2034 kept stable at 2018 levels.

| **Strategy** | **Averted cases**  **(total)** | **Averted cases**  **(%)** | **Averted cases**  **per dose** | **Number of vaccinations administered** | **Primary**  **uptake ratio** |
| --- | --- | --- | --- | --- | --- |
| VbE | 316  (0-1782) | 11.42  (2.56-22.59) | 0.001  (0.000-0.005) | 172701  (172701-172701) | 100.00  (100.00-100.00) |
| VoD(H) | 182  (0-1428) | 2.53  (0.06-12.91) | 0.05  (0.00-0.27) | 447  (0-2782) | 99.52  (96.24-100.00) |
| VoD | 182  (0-1429) | 2.53  (0.06-12.91) | 0.04  (0.00-0.23) | 505  (0-3156) | 99.57  (96.56-100.00) |
| VoA | 788  (0-4949) | 26.56  (4.30-54.28) | 0.01  (0.00-0.06) | 44372  (10543-72141) | 92.09  (85.53-96.48) |
| VoA(H) | 785  (0-4918) | 26.45  (4.24-54.17) | 0.04  (0.00-0.23) | 10462  (3078-14869) | 88.43  (80.20-94.45) |
| VaR | 785  (0-4919) | 26.45  (4.24-54.17) | 0.04  (0.00-0.22) | 10498  (3078-14869) | 88.47  (80.20-94.56) |

**Table A34 Summary of population health impact for various vaccination programmes from 2024 to 2034, with a +25% change in the natural recovery rate.** We report posterior means and accompanying 95% credible intervals. Estimates presented were relative to the baseline scenario of no intervention, with transmission probabilities from 2024 to 2034 kept stable at 2018 levels.

| **Strategy** | **Averted cases**  **(total)** | **Averted cases**  **(%)** | **Averted cases**  **per dose** | **Number of vaccinations administered** | **Primary**  **uptake ratio** |
| --- | --- | --- | --- | --- | --- |
| VbE | 316  (0-1782) | 11.07  (20.97-22.68) | 0.001  (0.000-0.005) | 172701  (172701-172701) | 100.00  (100.00-100.00) |
| VoD(H) | 182  (0-1428) | 2.53  (0.00-12.91) | 0.05  (0.00-0.27) | 447  (0-2782) | 99.52  (96.24-100.00) |
| VoD | 182  (0-1429) | 2.53  (0.00-12.91) | 0.04  (0.00-0.23) | 505  (0-3156) | 99.57  (96.56-100.00) |
| VoA | 788  (0-4949) | 24.64  (0.00-54.28) | 0.01  (0.00-0.06) | 44372  (10543-72141) | 92.09  (85.53-96.48) |
| VoA(H) | 785  (0-4918) | 24.55  (0.00-54.17) | 0.04  (0.00-0.23) | 10462  (3078-14869) | 88.43  (80.20-94.45) |
| VaR | 785  (0-4919) | 24.54  (0.00-54.17) | 0.04  (0.00-0.22) | 10498  (3078-14869) | 88.47  (80.20-94.56) |

**Table A35 Summary of population health impact for various vaccination programmes from 2024 to 2034, with a +50% change in the natural recovery rate.** We report posterior means and accompanying 95% credible intervals. Estimates presented were relative to the baseline scenario of no intervention, with transmission probabilities from 2024 to 2034 kept stable at 2018 levels.

**Sensitivity analysis on varying the screening rate in low versus high groups**$\boldsymbol{\omega}$**) by** $\boldsymbol{\pm}$**25 and 50% in value on the prospective impact of each vaccination strategy from 2024 – 2034**

| **Strategy** | **Averted cases**  **(total)** | **Averted cases**  **(%)** | **Averted cases**  **per dose** | **Number of vaccinations administered** | **Primary**  **uptake ratio** |
| --- | --- | --- | --- | --- | --- |
| VbE | 1713  (994-2236) | 13.67  (8.99-16.36) | 0.005  (0.003-0.006) | 172701  (172701-172701) | 100.00  (100.00-100.00) |
| VoD(H) | 1582  (620-2431) | 12.38  (7.36-15.51) | 0.25  (0.14-0.33) | 3160  (1870-4117) | 96.57  (95.12-97.51) |
| VoD | 1583  (620-2431) | 12.39  (7.36-15.52) | 0.22  (0.12-0.29) | 3552  (2135-4523) | 96.93  (95.38-97.83) |
| VoA | 5001  (2219-7304) | 39.70  (18.16-52.19) | 0.06  (0.03-0.10) | 45830  (13149-73427) | 91.57  (85.34-95.50) |
| VoA(H) | 4980  (2215-7298) | 39.54  (18.01-52.17) | 0.23  (0.14-0.30) | 11736  (5676-15792) | 87.00  (79.48-92.16) |
| VaR | 4980  (2215-7298) | 39.54  (18.01-52.17) | 0.22  (0.13-0.29) | 12010  (6063-15959) | 87.29  (79.69-92.57) |

**Table A36 Summary of population health impact for various vaccination programmes from 2024 to 2034, with a -50% change in the screening rate in low versus high groups.** We report posterior means and accompanying 95% credible intervals. Estimates presented were relative to the baseline scenario of no intervention, with transmission probabilities from 2024 to 2034 kept stable at 2018 levels.

| **Strategy** | **Averted cases**  **(total)** | **Averted cases**  **(%)** | **Averted cases**  **per dose** | **Number of vaccinations administered** | **Primary**  **uptake ratio** |
| --- | --- | --- | --- | --- | --- |
| VbE | 1671  (974-2163) | 14.00  (9.07-16.84) | 0.005  (0.003-0.006) | 172701  (172701-172701) | 100.00  (100.00-100.00) |
| VoD(H) | 1479  (574-2265) | 12.16  (7.24-15.18) | 0.25  (0.14-0.32) | 3023  (1745-3983) | 96.72  (95.35-97.64) |
| VoD | 1480  (574-2265) | 12.17  (7.24-15.18) | 0.22  (0.12-0.28) | 3396  (1963-4356) | 97.06  (95.53-97.91) |
| VoA | 4805  (2074-7124) | 40.08  (18.26-52.45) | 0.06  (0.03-0.10) | 45743  (13053-73360) | 91.60  (85.35-95.55) |
| VoA(H) | 4786  (2066-7095) | 39.91  (18.08-52.43) | 0.22  (0.13-0.29) | 11660  (5565-15700) | 87.09  (79.52-92.20) |
| VaR | 4786  (2066-7095) | 39.91  (18.09-52.43) | 0.21  (0.12-0.28) | 11919  (5959-15879) | 87.36  (79.72-92.60) |

**Table A37 Summary of population health impact for various vaccination programmes from 2024 to 2034, with a -25% change in the rate of seeking treatment due to symptoms.** We report posterior means and accompanying 95% credible intervals. Estimates presented were relative to the baseline scenario of no intervention, with transmission probabilities from 2024 to 2034 kept stable at 2018 levels.

| **Strategy** | **Averted cases**  **(total)** | **Averted cases**  **(%)** | **Averted cases**  **per dose** | **Number of vaccinations administered** | **Primary**  **uptake ratio** |
| --- | --- | --- | --- | --- | --- |
| VbE | 1633  (930-2101) | 14.28  (9.13-17.36) | 0.005  (0.003-0.006) | 172701  (172701-172701) | 100.00  (100.00-100.00) |
| VoD(H) | 1396  (528-2122) | 11.96  (7.02-14.81) | 0.24  (0.14-0.31) | 2912  (1656-3862) | 96.84  (95.54-97.75) |
| VoD | 1396  (528-2123) | 11.97  (7.02-14.81) | 0.21  (0.12-0.28) | 3270  (1809-4205) | 97.17  (95.67-98.04) |
| VoA | 4642  (1906-6825) | 40.37  (18.35-52.69) | 0.06  (0.02-0.10) | 45673  (12975-73308) | 91.63  (85.35-95.58) |
| VoA(H) | 4623  (1895-6803) | 40.21  (18.17-52.62) | 0.21  (0.12-0.28) | 11600  (5458-15646) | 87.15  (79.56-92.26) |
| VaR | 4623  (1896-6803) | 40.21  (18.17-52.62) | 0.21  (0.12-0.27) | 11847  (5875-15822) | 87.42  (79.74-92.62) |

**Table A38 Summary of population health impact for various vaccination programmes from 2024 to 2034, with a +25% change in the rate of seeking treatment due to symptoms.** We report posterior means and accompanying 95% credible intervals. Estimates presented were relative to the baseline scenario of no intervention, with transmission probabilities from 2024 to 2034 kept stable at 2018 levels.

| **Strategy** | **Averted cases**  **(total)** | **Averted cases**  **(%)** | **Averted cases**  **per dose** | **Number of vaccinations administered** | **Primary**  **uptake ratio** |
| --- | --- | --- | --- | --- | --- |
| VbE | 1623  (925-2088) | 14.35  (9.15-17.50) | 0.005  (0.003-0.006) | 172701  (172701-172701) | 100.00  (100.00-100.00) |
| VoD(H) | 1375  (508-2096) | 11.91  (7.00-14.75) | 0.24  (0.14-0.31) | 2884  (1644-3828) | 96.87  (95.57-97.79) |
| VoD | 1375  (508-2097) | 11.91  (7.01-14.75) | 0.21  (0.12-0.27) | 3238  (1779-4183) | 97.19  (95.69-98.06) |
| VoA | 4600  (1879-6771) | 40.44  (18.37-52.87) | 0.06  (0.02-0.10) | 45656  (12955-73294) | 91.63  (85.35-95.59) |
| VoA(H) | 4581  (1868-6732) | 40.28  (18.19-52.85) | 0.21  (0.12-0.28) | 11585  (5428-15639) | 87.17  (79.56-92.28) |
| VaR | 4582  (1868-6732) | 40.28  (18.20-52.85) | 0.21  (0.12-0.27) | 11829  (5854-15811) | 87.43  (79.75-92.64) |

**Table A39 Summary of population health impact for various vaccination programmes from 2024 to 2034, with a +50% change in the rate of seeking treatment due to symptoms.** We report posterior means and accompanying 95% credible intervals. Estimates presented were relative to the baseline scenario of no intervention, with transmission probabilities from 2024 to 2034 kept stable at 2018 levels.

**Sensitivity analysis on varying the initial prevalence of asymptomatics in the low-risk group (**$A_{L}\left( t0 \right)/N_{L}$) **by** $\boldsymbol{\pm}$**25 and 50% in value on the prospective impact of each vaccination strategy from 2024 – 2034**

| **Strategy** | **Averted cases**  **(total)** | **Averted cases**  **(%)** | **Averted cases**  **per dose** | **Number of vaccinations administered** | **Primary**  **uptake ratio** |
| --- | --- | --- | --- | --- | --- |
| VbE | 1648  (942-2120) | 14.18  (9.11-17.08) | 0.005  (0.003-0.006) | 172701  (172701-172701) | 100.00  (100.00-100.00) |
| VoD(H) | 1427  (551-2176) | 12.04  (7.12-15.00) | 0.24  (0.14-0.31) | 2954  (1674-3912) | 96.79  (95.47-97.69) |
| VoD | 1427  (551-2176) | 12.04  (7.12-15.00) | 0.22  (0.12-0.28) | 3317  (1853-4252) | 97.13  (95.63-97.96) |
| VoA | 4704  (1948-6947) | 40.26  (18.32-52.57) | 0.06  (0.02-0.10) | 45699  (13004-73327) | 91.62  (85.35-95.57) |
| VoA(H) | 4685  (1938-6922) | 40.10  (18.14-52.55) | 0.22  (0.12-0.28) | 11622  (5502-15669) | 87.13  (79.54-92.24) |
| VaR | 4685  (1939-6922) | 40.10  (18.14-52.55) | 0.21  (0.12-0.27) | 11874  (5906-15851) | 87.40  (79.73-92.61) |

**Table A40 Summary of population health impact for various vaccination programmes from 2024 to 2034, with a -50% change in the initial prevalence of asymptomatic infection in the low risk group.** We report posterior means and accompanying 95% credible intervals. Estimates presented were relative to the baseline scenario of no intervention, with transmission probabilities from 2024 to 2034 kept stable at 2018 levels.

| **Strategy** | **Averted cases**  **(total)** | **Averted cases**  **(%)** | **Averted cases**  **per dose** | **Number of vaccinations administered** | **Primary**  **uptake ratio** |
| --- | --- | --- | --- | --- | --- |
| VbE | 1648  (942-2120) | 14.18  (9.11-17.08) | 0.005  (0.003-0.006) | 172701  (172701-172701) | 100.00  (100.00-100.00) |
| VoD(H) | 1427  (551-2176) | 12.04  (7.12-15.00) | 0.24  (0.14-0.31) | 2954  (1674-3912) | 96.79  (95.47-97.69) |
| VoD | 1427  (551-2176) | 12.04  (7.12-15.00) | 0.22  (0.12-0.28) | 3317  (1853-4252) | 97.13  (95.63-97.96) |
| VoA | 4704  (1948-6947) | 40.26  (18.32-52.57) | 0.06  (0.02-0.10) | 45699  (13004-73327) | 91.62  (85.35-95.57) |
| VoA(H) | 4685  (1938-6922) | 40.10  (18.14-52.55) | 0.22  (0.12-0.28) | 11622  (5502-15669) | 87.13  (79.54-92.24) |
| VaR | 4685  (1939-6922) | 40.10  (18.14-52.55) | 0.21  (0.12-0.27) | 11874  (5906-15851) | 87.40  (79.73-92.61) |

**Table A41 Summary of population health impact for various vaccination programmes from 2024 to 2034, with a -25% change in the initial prevalence of asymptomatic infection in the low risk group.** We report posterior means and accompanying 95% credible intervals. Estimates presented were relative to the baseline scenario of no intervention, with transmission probabilities from 2024 to 2034 kept stable at 2018 levels.

| **Strategy** | **Averted cases**  **(total)** | **Averted cases**  **(%)** | **Averted cases**  **per dose** | **Number of vaccinations administered** | **Primary**  **uptake ratio** |
| --- | --- | --- | --- | --- | --- |
| VbE | 1648  (942-2120) | 14.18  (9.11-17.08) | 0.005  (0.003-0.006) | 172701  (172701-172701) | 100.00  (100.00-100.00) |
| VoD(H) | 1427  (551-2176) | 12.04  (7.12-15.00) | 0.24  (0.14-0.31) | 2954  (1674-3912) | 96.79  (95.47-97.69) |
| VoD | 1427  (551-2176) | 12.04  (7.12-15.00) | 0.22  (0.12-0.28) | 3317  (1853-4252) | 97.13  (95.63-97.96) |
| VoA | 4704  (1948-6947) | 40.26  (18.32-52.57) | 0.06  (0.02-0.10) | 45699  (13004-73327) | 91.62  (85.35-95.57) |
| VoA(H) | 4685  (1938-6922) | 40.10  (18.14-52.55) | 0.22  (0.12-0.28) | 11622  (5502-15669) | 87.13  (79.54-92.24) |
| VaR | 4685  (1939-6922) | 40.10  (18.14-52.55) | 0.21  (0.12-0.27) | 11874  (5906-15851) | 87.40  (79.73-92.61) |

**Table A42 Summary of population health impact for various vaccination programmes from 2024 to 2034, with a +25% change in the initial prevalence of asymptomatic infection in the low risk group.** We report posterior means and accompanying 95% credible intervals. Estimates presented were relative to the baseline scenario of no intervention, with transmission probabilities from 2024 to 2034 kept stable at 2018 levels.

| **Strategy** | **Averted cases**  **(total)** | **Averted cases**  **(%)** | **Averted cases**  **per dose** | **Number of vaccinations administered** | **Primary**  **uptake ratio** |
| --- | --- | --- | --- | --- | --- |
| VbE | 1648  (942-2120) | 14.18  (9.11-17.08) | 0.005  (0.003-0.006) | 172701  (172701-172701) | 100.00  (100.00-100.00) |
| VoD(H) | 1427  (551-2176) | 12.04  (7.12-15.00) | 0.24  (0.14-0.31) | 2954  (1674-3912) | 96.79  (95.47-97.69) |
| VoD | 1427  (551-2176) | 12.04  (7.12-15.00) | 0.22  (0.12-0.28) | 3317  (1853-4252) | 97.13  (95.63-97.96) |
| VoA | 4704  (1948-6947) | 40.26  (18.32-52.57) | 0.06  (0.02-0.10) | 45699  (13004-73327) | 91.62  (85.35-95.57) |
| VoA(H) | 4685  (1938-6922) | 40.10  (18.14-52.55) | 0.22  (0.12-0.28) | 11622  (5502-15669) | 87.13  (79.54-92.24) |
| VaR | 4685  (1939-6922) | 40.10  (18.14-52.55) | 0.21  (0.12-0.27) | 11874  (5906-15851) | 87.40  (79.73-92.61) |

**Table A43 Summary of population health impact for various vaccination programmes from 2024 to 2034, with a +50% change in the initial prevalence of asymptomatic infection in the low risk group.** We report posterior means and accompanying 95% credible intervals. Estimates presented were relative to the baseline scenario of no intervention, with transmission probabilities from 2024 to 20˙34 kept stable at 2018 levels.

**Sensitivity analysis on varying the initial prevalence of asymptomatics in the high-risk group (**$A_{H}\left( t0 \right)/N_{H}$) **by** $\boldsymbol{\pm}$**25 and 50% in value on the prospective impact of each vaccination strategy from 2024 – 2034**

| **Strategy** | **Averted cases**  **(total)** | **Averted cases**  **(%)** | **Averted cases**  **per dose** | **Number of vaccinations administered** | **Primary**  **uptake ratio** |
| --- | --- | --- | --- | --- | --- |
| VbE | 1647  (942-2120) | 14.18  (9.11-17.09) | 0.005  (0.003-0.006) | 172701  (172701-172701) | 100.00  (100.00-100.00) |
| VoD(H) | 1425  (550-2176) | 12.03  (7.12-14.99) | 0.24  (0.14-0.31) | 2952  (1671-3906) | 96.80  (95.47-97.69) |
| VoD | 1426  (550-2176) | 12.04  (7.12-14.99) | 0.22  (0.12-0.28) | 3315  (1852-4247) | 97.13  (95.63-97.96) |
| VoA | 4702  (1948-6941) | 40.27  (18.32-52.58) | 0.06  (0.02-0.10) | 45698  (13003-73326) | 91.62  (85.35-95.57) |
| VoA(H) | 4683  (1938-6920) | 40.11  (18.14-52.55) | 0.22  (0.12-0.28) | 11621  (5499-15663) | 87.13  (79.54-92.24) |
| VaR | 4683  (1938-6921) | 40.11  (18.14-52.55) | 0.21  (0.12-0.27) | 11873  (5903-15849) | 87.40  (79.73-92.61) |

**Table A44 Summary of population health impact for various vaccination programmes from 2024 to 2034, with a -50% change in the initial prevalence of asymptomatic infection in the high risk group.** We report posterior means and accompanying 95% credible intervals. Estimates presented were relative to the baseline scenario of no intervention, with transmission probabilities from 2024 to 2034 kept stable at 2018 levels.

| **Strategy** | **Averted cases**  **(total)** | **Averted cases**  **(%)** | **Averted cases**  **per dose** | **Number of vaccinations administered** | **Primary**  **uptake ratio** |
| --- | --- | --- | --- | --- | --- |
| VbE | 1647  (942-2120) | 14.18  (9.11-17.08) | 0.005  (0.003-0.006) | 172701  (172701-172701) | 100.00  (100.00-100.00) |
| VoD(H) | 1426  (551-2176) | 12.04  (7.12-14.99) | 0.24  (0.14-0.31) | 2953  (1673-3910) | 96.79  (95.47-97.69) |
| VoD | 1427  (551-2176) | 12.04  (7.12-14.99) | 0.22  (0.12-0.28) | 3317  (1852-4250) | 97.13  (95.63-97.96) |
| VoA | 4703  (1948-6945) | 40.26  (18.32-52.57) | 0.06  (0.02-0.10) | 45699  (13004-73327) | 91.62  (85.35-95.57) |
| VoA(H) | 4684  (1938-6922) | 40.10  (18.14-52.55) | 0.22  (0.12-0.28) | 11622  (5501-15667) | 87.13  (79.54-92.24) |
| VaR | 4684  (1938-6922) | 40.10  (18.14-52.55) | 0.21  (0.12-0.27) | 11874  (5905-15851) | 87.40  (79.73-92.61) |

**Table A45 Summary of population health impact for various vaccination programmes from 2024 to 2034, with a -25% change in initial prevalence of asymptomatic infection in the high risk group.** We report posterior means and accompanying 95% credible intervals. Estimates presented were relative to the baseline scenario of no intervention, with transmission probabilities from 2024 to 2034 kept stable at 2018 levels.

| **Strategy** | **Averted cases**  **(total)** | **Averted cases**  **(%)** | **Averted cases**  **per dose** | **Number of vaccinations administered** | **Primary**  **uptake ratio** |
| --- | --- | --- | --- | --- | --- |
| VbE | 1648  (942-2120) | 14.17  (9.11-17.07) | 0.005  (0.003-0.006) | 172701  (172701-172701) | 100.00  (100.00-100.00) |
| VoD(H) | 1427  (551-2177) | 12.04  (7.12-15.00) | 0.24  (0.14-0.31) | 2954  (1674-3913) | 96.79  (95.47-97.69) |
| VoD | 1428  (551-2177) | 12.04  (7.12-15.00) | 0.22  (0.12-0.28) | 3318  (1853-4253) | 97.13  (95.63-97.96) |
| VoA | 4704  (1948-6948) | 40.26  (18.32-52.57) | 0.06  (0.02-0.10) | 45700  (13004-73328) | 91.62  (85.35-95.57) |
| VoA(H) | 4685  (1939-6922) | 40.09  (18.14-52.55) | 0.22  (0.12-0.28) | 11623  (5502-15671) | 87.13  (79.54-92.24) |
| VaR | 4685  (1939-6923) | 40.09  (18.14-52.55) | 0.21  (0.12-0.27) | 11874  (5907-15852) | 87.40  (79.73-92.61) |

**Table A46 Summary of population health impact for various vaccination programmes from 2024 to 2034, with a +25% change in the initial prevalence of asymptomatic infection in the high risk group.** We report posterior means and accompanying 95% credible intervals. Estimates presented were relative to the baseline scenario of no intervention, with transmission probabilities from 2024 to 2034 kept stable at 2018 levels.

| **Strategy** | **Averted cases**  **(total)** | **Averted cases**  **(%)** | **Averted cases**  **per dose** | **Number of vaccinations administered** | **Primary**  **uptake ratio** |
| --- | --- | --- | --- | --- | --- |
| VbE | 1648  (942-2120) | 14.17  (9.11-17.07) | 0.005  (0.003-0.006) | 172701  (172701-172701) | 100.00  (100.00-100.00) |
| VoD(H) | 1428  (551-2177) | 12.04  (7.12-15.00) | 0.24  (0.14-0.31) | 2955  (1674-3914) | 96.79  (95.47-97.69) |
| VoD | 1428  (551-2177) | 12.04  (7.12-15.00) | 0.22  (0.12-0.28) | 3318  (1854-4253) | 97.13  (95.63-97.96) |
| VoA | 4705  (1948-6948) | 40.25  (18.32-52.57) | 0.06  (0.02-0.10) | 45700  (13004-73328) | 91.62  (85.35-95.57) |
| VoA(H) | 4685  (1939-6923) | 40.09  (18.14-52.55) | 0.22  (0.12-0.28) | 11623  (5502-15672) | 87.13  (79.54-92.24) |
| VaR | 4686  (1939-6923) | 40.09  (18.14-52.55) | 0.21  (0.12-0.27) | 11875  (5907-15852) | 87.40  (79.73-92.61) |

**Table A47 Summary of population health impact for various vaccination programmes from 2024 to 2034, with a +50% change in the initial prevalence of asymptomatic infection in the high risk group.** We report posterior means and accompanying 95% credible intervals. Estimates presented were relative to the baseline scenario of no intervention, with transmission probabilities from 2024 to 2034 kept stable at 2018 levels.

**Sensitivity analysis on varying the asymptomatic rate (**$\psi)$ **by** $\boldsymbol{\pm}$**25 and 50% in value on the prospective impact of each vaccination strategy from 2024 – 2034**

| **Strategy** | **Averted cases**  **(total)** | **Averted cases**  **(%)** | **Averted cases**  **per dose** | **Number of vaccinations administered** | **Primary**  **uptake ratio** |
| --- | --- | --- | --- | --- | --- |
| VbE | 1249  (705-1625) | 8.50  (6.30-10.06) | 0.004  (0.002-0.005) | 172701  (172701-172701) | 100.00  (100.00-100.00) |
| VoD(H) | 1352  (547-2257) | 8.97  (5.47-11.22) | 0.18  (0.10-0.23) | 3828  (2405-5124) | 95.83  (94.05-97.00) |
| VoD | 1353  (547-2257) | 8.97  (5.48-11.23) | 0.16  (0.09-0.20) | 4307  (2807-5708) | 96.27  (94.36-97.37) |
| VoA | 4137  (1387-6499) | 27.83  (11.84-39.24) | 0.05  (0.02-0.08) | 46126  (13883-73512) | 91.46  (85.30-95.37) |
| VoA(H) | 4108  (1372-6457) | 27.63  (11.69-39.02) | 0.18  (0.10-0.24) | 11977  (6076-15965) | 86.72  (79.42-91.95) |
| VaR | 4108  (1372-6458) | 27.64  (11.70-39.03) | 0.18  (0.10-0.23) | 12362  (6468-16417) | 87.13  (79.68-92.37) |

**Table A48 Summary of population health impact for various vaccination programmes from 2024 to 2034, with a -50% change in the number of asymptomatics per incident infection.** We report posterior means and accompanying 95% credible intervals. Estimates presented were relative to the baseline scenario of no intervention, with transmission probabilities from 2024 to 2034 kept stable at 2018 levels.

| **Strategy** | **Averted cases**  **(total)** | **Averted cases**  **(%)** | **Averted cases**  **per dose** | **Number of vaccinations administered** | **Primary**  **uptake ratio** |
| --- | --- | --- | --- | --- | --- |
| VbE | 1593  (917-2100) | 10.68  (7.37-12.61) | 0.005  (0.003-0.006) | 172701  (172701-172701) | 100.00  (100.00-100.00) |
| VoD(H) | 1725  (695-2811) | 11.31  (6.98-14.30) | 0.23  (0.13-0.31) | 3774  (2445-4839) | 95.89  (94.28-96.97) |
| VoD | 1726  (695-2812) | 11.32  (6.98-14.31) | 0.20  (0.12-0.27) | 4249  (2774-5485) | 96.33  (94.54-97.39) |
| VoA | 5106  (1863-7800) | 34.01  (15.05-46.01) | 0.06  (0.03-0.10) | 46199  (13928-73587) | 91.43  (85.30-95.21) |
| VoA(H) | 5078  (1852-7744) | 33.83  (14.97-45.76) | 0.23  (0.13-0.30) | 12049  (6269-16047) | 86.64  (79.37-91.82) |
| VaR | 5078  (1852-7744) | 33.83  (14.97-45.77) | 0.22  (0.13-0.29) | 12407  (6795-16357) | 87.02  (79.62-92.15) |

**Table A49 Summary of population health impact for various vaccination programmes from 2024 to 2034, with a -25% change in the number of asymptomatics per incident infection.** We report posterior means and accompanying 95% credible intervals. Estimates presented were relative to the baseline scenario of no intervention, with transmission probabilities from 2024 to 2034 kept stable at 2018 levels.

| **Strategy** | **Averted cases**  **(total)** | **Averted cases**  **(%)** | **Averted cases**  **per dose** | **Number of vaccinations administered** | **Primary**  **uptake ratio** |
| --- | --- | --- | --- | --- | --- |
| VbE | 917  (61-1640) | 18.75  (11.16-22.35) | 0.003  (0.000-0.005) | 172701  (172701-172701) | 100.00  (100.00-100.00) |
| VoD(H) | 447  (3-1259) | 7.65  (0.75-12.19) | 0.14  (0.01-0.24) | 1353  (102-2685) | 98.54  (96.15-99.68) |
| VoD | 447  (3-1259) | 7.65  (0.75-12.19) | 0.13  (0.01-0.22) | 1512  (117-2963) | 98.68  (96.50-99.72) |
| VoA | 2231  (130-4651) | 44.05  (18.80-56.70) | 0.03  (0.00-0.06) | 44808  (11875-72591) | 91.94  (85.44-96.10) |
| VoA(H) | 2223  (129-4636) | 43.90  (18.73-56.61) | 0.11  (0.01-0.21) | 10848  (4077-15234) | 88.00  (79.87-93.60) |
| VaR | 2223  (129-4636) | 43.90  (18.73-56.61) | 0.11  (0.01-0.20) | 10948  (4225-15263) | 88.11  (79.97-93.66) |

**Table A50 Summary of population health impact for various vaccination programmes from 2024 to 2034, with a +25% change in the number of asymptomatics per incident infection.** We report posterior means and accompanying 95% credible intervals. Estimates presented were relative to the baseline scenario of no intervention, with transmission probabilities from 2024 to 2034 kept stable at 2018 levels.

| **Strategy** | **Averted cases**  **(total)** | **Averted cases**  **(%)** | **Averted cases**  **per dose** | **Number of vaccinations administered** | **Primary**  **uptake ratio** |
| --- | --- | --- | --- | --- | --- |
| VbE | 170  (0-912) | 16.53  (7.57-21.94) | 0.000  (0.000-0.003) | 172701  (172701-172701) | 100.00  (100.00-100.00) |
| VoD(H) | 45  (0-364) | 1.68  (0.00-7.63) | 0.03  (0.00-0.14) | 259  (0-1319) | 99.72  (97.15-100.00) |
| VoD | 45  (0-364) | 1.68  (0.00-7.63) | 0.03  (0.00-0.13) | 288  (0-1439) | 99.75  (97.35-100.00) |
| VoA | 382  (0-2140) | 36.48  (11.24-53.26) | 0.00  (0.00-0.03) | 44285  (10734-72153) | 92.12  (85.51-96.37) |
| VoA(H) | 381  (0-2139) | 36.34  (11.24-53.08) | 0.02  (0.00-0.11) | 10389  (3183-14872) | 88.51  (80.22-94.07) |
| VaR | 381  (0-2139) | 36.34  (11.24-53.08) | 0.02  (0.00-0.11) | 10407  (3201-14872) | 88.53  (80.25-94.22) |

**Table A51 Summary of population health impact for various vaccination programmes from 2024 to 2034, with a +50% change in the number of asymptomatics per incident infection.** We report posterior means and accompanying 95% credible intervals. Estimates presented were relative to the baseline scenario of no intervention, with transmission probabilities from 2024 to 2034 kept stable at 2018 levels.

**Sensitivity analysis on varying the partner change rate for the low risk group (**$c_{L})$ **by** $\boldsymbol{\pm}$**25 and 50% in value on the prospective impact of each vaccination strategy from 2024 – 2034**

| **Strategy** | **Averted cases**  **(total)** | **Averted cases**  **(%)** | **Averted cases**  **per dose** | **Number of vaccinations administered** | **Primary**  **uptake ratio** |
| --- | --- | --- | --- | --- | --- |
| VbE | 1564  (868-2078) | 13.59  (9.27-16.47) | 0.005  (0.003-0.006) | 172701  (172701-172701) | 100.00  (100.00-100.00) |
| VoD(H) | 1433  (522-2243) | 12.22  (7.19-15.91) | 0.23  (0.13-0.31) | 3099  (1867-3885) | 96.64  (95.32-97.63) |
| VoD | 1434  (522-2243) | 12.22  (7.19-15.91) | 0.22  (0.12-0.30) | 3262  (2009-4059) | 96.80  (95.43-97.76) |
| VoA | 4459  (1725-6665) | 38.55  (17.05-51.47) | 0.06  (0.02-0.10) | 44300  (10341-71584) | 91.82  (86.06-95.58) |
| VoA(H) | 4452  (1720-6649) | 38.48  (17.03-51.38) | 0.21  (0.11-0.28) | 11420  (4680-15676) | 87.36  (80.96-92.57) |
| VaR | 4452  (1720-6649) | 38.48  (17.03-51.38) | 0.21  (0.11-0.27) | 11537  (4891-15805) | 87.48  (81.06-92.69) |

**Table A52 Summary of population health impact for various vaccination programmes from 2024 to 2034, with a -50% change in the partner change rate for the low risk group.** We report posterior means and accompanying 95% credible intervals. Estimates presented were relative to the baseline scenario of no intervention, with transmission probabilities from 2024 to 2034 kept stable at 2018 levels.

| **Strategy** | **Averted cases**  **(total)** | **Averted cases**  **(%)** | **Averted cases**  **per dose** | **Number of vaccinations administered** | **Primary**  **uptake ratio** |
| --- | --- | --- | --- | --- | --- |
| VbE | 1594  (850-2105) | 13.72  (8.78-16.81) | 0.005  (0.002-0.006) | 172701  (172701-172701) | 100.00  (100.00-100.00) |
| VoD(H) | 1429  (451-2230) | 12.05  (7.05-15.26) | 0.24  (0.13-0.31) | 3043  (1563-3916) | 96.70  (95.13-97.64) |
| VoD | 1429  (451-2231) | 12.05  (7.05-15.26) | 0.22  (0.12-0.29) | 3307  (1754-4323) | 96.95  (95.45-97.86) |
| VoA | 4574  (1994-6784) | 39.15  (19.78-50.80) | 0.05  (0.02-0.09) | 46346  (13989-78220) | 91.59  (85.06-95.08) |
| VoA(H) | 4561  (1983-6766) | 39.04  (19.68-50.69) | 0.21  (0.11-0.28) | 11534  (5977-15607) | 87.23  (81.14-91.65) |
| VaR | 4561  (1983-6766) | 39.04  (19.69-50.69) | 0.21  (0.11-0.27) | 11720  (6374-15859) | 87.43  (81.29-91.93) |

**Table A53 Summary of population health impact for various vaccination programmes from 2024 to 2034, with a -25% change in the partner change rate for the low risk group.** We report posterior means and accompanying 95% credible intervals. Estimates presented were relative to the baseline scenario of no intervention, with transmission probabilities from 2024 to 2034 kept stable at 2018 levels.

| **Strategy** | **Averted cases**  **(total)** | **Averted cases**  **(%)** | **Averted cases**  **per dose** | **Number of vaccinations administered** | **Primary**  **uptake ratio** |
| --- | --- | --- | --- | --- | --- |
| VbE | 1556  (788-2118) | 13.47  (8.82-16.76) | 0.005  (0.002-0.006) | 172701  (172701-172701) | 100.00  (100.00-100.00) |
| VoD(H) | 1314  (374-2047) | 11.13  (5.90-14.39) | 0.23  (0.12-0.31) | 2893  (1591-3858) | 96.86  (95.23-97.82) |
| VoD | 1315  (374-2048) | 11.14  (5.90-14.39) | 0.20  (0.10-0.26) | 3334  (1873-4381) | 97.25  (95.36-98.08) |
| VoA | 4468  (1675-6634) | 38.44  (19.36-50.46) | 0.05  (0.02-0.09) | 47296  (13573-77785) | 91.44  (85.15-95.25) |
| VoA(H) | 4441  (1666-6618) | 38.21  (19.17-50.16) | 0.20  (0.10-0.27) | 11664  (5055-15768) | 87.08  (80.87-91.96) |
| VaR | 4442  (1666-6618) | 38.22  (19.18-50.16) | 0.20  (0.10-0.26) | 11975  (5513-15934) | 87.41  (81.15-92.26) |

**Table A54 Summary of population health impact for various vaccination programmes from 2024 to 2034, with a +25% change in the partner change rate for the low risk group.** We report posterior means and accompanying 95% credible intervals. Estimates presented were relative to the baseline scenario of no intervention, with transmission probabilities from 2024 to 2034 kept stable at 2018 levels.

| **Strategy** | **Averted cases**  **(total)** | **Averted cases**  **(%)** | **Averted cases**  **per dose** | **Number of vaccinations administered** | **Primary**  **uptake ratio** |
| --- | --- | --- | --- | --- | --- |
| VbE | 1680  (1004-2266) | 14.08  (9.63-17.47) | 0.005  (0.003-0.007) | 172701  (172701-172701) | 100.00  (100.00-100.00) |
| VoD(H) | 1401  (548-2140) | 11.53  (7.39-14.88) | 0.25  (0.15-0.33) | 2873  (1623-3770) | 96.88  (95.29-97.80) |
| VoD | 1402  (548-2141) | 11.54  (7.40-14.88) | 0.20  (0.12-0.28) | 3431  (1942-4401) | 97.36  (95.73-98.18) |
| VoA | 4633  (1926-6819) | 38.60  (18.53-51.42) | 0.06  (0.03-0.10) | 44035  (12515-74755) | 91.89  (85.36-95.75) |
| VoA(H) | 4598  (1917-6772) | 38.31  (18.30-51.16) | 0.22  (0.12-0.29) | 11264  (4875-15447) | 87.53  (81.77-92.81) |
| VaR | 4599  (1918-6773) | 38.32  (18.31-51.17) | 0.21  (0.12-0.27) | 11660  (5511-15773) | 87.95  (82.12-93.23) |

**Table A55 Summary of population health impact for various vaccination programmes from 2024 to 2034, with a +50% change in the partner change rate for the low risk group.** We report posterior means and accompanying 95% credible intervals. Estimates presented were relative to the baseline scenario of no intervention, with transmission probabilities from 2024 to 2034 kept stable at 2018 levels.

**Sensitivity analysis on varying the partner change rate for the high risk group (**$c_{h})$ **by** $\boldsymbol{\pm}$**25 and 50% in value on the prospective impact of each vaccination strategy from 2024 – 2034**

| **Strategy** | **Averted cases**  **(total)** | **Averted cases**  **(%)** | **Averted cases**  **per dose** | **Number of vaccinations administered** | **Primary**  **uptake ratio** |
| --- | --- | --- | --- | --- | --- |
| VbE | 1706  (1345-1960) | 14.63  (11.21-16.60) | 0.005  (0.004-0.006) | 172701  (172701-172701) | 100.00  (100.00-100.00) |
| VoD(H) | 1592  (945-2060) | 13.54  (10.84-15.47) | 0.25  (0.20-0.29) | 3173  (2271-3767) | 96.56  (95.63-97.20) |
| VoD | 1592  (945-2060) | 13.54  (10.84-15.47) | 0.25  (0.19-0.29) | 3258  (2302-3850) | 96.65  (95.72-97.27) |
| VoA | 5641  (3124-7177) | 48.25  (27.63-58.40) | 0.07  (0.04-0.13) | 48046  (13980-80971) | 90.33  (83.75-93.81) |
| VoA(H) | 5638  (3122-7174) | 48.22  (27.59-58.40) | 0.22  (0.15-0.26) | 14090  (7223-18309) | 84.34  (78.05-89.29) |
| VaR | 5638  (3122-7174) | 48.22  (27.59-58.40) | 0.22  (0.15-0.25) | 14143  (7321-18327) | 84.40  (78.06-89.41) |

**Table A56 Summary of population health impact for various vaccination programmes from 2024 to 2034, with a -50% change in the partner change rate for the high risk group.** We report posterior means and accompanying 95% credible intervals. Estimates presented were relative to the baseline scenario of no intervention, with transmission probabilities from 2024 to 2034 kept stable at 2018 levels.

| **Strategy** | **Averted cases**  **(total)** | **Averted cases**  **(%)** | **Averted cases**  **per dose** | **Number of vaccinations administered** | **Primary**  **uptake ratio** |
| --- | --- | --- | --- | --- | --- |
| VbE | 1991  (1243-2562) | 14.64  (10.32-17.50) | 0.006  (0.004-0.007) | 172701  (172701-172701) | 100.00  (100.00-100.00) |
| VoD(H) | 1821  (804-2854) | 13.17  (8.74-16.40) | 0.29  (0.18-0.38) | 3149  (2132-3973) | 96.58  (94.96-97.41) |
| VoD | 1823  (805-2858) | 13.18  (8.75-16.40) | 0.24  (0.15-0.31) | 3831  (2583-4875) | 97.16  (95.58-97.90) |
| VoA | 5876  (2426-8461) | 42.96  (19.71-54.57) | 0.08  (0.03-0.15) | 43695  (12050-73717) | 91.39  (84.32-94.86) |
| VoA(H) | 5837  (2407-8413) | 42.68  (19.61-54.29) | 0.25  (0.16-0.32) | 12457  (5599-16520) | 86.19  (79.18-91.09) |
| VaR | 5838  (2408-8413) | 42.69  (19.62-54.29) | 0.24  (0.15-0.31) | 12916  (6221-16938) | 86.67  (79.57-91.74) |

**Table A57 Summary of population health impact for various vaccination programmes from 2024 to 2034, with a -25% change in the partner change rate for the high risk group.** We report posterior means and accompanying 95% credible intervals. Estimates presented were relative to the baseline scenario of no intervention, with transmission probabilities from 2024 to 2034 kept stable at 2018 levels.

| **Strategy** | **Averted cases**  **(total)** | **Averted cases**  **(%)** | **Averted cases**  **per dose** | **Number of vaccinations administered** | **Primary**  **uptake ratio** |
| --- | --- | --- | --- | --- | --- |
| VbE | 1659  (1136-2031) | 14.31  (10.75-16.79) | 0.005  (0.003-0.006) | 172701  (172701-172701) | 100.00  (100.00-100.00) |
| VoD(H) | 1453  (655-2166) | 12.31  (8.85-14.82) | 0.24  (0.17-0.30) | 2988  (1846-3863) | 96.76  (95.37-97.61) |
| VoD | 1454  (655-2166) | 12.32  (8.85-14.82) | 0.22  (0.15-0.28) | 3301  (2059-4279) | 97.05  (95.59-97.85) |
| VoA | 4763  (2103-6885) | 40.80  (20.86-54.58) | 0.07  (0.03-0.12) | 40542  (12940-68963) | 92.07  (86.88-95.40) |
| VoA(H) | 4749  (2095-6848) | 40.68  (20.82-54.58) | 0.22  (0.15-0.27) | 11592  (5363-16081) | 87.16  (80.44-92.26) |
| VaR | 4749  (2095-6848) | 40.68  (20.82-54.58) | 0.21  (0.15-0.26) | 11807  (5615-16233) | 87.39  (80.69-92.52) |

**Table A58 Summary of population health impact for various vaccination programmes from 2024 to 2034, with a +25% change in the partner change rate for the high risk group.** We report posterior means and accompanying 95% credible intervals. Estimates presented were relative to the baseline scenario of no intervention, with transmission probabilities from 2024 to 2034 kept stable at 2018 levels.

| **Strategy** | **Averted cases**  **(total)** | **Averted cases**  **(%)** | **Averted cases**  **per dose** | **Number of vaccinations administered** | **Primary**  **uptake ratio** |
| --- | --- | --- | --- | --- | --- |
| VbE | 1667  (1002-2128) | 14.42  (10.69-17.02) | 0.005  (0.003-0.006) | 172701  (172701-172701) | 100.00  (100.00-100.00) |
| VoD(H) | 1487  (472-2282) | 12.59  (8.45-15.44) | 0.25  (0.16-0.32) | 3026  (1549-3949) | 96.71  (95.24-97.67) |
| VoD | 1487  (472-2282) | 12.59  (8.45-15.44) | 0.23  (0.14-0.29) | 3282  (1658-4235) | 96.96  (95.38-97.90) |
| VoA | 4638  (1638-6681) | 39.79  (14.87-51.68) | 0.07  (0.02-0.15) | 37545  (6281-65997) | 92.39  (85.68-95.72) |
| VoA(H) | 4628  (1638-6673) | 39.71  (14.86-51.64) | 0.22  (0.12-0.28) | 11111  (3733-15251) | 87.70  (81.03-92.62) |
| VaR | 4628  (1638-6673) | 39.71  (14.86-51.64) | 0.22  (0.12-0.28) | 11292  (3884-15407) | 87.90  (81.17-92.93) |

**Table A59 Summary of population health impact for various vaccination programmes from 2024 to 2034, with a +50% change in the partner change rate for the high risk group.** We report posterior means and accompanying 95% credible intervals. Estimates presented were relative to the baseline scenario of no intervention, with transmission probabilities from 2024 to 2034 kept stable at 2018 levels.

**Sensitivity analysis on varying the proportion of individuals in the high risk group (**$q_{h})$ **to 0.1, 0.2 and 0.3 in value on the prospective impact of each vaccination strategy from 2024 – 2034**

| **Strategy** | **Averted cases**  **(total)** | **Averted cases**  **(%)** | **Averted cases**  **per dose** | **Number of vaccinations administered** | **Primary**  **uptake ratio** |
| --- | --- | --- | --- | --- | --- |
| VbE | 1390  (666-1868) | 12.65  (7.56-16.03) | 0.004  (0.002-0.005) | 172701  (172701-172701) | 100.00  (100.00-100.00) |
| VoD(H) | 1535  (427-2398) | 13.64  (8.08-17.27) | 0.30  (0.16-0.41) | 2549  (1301-3458) | 95.84  (93.42-97.08) |
| VoD | 1535  (427-2399) | 13.65  (8.08-17.28) | 0.25  (0.14-0.33) | 3066  (1438-4024) | 96.50  (93.84-97.68) |
| VoA | 4437  (1830-6497) | 40.12  (19.36-52.67) | 0.05  (0.02-0.10) | 48233  (10417-78854) | 91.29  (84.67-95.00) |
| VoA(H) | 4407  (1820-6459) | 39.85  (19.20-52.38) | 0.28  (0.13-0.37) | 8616  (3918-11252) | 85.66  (79.36-90.50) |
| VaR | 4407  (1821-6460) | 39.85  (19.21-52.38) | 0.26  (0.13-0.34) | 8980  (4653-11530) | 86.23  (80.11-91.32) |

**Table A60 Summary of population health impact for various vaccination programmes from 2024 to 2034, with the proportion of individuals in the high risk group changed to 0.1.** We report posterior means and accompanying 95% credible intervals. Estimates presented were relative to the baseline scenario of no intervention, with transmission probabilities from 2024 to 2034 kept stable at 2018 levels.

| **Strategy** | **Averted cases**  **(total)** | **Averted cases**  **(%)** | **Averted cases**  **per dose** | **Number of vaccinations administered** | **Primary**  **uptake ratio** |
| --- | --- | --- | --- | --- | --- |
| VbE | 1847  (1133-2311) | 15.00  (10.86-17.59) | 0.005  (0.003-0.007) | 172701  (172701-172701) | 100.00  (100.00-100.00) |
| VoD(H) | 1345  (620-2058) | 10.75  (7.01-13.20) | 0.21  (0.13-0.26) | 3286  (2175-4199) | 97.33  (96.05-98.00) |
| VoD | 1346  (620-2059) | 10.75  (7.01-13.20) | 0.19  (0.12-0.24) | 3572  (2343-4503) | 97.53  (96.24-98.19) |
| VoA | 5104  (2085-7435) | 41.20  (19.83-53.53) | 0.06  (0.03-0.10) | 47314  (14913-74539) | 91.24  (85.42-95.01) |
| VoA(H) | 5089  (2077-7429) | 41.08  (19.81-53.50) | 0.18  (0.11-0.23) | 15214  (7096-20709) | 87.37  (81.39-92.45) |
| VaR | 5089  (2077-7429) | 41.08  (19.81-53.50) | 0.18  (0.11-0.22) | 15406  (7264-20812) | 87.52  (81.58-92.61) |

**Table A61 Summary of population health impact for various vaccination programmes from 2024 to 2034, with the proportion of individuals in the high risk group changed to 0.2.** We report posterior means and accompanying 95% credible intervals. Estimates presented were relative to the baseline scenario of no intervention, with transmission probabilities from 2024 to 2034 kept stable at 2018 levels.

| **Strategy** | **Averted cases**  **(total)** | **Averted cases**  **(%)** | **Averted cases**  **per dose** | **Number of vaccinations administered** | **Primary**  **uptake ratio** |
| --- | --- | --- | --- | --- | --- |
| VbE | 2159  (1391-2765) | 15.39  (11.38-18.32) | 0.006  (0.004-0.008) | 172701  (172701-172701) | 100.00  (100.00-100.00) |
| VoD(H) | 1279  (524-2009) | 8.95  (5.98-11.11) | 0.16  (0.10-0.20) | 3984  (2248-5181) | 97.84  (96.78-98.42) |
| VoD | 1280  (524-2010) | 8.95  (5.98-11.11) | 0.15  (0.10-0.20) | 4172  (2369-5402) | 97.94  (96.81-98.50) |
| VoA | 5046  (1632-7700) | 35.74  (12.91-50.71) | 0.07  (0.03-0.11) | 39002  (8389-67793) | 92.32  (85.82-96.14) |
| VoA(H) | 5038  (1631-7693) | 35.68  (12.90-50.57) | 0.14  (0.08-0.18) | 18821  (5460-28216) | 89.61  (82.98-94.61) |
| VaR | 5039  (1631-7693) | 35.68  (12.90-50.57) | 0.14  (0.08-0.18) | 18956  (5587-28372) | 89.69  (83.08-94.71) |

**Table A62 Summary of population health impact for various vaccination programmes from 2024 to 2034, with the proportion of individuals in the high risk group changed to 0.3.** We report posterior means and accompanying 95% credible intervals. Estimates presented were relative to the baseline scenario of no intervention, with transmission probabilities from 2024 to 2034 kept stable at 2018 levels.

**Variance decomposition on** $\boldsymbol{\beta}_{\boldsymbol{2004}}$ **by keeping the parameter at the posterior median while allowing all other parameters to vary**

**Fig A13 Projected number of annual diagnosed cases and evaluation metrics by vaccination programme over 2024 – 2054 through variance decomposition on** $\boldsymbol{\beta}_{\boldsymbol{2004}}$**.** Posterior means are marked with a square, and the 95% credible interval are indicated by the boundaries of the box. Model was calibrated to case series of male incidence multiplied by the proportion of reported gonorrhea cases in the UK MSM population. Projections assumed that transmission probabilities remained stable in 2024 – 2034 at 2018 levels. 1000 posterior draws were used to generate future projections and calculate programme-specific metrics. $\beta_{2004}$ was kept at median values while all other parameters were allowed to vary

The series of boxplots illustrate the annual future number of diagnosed cases under different vaccination strategies compared to a no-vaccination programme baseline. Each column represented a different vaccination programme. Respectively, these were denoted (**A**) vaccination before entry [VbE], (**B**) VoD strategies by offering vaccinations in these groups to only high sexual activity individuals (VoD[H]), (**C**) vaccination on diagnoses with gonorrhea [VoD], (**D**) vaccination on attendance [VoA], (**E**) VoA strategies by offering vaccinations in these groups to only high sexual activity individuals (VoA[H]) and (**F**) vaccination according to risk [VaR].

The radar graph visualized the impact, efficiency, and uptake of each vaccination programme from 2024 – 2034, 2034 – 2044 and 2044 to 2054 respectively. Metrics were averted cases by percentage (A.P.), averted cases per dose (A.D.), number of vaccinations administered (N.V.), and primary uptake ratio (P.U.). Each sample’s metric value was normalized by the maximum and minimum across strategies for visual clarity, points closer to the center are closer to the overall strategy minimum metric score, and the radar border represents the overall maximum score each strategy can achieve.

**Variance decomposition on** $\boldsymbol{\beta}_{\boldsymbol{2018}}$ **by keeping the parameter at the posterior median value while allowing all other parameters to vary**

**Fig A14 Projected number of annual diagnosed cases and evaluation metrics by vaccination programme over 2024 – 2054 through variance decomposition on** $\boldsymbol{\beta}_{\boldsymbol{20}\boldsymbol{18}}$**.** Posterior means are marked with a square, and the 95% credible interval are indicated by the boundaries of the box. Model was calibrated to case series of male incidence multiplied by the proportion of reported gonorrhea cases in the UK MSM population. Projections assumed that transmission probabilities remained stable in 2024 – 2034 at 2018 levels. 1000 posterior draws were used to generate future projections and calculate programme-specific metrics. $\beta_{2018}$ was kept at median values while all other parameters were allowed to vary

The series of boxplots illustrate the annual future number of diagnosed cases under different vaccination strategies compared to a no-vaccination programme baseline. Each column represented a different vaccination programme. Respectively, these were denoted (**A**) vaccination before entry [VbE], (**B**) VoD strategies by offering vaccinations in these groups to only high sexual activity individuals (VoD[H]), (**C**) vaccination on diagnoses with gonorrhea [VoD], (**D**) vaccination on attendance [VoA], (**E**) VoA strategies by offering vaccinations in these groups to only high sexual activity individuals (VoA[H]) and (**F**) vaccination according to risk [VaR].

The radar graph visualized the impact, efficiency, and uptake of each vaccination programme from 2024 – 2034, 2034 – 2044 and 2044 to 2054 respectively. Metrics were averted cases by percentage (A.P.), averted cases per dose (A.D.), number of vaccinations administered (N.V.), and primary uptake ratio (P.U.). Each sample’s metric value was normalized by the maximum and minimum across strategies for visual clarity, points closer to the center are closer to the overall strategy minimum metric score, and the radar border represents the overall maximum score each strategy can achieve.

**Variance decomposition on** $\boldsymbol{\epsilon}$ **by keeping the parameter at the posterior median while allowing all other parameters to vary**

**Fig A15 Projected number of annual diagnosed cases and evaluation metrics by vaccination programme over 2024 – 2054 through variance decomposition on** $\boldsymbol{\epsilon}$**.** Posterior means are marked with a square, and the 95% credible interval are indicated by the boundaries of the box. Model was calibrated to case series of male incidence multiplied by the proportion of reported gonorrhea cases in the UK MSM population. Projections assumed that transmission probabilities remained stable in 2024 – 2034 at 2018 levels. 1000 posterior draws were used to generate future projections and calculate programme-specific metrics. $\epsilon$ was kept at median values while all other parameters were allowed to vary

The series of boxplots illustrate the annual future number of diagnosed cases under different vaccination strategies compared to a no-vaccination programme baseline. Each column represented a different vaccination programme. Respectively, these were denoted (**A**) vaccination before entry [VbE], (**B**) VoD strategies by offering vaccinations in these groups to only high sexual activity individuals (VoD[H]), (**C**) vaccination on diagnoses with gonorrhea [VoD], (**D**) vaccination on attendance [VoA], (**E**) VoA strategies by offering vaccinations in these groups to only high sexual activity individuals (VoA[H]) and (**F**) vaccination according to risk [VaR].

The radar graph visualized the impact, efficiency, and uptake of each vaccination programme from 2024 – 2034, 2034 – 2044 and 2044 to 2054 respectively. Metrics were averted cases by percentage (A.P.), averted cases per dose (A.D.), number of vaccinations administered (N.V.), and primary uptake ratio (P.U.). Each sample’s metric value was normalized by the maximum and minimum across strategies for visual clarity, points closer to the center are closer to the overall strategy minimum metric score, and the radar border represents the overall maximum score each strategy can achieve.

**Variance decomposition on** $\boldsymbol{\eta}_{\boldsymbol{h}}$ **by keeping the parameter at the posterior median while allowing all other parameters to vary**

**Fig A16 Projected number of annual diagnosed cases and evaluation metrics by vaccination programme over 2024 – 2054 through variance decomposition on** $\boldsymbol{\eta}_{\boldsymbol{h}}$**.** Posterior means are marked with a square, and the 95% credible interval are indicated by the boundaries of the box. Model was calibrated to case series of male incidence multiplied by the proportion of reported gonorrhea cases in the UK MSM population. Projections assumed that transmission probabilities remained stable in 2024 – 2034 at 2018 levels. 1000 posterior draws were used to generate future projections and calculate programme-specific metrics. $\eta_{h}$was kept at median values while all other parameters were allowed to vary

The series of boxplots illustrate the annual future number of diagnosed cases under different vaccination strategies compared to a no-vaccination programme baseline. Each column represented a different vaccination programme. Respectively, these were denoted (**A**) vaccination before entry [VbE], (**B**) VoD strategies by offering vaccinations in these groups to only high sexual activity individuals (VoD[H]), (**C**) vaccination on diagnoses with gonorrhea [VoD], (**D**) vaccination on attendance [VoA], (**E**) VoA strategies by offering vaccinations in these groups to only high sexual activity individuals (VoA[H]) and (**F**) vaccination according to risk [VaR].

The radar graph visualized the impact, efficiency, and uptake of each vaccination programme from 2024 – 2034, 2034 – 2044 and 2044 to 2054 respectively. Metrics were averted cases by percentage (A.P.), averted cases per dose (A.D.), number of vaccinations administered (N.V.), and primary uptake ratio (P.U.). Each sample’s metric value was normalized by the maximum and minimum across strategies for visual clarity, points closer to the center are closer to the overall strategy minimum metric score, and the radar border represents the overall maximum score each strategy can achieve.

**Variance decomposition on the shape parameter of communicable disease surveillance data** $\boldsymbol{\kappa}_{\boldsymbol{d}}$ **by keeping the parameter at the posterior median while allowing all other parameters to vary**

**Fig A17 Projected number of annual diagnosed cases and evaluation metrics by vaccination programme over 2024 – 2054 through variance decomposition on** $\boldsymbol{\kappa}_{\boldsymbol{d}}$**.** Posterior means are marked with a square, and the 95% credible interval are indicated by the boundaries of the box. Model was calibrated to case series of male incidence multiplied by the proportion of reported gonorrhea cases in the UK MSM population. Projections assumed that transmission probabilities remained stable in 2024 – 2034 at 2018 levels. 1000 posterior draws were used to generate future projections and calculate programme-specific metrics. $\kappa_{d}$ was kept at median values while all other parameters were allowed to vary

The series of boxplots illustrate the annual future number of diagnosed cases under different vaccination strategies compared to a no-vaccination programme baseline. Each column represented a different vaccination programme. Respectively, these were denoted (**A**) vaccination before entry [VbE], (**B**) VoD strategies by offering vaccinations in these groups to only high sexual activity individuals (VoD[H]), (**C**) vaccination on diagnoses with gonorrhea [VoD], (**D**) vaccination on attendance [VoA], (**E**) VoA strategies by offering vaccinations in these groups to only high sexual activity individuals (VoA[H]) and (**F**) vaccination according to risk [VaR].

The radar graph visualized the impact, efficiency, and uptake of each vaccination programme from 2024 – 2034, 2034 – 2044 and 2044 to 2054 respectively. Metrics were averted cases by percentage (A.P.), averted cases per dose (A.D.), number of vaccinations administered (N.V.), and primary uptake ratio (P.U.). Each sample’s metric value was normalized by the maximum and minimum across strategies for visual clarity, points closer to the center are closer to the overall strategy minimum metric score, and the radar border represents the overall maximum score each strategy can achieve.

**Variance decomposition on** $\boldsymbol{\mu}$ **by keeping the parameter at the posterior median while allowing all other parameters to vary**

**Fig A18 Projected number of annual diagnosed cases and evaluation metrics by vaccination programme over 2024 – 2054 through variance decomposition on** $\boldsymbol{\mu}$**.** Posterior means are marked with a square, and the 95% credible interval are indicated by the boundaries of the box. Model was calibrated to case series of male incidence multiplied by the proportion of reported gonorrhea cases in the UK MSM population. Projections assumed that transmission probabilities remained stable in 2024 – 2034 at 2018 levels. 1000 posterior draws were used to generate future projections and calculate programme-specific metrics. $\mu$ was kept at median values while all other parameters were allowed to vary

The series of boxplots illustrate the annual future number of diagnosed cases under different vaccination strategies compared to a no-vaccination programme baseline. Each column represented a different vaccination programme. Respectively, these were denoted (**A**) vaccination before entry [VbE], (**B**) VoD strategies by offering vaccinations in these groups to only high sexual activity individuals (VoD[H]), (**C**) vaccination on diagnoses with gonorrhea [VoD], (**D**) vaccination on attendance [VoA], (**E**) VoA strategies by offering vaccinations in these groups to only high sexual activity individuals (VoA[H]) and (**F**) vaccination according to risk [VaR].

The radar graph visualized the impact, efficiency, and uptake of each vaccination programme from 2024 – 2034, 2034 – 2044 and 2044 to 2054 respectively. Metrics were averted cases by percentage (A.P.), averted cases per dose (A.D.), number of vaccinations administered (N.V.), and primary uptake ratio (P.U.). Each sample’s metric value was normalized by the maximum and minimum across strategies for visual clarity, points closer to the center are closer to the overall strategy minimum metric score, and the radar border represents the overall maximum score each strategy can achieve

**Variance decomposition on** $\boldsymbol{\nu}$ **by keeping the parameter at the mean while allowing all other parameters to vary**

**Fig A19 Projected number of annual diagnosed cases and evaluation metrics by vaccination programme over 2024 – 2054 through variance decomposition on** $\boldsymbol{\nu}$**.** Posterior means are marked with a square, and the 95% credible interval are indicated by the boundaries of the box. Model was calibrated to case series of male incidence multiplied by the proportion of reported gonorrhea cases in the UK MSM population. Projections assumed that transmission probabilities remained stable in 2024 – 2034 at 2018 levels. 1000 posterior draws were used to generate future projections and calculate programme-specific metrics. $\nu$ was kept at median values while all other parameters were allowed to vary

The series of boxplots illustrate the annual future number of diagnosed cases under different vaccination strategies compared to a no-vaccination programme baseline. Each column represented a different vaccination programme. Respectively, these were denoted (**A**) vaccination before entry [VbE], (**B**) VoD strategies by offering vaccinations in these groups to only high sexual activity individuals (VoD[H]), (**C**) vaccination on diagnoses with gonorrhea [VoD], (**D**) vaccination on attendance [VoA], (**E**) VoA strategies by offering vaccinations in these groups to only high sexual activity individuals (VoA[H]) and (**F**) vaccination according to risk [VaR].

The radar graph visualized the impact, efficiency, and uptake of each vaccination programme from 2024 – 2034, 2034 – 2044 and 2044 to 2054 respectively. Metrics were averted cases by percentage (A.P.), averted cases per dose (A.D.), number of vaccinations administered (N.V.), and primary uptake ratio (P.U.). Each sample’s metric value was normalized by the maximum and minimum across strategies for visual clarity, points closer to the center are closer to the overall strategy minimum metric score, and the radar border represents the overall maximum score each strategy can achieve.

**Variance decomposition on** $\boldsymbol{\omega}$ **by keeping the parameter at the posterior median while allowing all other parameters to vary**

**Fig A20 Projected number of annual diagnosed cases and evaluation metrics by vaccination programme over 2024 – 2054 through variance decomposition on** $\boldsymbol{\omega}$**.** Posterior means are marked with a square, and the 95% credible interval are indicated by the boundaries of the box. Model was calibrated to case series of male incidence multiplied by the proportion of reported gonorrhea cases in the UK MSM population. Projections assumed that transmission probabilities remained stable in 2024 – 2034 at 2018 levels. 1000 posterior draws were used to generate future projections and calculate programme-specific metrics. $\omega$ was kept at median values while all other parameters were allowed to vary

The series of boxplots illustrate the annual future number of diagnosed cases under different vaccination strategies compared to a no-vaccination programme baseline. Each column represented a different vaccination programme. Respectively, these were denoted (**A**) vaccination before entry [VbE], (**B**) VoD strategies by offering vaccinations in these groups to only high sexual activity individuals (VoD[H]), (**C**) vaccination on diagnoses with gonorrhea [VoD], (**D**) vaccination on attendance [VoA], (**E**) VoA strategies by offering vaccinations in these groups to only high sexual activity individuals (VoA[H]) and (**F**) vaccination according to risk [VaR].

The radar graph visualized the impact, efficiency, and uptake of each vaccination programme from 2024 – 2034, 2034 – 2044 and 2044 to 2054 respectively. Metrics were averted cases by percentage (A.P.), averted cases per dose (A.D.), number of vaccinations administered (N.V.), and primary uptake ratio (P.U.). Each sample’s metric value was normalized by the maximum and minimum across strategies for visual clarity, points closer to the center are closer to the overall strategy minimum metric score, and the radar border represents the overall maximum score each strategy can achieve.

**Variance decomposition on the initial prevalence of asymptomatics in the high risk group by keeping the parameter at the posterior median while allowing all other parameters to vary**

**Fig A21 Projected number of annual diagnosed cases and evaluation metrics by vaccination programme over 2024 – 2054 through variance decomposition on** $A_{H}\left( t0 \right)/N_{H}$**.** Posterior means are marked with a square, and the 95% credible interval are indicated by the boundaries of the box. Model was calibrated to case series of male incidence multiplied by the proportion of reported gonorrhea cases in the UK MSM population. Projections assumed that transmission probabilities remained stable in 2024 – 2034 at 2018 levels. 1000 posterior draws were used to generate future projections and calculate programme-specific metrics. The initial prevalence of asymptomatics in the high risk group was kept at median values while all other parameters were allowed to vary

The series of boxplots illustrate the annual future number of diagnosed cases under different vaccination strategies compared to a no-vaccination programme baseline. Each column represented a different vaccination programme. Respectively, these were denoted (**A**) vaccination before entry [VbE], (**B**) VoD strategies by offering vaccinations in these groups to only high sexual activity individuals (VoD[H]), (**C**) vaccination on diagnoses with gonorrhea [VoD], (**D**) vaccination on attendance [VoA], (**E**) VoA strategies by offering vaccinations in these groups to only high sexual activity individuals (VoA[H]) and (**F**) vaccination according to risk [VaR].

The radar graph visualized the impact, efficiency, and uptake of each vaccination programme from 2024 – 2034, 2034 – 2044 and 2044 to 2054 respectively. Metrics were averted cases by percentage (A.P.), averted cases per dose (A.D.), number of vaccinations administered (N.V.), and primary uptake ratio (P.U.). Each sample’s metric value was normalized by the maximum and minimum across strategies for visual clarity, points closer to the center are closer to the overall strategy minimum metric score, and the radar border represents the overall maximum score each strategy can achieve.

**Variance decomposition on the initial prevalence of asymptomatics in the low risk group by keeping the parameter at the posterior median while allowing all other parameters to vary**

**Fig A22 Projected number of annual diagnosed cases and evaluation metrics by vaccination programme over 2024 – 2054 through variance decomposition on** $A_{L}\left( t0 \right)/N_{L}$**.** Posterior means are marked with a square, and the 95% credible interval are indicated by the boundaries of the box. Model was calibrated to case series of male incidence multiplied by the proportion of reported gonorrhea cases in the UK MSM population. Projections assumed that transmission probabilities remained stable in 2024 – 2034 at 2018 levels. 1000 posterior draws were used to generate future projections and calculate programme-specific metrics. The initial prevalence of asymptomatics in the low-risk group was kept at median values while all other parameters were allowed to vary

The series of boxplots illustrate the annual future number of diagnosed cases under different vaccination strategies compared to a no-vaccination programme baseline. Each column represented a different vaccination programme. Respectively, these were denoted (**A**) vaccination before entry [VbE], (**B**) VoD strategies by offering vaccinations in these groups to only high sexual activity individuals (VoD[H]), (**C**) vaccination on diagnoses with gonorrhea [VoD], (**D**) vaccination on attendance [VoA], (**E**) VoA strategies by offering vaccinations in these groups to only high sexual activity individuals (VoA[H]) and (**F**) vaccination according to risk [VaR].

The radar graph visualized the impact, efficiency, and uptake of each vaccination programme from 2024 – 2034, 2034 – 2044 and 2044 to 2054 respectively. Metrics were averted cases by percentage (A.P.), averted cases per dose (A.D.), number of vaccinations administered (N.V.), and primary uptake ratio (P.U.). Each sample’s metric value was normalized by the maximum and minimum across strategies for visual clarity, points closer to the center are closer to the overall strategy minimum metric score, and the radar border represents the overall maximum score each strategy can achieve.

**Variance decomposition on** $\boldsymbol{\psi}$ **by keeping the parameter at the posterior median while allowing all other parameters to vary**

**Fig A23 Projected number of annual diagnosed cases and evaluation metrics by vaccination programme over 2024 – 2054 through variance decomposition on** $\boldsymbol{\psi}$**.** Posterior means are marked with a square, and the 95% credible interval are indicated by the boundaries of the box. Model was calibrated to case series of male incidence multiplied by the proportion of reported gonorrhea cases in the UK MSM population. Projections assumed that transmission probabilities remained stable in 2024 – 2034 at 2018 levels. 1000 posterior draws were used to generate future projections and calculate programme-specific metrics. $\psi$ was kept at median values while all other parameters were allowed to vary

The series of boxplots illustrate the annual future number of diagnosed cases under different vaccination strategies compared to a no-vaccination programme baseline. Each column represented a different vaccination programme. Respectively, these were denoted (**A**) vaccination before entry [VbE], (**B**) VoD strategies by offering vaccinations in these groups to only high sexual activity individuals (VoD[H]), (**C**) vaccination on diagnoses with gonorrhea [VoD], (**D**) vaccination on attendance [VoA], (**E**) VoA strategies by offering vaccinations in these groups to only high sexual activity individuals (VoA[H]) and (**F**) vaccination according to risk [VaR].

The radar graph visualized the impact, efficiency, and uptake of each vaccination programme from 2024 – 2034, 2034 – 2044 and 2044 to 2054 respectively. Metrics were averted cases by percentage (A.P.), averted cases per dose (A.D.), number of vaccinations administered (N.V.), and primary uptake ratio (P.U.). Each sample’s metric value was normalized by the maximum and minimum across strategies for visual clarity, points closer to the center are closer to the overall strategy minimum metric score, and the radar border represents the overall maximum score each strategy can achieve.

**Variance decomposition on** $\boldsymbol{\rho}$ **by keeping the parameter at the posterior median while allowing all other parameters to vary**

**Fig A24 Projected number of annual diagnosed cases and evaluation metrics by vaccination programme over 2024 – 2054 through variance decomposition on** $\boldsymbol{\rho}$**.** Posterior means are marked with a square, and the 95% credible interval are indicated by the boundaries of the box. Model was calibrated to case series of male incidence multiplied by the proportion of reported gonorrhea cases in the UK MSM population. Projections assumed that transmission probabilities remained stable in 2024 – 2034 at 2018 levels. 1000 posterior draws were used to generate future projections and calculate programme-specific metrics. $\nu$ was kept at median values while all other parameters were allowed to vary

The series of boxplots illustrate the annual future number of diagnosed cases under different vaccination strategies compared to a no-vaccination programme baseline. Each column represented a different vaccination programme. Respectively, these were denoted (**A**) vaccination before entry [VbE], (**B**) VoD strategies by offering vaccinations in these groups to only high sexual activity individuals (VoD[H]), (**C**) vaccination on diagnoses with gonorrhea [VoD], (**D**) vaccination on attendance [VoA], (**E**) VoA strategies by offering vaccinations in these groups to only high sexual activity individuals (VoA[H]) and (**F**) vaccination according to risk [VaR].

The radar graph visualized the impact, efficiency, and uptake of each vaccination programme from 2024 – 2034, 2034 – 2044 and 2044 to 2054 respectively. Metrics were averted cases by percentage (A.P.), averted cases per dose (A.D.), number of vaccinations administered (N.V.), and primary uptake ratio (P.U.). Each sample’s metric value was normalized by the maximum and minimum across strategies for visual clarity, points closer to the center are closer to the overall strategy minimum metric score, and the radar border represents the overall maximum score each strategy can achieve.

**Variance decomposition on** $\boldsymbol{\sigma}$ **by keeping the parameter at the posterior median while allowing all other parameters to vary**

**Fig A25 Projected number of annual diagnosed cases and evaluation metrics by vaccination programme over 2024 – 2054 through variance decomposition on .** Posterior means are marked with a square, and the 95% credible interval are indicated by the boundaries of the box. Model was calibrated to case series of male incidence multiplied by the proportion of reported gonorrhea cases in the UK MSM population. Projections assumed that transmission probabilities remained stable in 2024 – 2034 at 2018 levels. 1000 posterior draws were used to generate future projections and calculate programme-specific metrics. $\rho$ was kept at median values while all other parameters were allowed to vary

The series of boxplots illustrate the annual future number of diagnosed cases under different vaccination strategies compared to a no-vaccination programme baseline. Each column represented a different vaccination programme. Respectively, these were denoted (**A**) vaccination before entry [VbE], (**B**) VoD strategies by offering vaccinations in these groups to only high sexual activity individuals (VoD[H]), (**C**) vaccination on diagnoses with gonorrhea [VoD], (**D**) vaccination on attendance [VoA], (**E**) VoA strategies by offering vaccinations in these groups to only high sexual activity individuals (VoA[H]) and (**F**) vaccination according to risk [VaR].

The radar graph visualized the impact, efficiency, and uptake of each vaccination programme from 2024 – 2034, 2034 – 2044 and 2044 to 2054 respectively. Metrics were averted cases by percentage (A.P.), averted cases per dose (A.D.), number of vaccinations administered (N.V.), and primary uptake ratio (P.U.). Each sample’s metric value was normalized by the maximum and minimum across strategies for visual clarity, points closer to the center are closer to the overall strategy minimum metric score, and the radar border represents the overall maximum score each strategy can achieve.

**Reference:**

1. Garnett GP, Mertz KJ, Finelli L, Levine WC, St Louis ME. The transmission dynamics of gonorrhea: modelling the reported behaviour of infected patients from Newark, New Jersey. Philos Trans R Soc B Biol Sci. 1999 Apr 29;354(1384):787–97.

2. Whittles LK, Didelot X, White PJ. Public health impact and cost-effectiveness of gonorrhea vaccination: an integrated transmission-dynamic health-economic modelling analysis. Lancet Infect Dis. 2022 Jul 1;22(7):1030–41.

3. MOH | Communicable Diseases Surveillance in Singapore 2019-2020 [Internet]. [cited 2024 May 15]. Available from: https://www.moh.gov.sg/resources-statistics/reports/communicable-diseases-surveillance-in-singapore-2019-2020

4. Cameron AC, Trivedi PK. Regression-based tests for overdispersion in the Poisson model. J Econom. 1990 Dec 1;46(3):347–64.

5. Quaye SED, Cheng Y, Tan RKJ, Koo JR, Prem K, Teo AKJ, et al. Application of the network scale-up method to estimate the sizes of key populations for HIV in Singapore using online surveys. J Int AIDS Soc. 2023 Mar;26(3):e25973.

6. Johnson, A., Mercer, C., Sonnenberg, P., Copas, A., Wellings, K., Macdowall, W., Erens, B. National Survey of Sexual Attitudes and Lifestyles, 2010-2012. [data collection]. 2nd Edition. UK Data Service. SN: 7799. 2024;

7. Whittles LK, White PJ, Didelot X. Assessment of the Potential of Vaccination to Combat Antibiotic Resistance in Gonorrhea: A Modeling Analysis to Determine Preferred Product Characteristics. Clin Infect Dis. 2020 Nov 5;71(8):1912–9.

8. Population and Population Structure [Internet]. Statistics on Singapore’s population are compiled by the Singapore Department of Statistics. [cited 2024 Jul 11]. Available from: http://www.singstat.gov.sg/find-data/search-by-theme/population/population-and-population-structure/latest-data

9. Molina JM, Bercot B, Assoumou L, Rubenstein E, Algarte-Genin M, Pialoux G, et al. Doxycycline prophylaxis and meningococcal group B vaccine to prevent bacterial sexually transmitted infections in France (ANRS 174 DOXYVAC): a multicentre, open-label, randomised trial with a 2 × 2 factorial design. Lancet Infect Dis. 2024 May;S1473309924002366.

10. Effectiveness of a group B outer membrane vesicle meningococcal vaccine against gonorrhea in New Zealand: a retrospective case-control study - ClinicalKey [Internet]. [cited 2024 May 20]. Available from: https://www-clinicalkey-com.remotexs.ntu.edu.sg/#!/content/playContent/1-s2.0-S0140673617314496?returnurl=null&referrer=null

11. Robison SG, Leman RF. Association of Group B Meningococcal Vaccine Receipt With Reduced Gonorrhea Incidence Among University Students. JAMA Netw Open. 2023 Aug 31;6(8): e2331742.

12. Longtin J, Dion R, Simard M, Betala Belinga JF, Longtin Y, Lefebvre B, et al. Possible Impact of Wide-scale Vaccination Against Serogroup B Neisseria Meningitidis on Gonorrhea Incidence Rates in One Region of Quebec, Canada. Open Forum Infect Dis. 2017 Oct 1;4(suppl_1): S734–5.

13. Santolaya ME, O’Ryan M, Valenzuela MT, Prado V, Vergara RF, Muñoz A, et al. Persistence of antibodies in adolescents 18−24 months after immunization with one, two, or three doses of 4CMenB meningococcal serogroup B vaccine. Hum Vaccines Immunother. 2013 Nov 1;9(11): 2304–10.

14. Nolan T, Santolaya ME, de Looze F, Marshall H, Richmond P, Henein S, et al. Antibody persistence and booster response in adolescents and young adults 4 and 7.5 years after immunization with 4CMenB vaccine. Vaccine. 2019 Feb 21;37(9):1209–18.

15. Public Health England. Human papillomavirus (HPV) vaccination uptake in gay, bisexual and other men who have sex with men (MSM). National programme: 2018 annual report. [Internet]. 2020. Available from: https://assets.publishing.service.gov.uk/government/uploads/system/uploads/attachment_data/file/864727/HPV_MSM_programme_report_2018.pdf

**Supporting Information legend**

Fig A1: Model-structure diagram for characterizing gonorrhea transmission.

Table A1: Annual gonorrhea diagnosed in Singapore MSM under different scenarios.

Table A2: Fixed parameters for Singapore gonorrhea transmission model.

Table A3: Fitted demographic parameters: notation, definition, prior distribution, and posterior estimates.

Table A4: Fitted transmission parameters: notation, definition, prior distribution, parameter bounds, and posterior estimates.

Fig A2: Trace plots of posterior parameter estimates under different observation scenarios.

Fig A3: Comparison of gonorrhea annual diagnosed projection from calibrated model and observation under different scenarios.

Table A5: Vaccination parameters used in sensitivity analysis.

Table A6: Summary of individuals entering each model compartment in the projected scenario of decreasing transmission probabilities.

Table A7: Summary of population health impact for various vaccination programmes from 2024 to 2034.

Fig A4: Projected number of annual diagnosed cases and evaluation metrics by vaccination programme over 2024 – 2034 with decreasing transmission probability, by model calibrated to the case series of male incidence less the female incidence of gonorrhea.

Table A8: Summary of individuals entering each model compartment in projected scenario of stable transmission probabilities.

Table A9: Summary of population health impact for various vaccination programmes from 2024 to 2034.

Fig A5: Projected number of annual diagnosed cases and evaluation metrics by vaccination programme over 2024 – 2034 with transmission probability set to that of 2018, by model calibrated to the case series of male incidence of gonorrhea.

Table A10: Summary of individuals entering each model compartment in projected scenario of decreasing transmission probabilities.

Table A11: Summary of population health impact for various vaccination programmes from 2024 to 2034.

Fig A6: Projected number of annual diagnosed cases and evaluation metrics by vaccination programme over 2024 – 2034 with decreasing transmission probability, by model calibrated to the case series of male incidence of gonorrhea.

Table A12: Summary of individuals entering each model compartment in projected scenario of stable transmission probabilities.

Table A13: Summary of population health impact for various vaccination programmes from 2024 to 2034.

Fig A7: Projected number of annual diagnosed cases and evaluation metrics by vaccination programme over 2024 – 2034 with transmission probability set to that of 2018, by model calibrated to the case series of male incidence multiplied by the proportion of reported gonorrhea cases in the UK MSM population.

Table A14: Summary of individuals entering each model compartment in projected scenario of stable transmission probabilities.

Table A15: Summary of population health impact for various vaccination programmes from 2024 to 2034.

Fig A8 Projected number of annual diagnosed cases and evaluation metrics by vaccination programme over 2024 – 2034 with decreasing transmission probability, by model calibrated to the case series of male incidence multiplied by the proportion of reported gonorrhea cases in the UK MSM population.

Fig A9: Comparison of gonorrhea annual diagnosed projection from calibrated model and observation under an exponentially decaying rate of transmission.

Fig A10 Projected number of annual diagnosed cases and evaluation metrics by vaccination programme over 2024 – 2034 under hypothetically increasing number of population entrants (1% increase per year).

Fig A11 Projected number of annual diagnosed cases and evaluation metrics by vaccination programme over 2024 – 2034 under hypothetically decreasing number of population entrants (-1% increase per year).

Fig A12: Fitted and predicted values of gonorrhea incidences from the calibrated model.

Table A16: Mean absolute percentage errors of model in out-of-sample prediction of annual gonnorhea incidences.

Table A17: Summary of population health impact for various vaccination programmes from 2024 to 2034, with a -50% change in the assortativity parameter.

Table A18: Summary of population health impact for various vaccination programmes from 2024 to 2034, with a -25% change in the assortativity parameter.

Table A19: Summary of population health impact for various vaccination programmes from 2024 to 2034, with a +25% change in the assortativity parameter.

Table A20: Summary of population health impact for various vaccination programmes from 2024 to 2034, with a +50% change in the assortativity parameter.

Table A21: Summary of averted cases (total) for various vaccination programmes from 2024 to 2034, by varying the assortativity parameter ($\epsilon$).

Table A22: Summary of averted cases by percentage for various vaccination programmes from 2024 to 2034, by varying the assortativity parameter ($\epsilon$).

Table A23: Summary of averted cases per dose for various vaccination programmes from 2024 to 2034, by varying the assortativity parameter ($\epsilon$).

Table A24: Summary of population health impact for various vaccination programmes from 2024 to 2034, with a -50% change in the asymptomatic screening rate.

Table A25: Summary of population health impact for various vaccination programmes from 2024 to 2034, with a -25% change in the asymptomatic screening rate.

Table A26: Summary of population health impact for various vaccination programmes from 2024 to 2034, with a +25% change in the asymptomatic screening rate.

Table A27: Summary of population health impact for various vaccination programmes from 2024 to 2034, with a +50% change in the asymptomatic screening rate.

Table A28: Summary of population health impact for various vaccination programmes from 2024 to 2034, with a -50% change in the rate of seeking treatment due to symptoms.

Table A29: Summary of population health impact for various vaccination programmes from 2024 to 2034, with a -25% change in the rate of seeking treatment due to symptoms.

Table A30: Summary of population health impact for various vaccination programmes from 2024 to 2034, with a +25% change in the rate of seeking treatment due to symptoms.

Table A31: Summary of population health impact for various vaccination programmes from 2024 to 2034, with a +50% change in the rate of seeking treatment due to symptoms.

Table A32: Summary of population health impact for various vaccination programmes from 2024 to 2034, with a -50% change in the natural recovery rate.

Table A33: Summary of population health impact for various vaccination programmes from 2024 to 2034, with a -25% change in the natural recovery rate.

Table A34: Summary of population health impact for various vaccination programmes from 2024 to 2034, with a +25% change in the natural recovery rate.

Table A35: Summary of population health impact for various vaccination programmes from 2024 to 2034, with a +50% change in the natural recovery rate.

Table A36: Summary of population health impact for various vaccination programmes from 2024 to 2034, with a -50% change in the screening rate in low versus high groups.

Table A37: Summary of population health impact for various vaccination programmes from 2024 to 2034, with a -25% change in the rate of seeking treatment due to symptoms.

Table A38: Summary of population health impact for various vaccination programmes from 2024 to 2034, with a +25% change in the rate of seeking treatment due to symptoms.

Table A39: Summary of population health impact for various vaccination programmes from 2024 to 2034, with a +50% change in the rate of seeking treatment due to symptoms.

Table A40: Summary of population health impact for various vaccination programmes from 2024 to 2034, with a -50% change in the initial prevalence of asymptomatic infection in the low risk group.

Table A41: Summary of population health impact for various vaccination programmes from 2024 to 2034, with a -25% change in the initial prevalence of asymptomatic infection in the low risk group.

Table A42: Summary of population health impact for various vaccination programmes from 2024 to 2034, with a +25% change in the initial prevalence of asymptomatic infection in the low risk group.

Table A43: Summary of population health impact for various vaccination programmes from 2024 to 2034, with a +50% change in the initial prevalence of asymptomatic infection in the low risk group.

Table A44: Summary of population health impact for various vaccination programmes from 2024 to 2034, with a -50% change in the initial prevalence of asymptomatic infection in the high risk group.

Table A45: Summary of population health impact for various vaccination programmes from 2024 to 2034, with a -25% change in initial prevalence of asymptomatic infection in the high risk group.

Table A46: Summary of population health impact for various vaccination programmes from 2024 to 2034, with a +25% change in the initial prevalence of asymptomatic infection in the high risk group.

Table A47: Summary of population health impact for various vaccination programmes from 2024 to 2034, with a +50% change in the initial prevalence of asymptomatic infection in the high risk group.

Table A48: Summary of population health impact for various vaccination programmes from 2024 to 2034, with a -50% change in the number of asymptomatics per incident infection.

Table A49: Summary of population health impact for various vaccination programmes from 2024 to 2034, with a -25% change in the number of asymptomatics per incident infection.

Table A50: Summary of population health impact for various vaccination programmes from 2024 to 2034, with a +25% change in the number of asymptomatics per incident infection.

Table A51: Summary of population health impact for various vaccination programmes from 2024 to 2034, with a +50% change in the number of asymptomatics per incident infection.

Table A52: Summary of population health impact for various vaccination programmes from 2024 to 2034, with a -50% change in the partner change rate for the low risk group.

Table A53: Summary of population health impact for various vaccination programmes from 2024 to 2034, with a -25% change in the partner change rate for the low risk group.

Table A54: Summary of population health impact for various vaccination programmes from 2024 to 2034, with a +25% change in the partner change rate for the low risk group.

Table A55: Summary of population health impact for various vaccination programmes from 2024 to 2034, with a +50% change in the partner change rate for the low risk group.\

Table A56: Summary of population health impact for various vaccination programmes from 2024 to 2034, with a -50% change in the partner change rate for the high risk group.

Table A57: Summary of population health impact for various vaccination programmes from 2024 to 2034, with a -25% change in the partner change rate for the high risk group.

Table A58: Summary of population health impact for various vaccination programmes from 2024 to 2034, with a +25% change in the partner change rate for the high risk group.

Table A59: Summary of population health impact for various vaccination programmes from 2024 to 2034, with a +50% change in the partner change rate for the high risk group.

Table A60: Summary of population health impact for various vaccination programmes from 2024 to 2034, with the proportion of individuals in the high risk group changed to 0.1.

Table A61: Summary of population health impact for various vaccination programmes from 2024 to 2034, with the proportion of individuals in the high risk group changed to 0.2.

Table A62: Summary of population health impact for various vaccination programmes from 2024 to 2034, with the proportion of individuals in the high risk group changed to 0.3.

Fig A13 Projected number of annual diagnosed cases and evaluation metrics by vaccination programme over 2024 – 2054 through variance decomposition on $\beta_{2004}$.

Fig A14 Projected number of annual diagnosed cases and evaluation metrics by vaccination programme over 2024 – 2054 through variance decomposition on $\beta_{2018}$.

Fig A15 Projected number of annual diagnosed cases and evaluation metrics by vaccination programme over 2024 – 2054 through variance decomposition on $\epsilon$.

Fig A16 Projected number of annual diagnosed cases and evaluation metrics by vaccination programme over 2024 – 2054 through variance decomposition on $\eta_{h}$.

Fig A17 Projected number of annual diagnosed cases and evaluation metrics by vaccination programme over 2024 – 2054 through variance decomposition on $\kappa_{d}$.

Fig A18 Projected number of annual diagnosed cases and evaluation metrics by vaccination programme over 2024 – 2054 through variance decomposition on $\mu$.

Fig A19 Projected number of annual diagnosed cases and evaluation metrics by vaccination programme over 2024 – 2054 through variance decomposition on $\nu$.

Fig A20 Projected number of annual diagnosed cases and evaluation metrics by vaccination programme over 2024 – 2054 through variance decomposition on $\omega$.

Fig A21 Projected number of annual diagnosed cases and evaluation metrics by vaccination programme over 2024 – 2054 through variance decomposition on $A_{H}\left( t0 \right)/N_{H}$.

Fig A22 Projected number of annual diagnosed cases and evaluation metrics by vaccination programme over 2024 – 2054 through variance decomposition on $A_{L}\left( t0 \right)/N_{L}$.

Fig A23 Projected number of annual diagnosed cases and evaluation metrics by vaccination programme over 2024 – 2054 through variance decomposition on $\psi$.

Fig A24 Projected number of annual diagnosed cases and evaluation metrics by vaccination programme over 2024 – 2054 through variance decomposition on $\rho$.

Fig A25 Projected number of annual diagnosed cases and evaluation metrics by vaccination programme over 2024 – 2054 through variance decomposition on $\sigma$.
