# Supplementary material for: Potential public health impacts of gonorrhea vaccination programmes under declining incidences: A modeling study
Source: PLoS Med. 2025 Feb 7;22(2):e1004521. doi: 10.1371/journal.pmed.1004521 (PMC11805383; doi:10.1371/journal.pmed.1004521)
Supplement: Attachment — Submitted filename: Response_Document_08112024.docx [file pmed.1004521.s004.docx]

**Editor Comments**

Dear Dr Geng,

Many thanks for submitting your manuscript "Potential public health impacts of gonorrhoea vaccination programmes under declining incidences: a modelling analysis" (PMEDICINE-D-24-02533R1) to PLOS Medicine. The paper has been reviewed by subject experts and a statistician; their comments are included below and can also be accessed here: 

As you will see, the value of your work was evident to many, though the reviewers did raise a number of points to address. After discussing the paper with the editorial team and an academic editor with relevant expertise, I'm pleased to invite you to revise the paper in response to the reviewers' comments. We plan to send the revised paper to some or all of the original reviewers, and we cannot provide any guarantees at this stage regarding publication.

When you upload your revision, please include a point-by-point response that addresses all of the reviewer and editorial points, indicating the changes made in the manuscript and either an excerpt of the revised text or the location (eg: page and line number) where each change can be found. Please also be sure to check the general editorial comments at the end of this letter and include these in your point-by-point response. When you resubmit your paper, please include a clean version of the paper as the main article file and a version with changes tracked as a marked-up manuscript. It may also be helpful to check the guidelines for revised papers at <http://journals.plos.org/plosmedicine/s/revising-your-manuscript> for any that apply to your paper.

We ask that you submit your revision by Nov 08 2024 11:59PM. However, if this deadline is not feasible, please contact me by email, and we can discuss a suitable alternative.

Don't hesitate to contact me directly with any questions ([ssunny@plos.org](mailto:ssunny@plos.org)). 

Best regards, 
Syba 

Syba Sunny, MBBS, MRes, FRCPath 
Associate Editor
PLOS Medicine
[ssunny@plos.org](mailto:ssunny@plos.org)

-----------------------------------------------------------
Comments from the academic editor:

The academic editor was supportive of your manuscript and wrote this: ‘The work is important and novel - when there is uncertainty in the field, mathematical models can provide insights into potential scenarios as outlined by the reviewers including the (fundamental) uncertainty of the efficacy of the meningococcal vaccine and GC resistance to antibiotics. Analyses can also inform the ideal product profile for gonorrhea vaccine development.’ She agreed with the reviewer comments and asked that you were given the opportunity to respond to them.

**Response:** We thank the editors for the comments and the opportunity to revise the manuscript entitled
*“Potential public health impacts of gonorrhoea vaccination programmes under declining incidences: a modelling analysis”.* Below, we respond point by point to each of the reviewers’ comments,. We have outlined potential sources of uncertainty through an extensive sensitivity analysis of epidemiological and behavioural parameters used in our model, as well as through validation checks and decomposing the sources of variance in *our model:*

*“****Data*** *The model was calibrated using a Bayesian inference framework using proxies of annual gonorrhoea MSM incidence from 2004 to 2018 in Singapore. MSMs were defined to be men who report ever having sex with a man. As actual breakdowns of annual gonorrhoea MSM incidence were not available in the study setting, we assumed that this was proxied by (****1****) male incidence less the female incidence of gonorrhoea (****2****) the male incidence of gonorrhoea, i.e the upper bound to the annual gonorrhoea MSM incidence (****3****) the male incidence multiplied by the proportion of reported gonorrhoea cases in the MSM population in other study settings (See supplementary information, Table S1). Raw annual gonorrhoea incidence was obtained from the communicable disease surveillance system of Singapore from 2004-2020 [1] and excluded observations in 2019, 2020 due to COVID-19. We calibrated our model under each scenario and reported the first scenario as our main results…..”*

*“….We separately calibrated the transmission models based on the different proxies of MSM gonorrhea incidence (see* ***Data*** *above). We projected the future incidences based on constant or linearly trending transmission rates over 2024 – 2034 and assessed the influence of these separate models on vaccination programmes’ public health impacts over the projected time horizon. Furthermore, in order to ascertain how the uncertainty in different parameters contribute to the overall uncertainty of the results, we iteratively set each parameter by its median values and all other parameters to vary according to the sampled distribution. Thereafter each the vaccination programmes’s impacts over a 10-30year timeframe was projected, under the 3 separate case series.*

*We further assessed the predictive ability of our models through an expanding cross-validation scheme, where the data was split to initial testing and training sets (70%, 30%), and the model was calibrated to the training set and annual gonnorhoea incidence rates projected forward in time to assess concordance with the test-set observations. In the subsequent cross-validation steps, an additional year of data was added in and the procedure is repeated till the testing set is depleted. Results were inspected visually through whether the model could capture the general trends in annual gonorrhoea incidence rates in the test set, and quantified through mean absolute percentage errors.*

*Lastly, we examined the sensitivity of behavioural and epidemiological parameters on our model’s outputs of the prospective vaccination strategies efficiency and raw public health impacts. This was done by varying the parameters (i.e, screening rates, recovery rates, asymptomatic rates, proportion of individuals in high/low risk groups) by* $\pm$*25,50% individually while keeping all other parameters fixed where possible and reprojecting each vaccination strategy through 2024–2034…..”*

**Reviewer 1**

**Comment:** In this paper, the author developed and calibrated a simulation model to examine the impact of various vaccination strategies on gonorrhea incidence. The model was specifically applied to the MSM population in Singapore. The author concluded that vaccinating MSM against gonorrhea based on risk assessment in sexual health clinics in Singapore is a viable approach, and that vaccine development efforts should prioritize maximizing efficacy over the duration of protection. The topic is innovative, and the findings make a significant contribution to the existing literature. I recommend that this paper be considered for publication after the following concerns are addressed.

**Response:** We thank the reviewer for the comments. Below, we provide our point-by-point responses to the comments given by the reviewer and hope that the amendments to the manuscript provide clarity and cogency to our findings.

**Comment:** In the model structure, the author assumed that all individuals entering treatment can be successfully treated. However, for gonorrhoea transmission, particularly within MSM populations, antibiotic resistance is a critical issue. While the author noted that few resistant strains have been diagnosed in Singapore, I recommend that antibiotic resistance should be considered due to the uncertainty surrounding future transmission dynamics. I suggest adding a resistance compartment to the simulation model or conducting a sensitivity analysis on resistance scenarios, with a discussion of the corresponding results. It could be insightful to examine the impact of antibiotics not only on transmission but also on resistance. Incorporating this aspect would enhance the model's applicability to gonorrhoea transmission in other regions and strengthen the overall conclusions.

**Response:** We thank the reviewer for the comment. We agree with the reviewer that antibiotic resistance may impact the burden of disease. In particular, increasing the time in which treated individuals recover from disease. Rates of AMR in the local population however were lower compared to the broader region in which Singapore resides (~1.5% as of 2018) [2]. It is also difficult to calibrate the model by incorporating a separate time-series of individuals infected by antibiotic-resistant gonorrhoea when case counts are extremely low in this population. However, increasing rates of AMR may potentially decrease the rate of recovery in individuals due to decreased treatment options. We have ran a sensitivity analysis to show that a moderate decrease in the time needed for recovery after treatment to proxy a decreased rate of AMR in gonorrhoea by varying this parameter by -50 to 25%, and found that number of averted cases can potentially be higher by employing vaccination strategies:

| **Strategy** | **Averted cases**  **(total)** | **Averted cases**  **(%)** | **Averted cases**  **per dose** | **Number of vaccinations administered** | **Primary**  **uptake ratio** |
| --- | --- | --- | --- | --- | --- |
| VbE | 1611 (927-2066) | 14.12 (9.08-17.01) | 0.005 (0.003-0.006) | 172701 (172701-172701) | 100.00 (100.00-100.00) |
| VoD(H) | 1375 (536-2077) | 11.81 (7.03-14.67) | 0.24 (0.14-0.31) | 2910 (1651-3855) | 96.85 (95.54-97.73) |
| VoD | 1375 (536-2077) | 11.82 (7.03-14.67) | 0.21 (0.12-0.27) | 3268 (1822-4199) | 97.17 (95.69-98.00) |
| VoA | 4594 (1905-6781) | 40.05 (18.15-52.35) | 0.06 (0.02-0.09) | 45674 (12968-73303) | 91.63 (85.35-95.58) |
| VoA(H) | 4575 (1894-6771) | 39.88 (17.97-52.33) | 0.21 (0.12-0.28) | 11600 (5467-15643) | 87.15 (79.56-92.27) |
| VaR | 4575 (1894-6771) | 39.88 (17.98-52.33) | 0.21 (0.12-0.27) | 11848 (5865-15835) | 87.42 (79.75-92.63) |

**Response Table Summary of population health impact for various vaccination programmes from 2024 to 2034, with a -50% change in the rate of recovery after treatment** We report posterior means and accompanying 95% credible intervals. Estimates presented are relative to the baseline scenario of no intervention, with transmission probabilities from 2024 to 2034 kept stable at 2018 levels.

| **Strategy** | **Averted cases**  **(total)** | **Averted cases**  **(%)** | **Averted cases**  **per dose** | **Number of vaccinations administered** | **Primary**  **uptake ratio** |
| --- | --- | --- | --- | --- | --- |
| VbE | 1635 (937-2102) | 14.16 (9.10-17.06) | 0.005 (0.003-0.006) | 172701 (172701-172701) | 100.00 (100.00-100.00) |
| VoD(H) | 1409 (546-2146) | 11.96 (7.09-14.89) | 0.24 (0.14-0.31) | 2939 (1666-3893) | 96.81 (95.49-97.70) |
| VoD | 1410 (546-2146) | 11.97 (7.09-14.89) | 0.21 (0.12-0.28) | 3301 (1842-4234) | 97.14 (95.65-97.97) |
| VoA | 4667 (1933-6889) | 40.19 (18.26-52.50) | 0.06 (0.02-0.10) | 45691 (12992-73319) | 91.62 (85.35-95.57) |
| VoA(H) | 4647 (1922-6872) | 40.02 (18.08-52.48) | 0.22 (0.12-0.28) | 11615 (5490-15660) | 87.14 (79.55-92.25) |
| VaR | 4648 (1923-6872) | 40.03 (18.09-52.48) | 0.21 (0.12-0.27) | 11865 (5892-15846) | 87.40 (79.74-92.62) |

**Response Table Summary of population health impact for various vaccination programmes from 2024 to 2034, with a -25% change in the rate of recovery after treatment** We report posterior means and accompanying 95% credible intervals. Estimates presented are relative to the baseline scenario of no intervention, with transmission probabilities from 2024 to 2034 kept stable at 2018 levels.

| **Strategy** | **Averted cases**  **(total)** | **Averted cases**  **(%)** | **Averted cases**  **per dose** | **Number of vaccinations administered** | **Primary**  **uptake ratio** |
| --- | --- | --- | --- | --- | --- |
| VbE | 1655 (945-2131) | 14.19 (9.12-17.09) | 0.005 (0.003-0.006) | 172701 (172701-172701) | 100.00 (100.00-100.00) |
| VoD(H) | 1438 (554-2195) | 12.09 (7.14-15.06) | 0.24 (0.14-0.32) | 2963 (1678-3924) | 96.78 (95.46-97.69) |
| VoD | 1438 (554-2195) | 12.09 (7.14-15.06) | 0.22 (0.12-0.28) | 3327 (1860-4262) | 97.12 (95.61-97.96) |
| VoA | 4727 (1957-6980) | 40.30 (18.35-52.62) | 0.06 (0.02-0.10) | 45705 (13011-73332) | 91.62 (85.35-95.56) |
| VoA(H) | 4707 (1949-6952) | 40.14 (18.17-52.59) | 0.22 (0.12-0.29) | 11627 (5509-15675) | 87.12 (79.54-92.23) |
| VaR | 4708 (1949-6952) | 40.14 (18.18-52.59) | 0.21 (0.12-0.27) | 11879 (5915-15855) | 87.39 (79.73-92.61) |

**Response Table Summary of population health impact for various vaccination programmes from 2024 to 2034, with a +25% change in the rate of recovery after treatment** We report posterior means and accompanying 95% credible intervals. Estimates presented are relative to the baseline scenario of no intervention, with transmission probabilities from 2024 to 2034 kept stable at 2018 levels.

| **Strategy** | **Averted cases**  **(total)** | **Averted cases**  **(%)** | **Averted cases**  **per dose** | **Number of vaccinations administered** | **Primary**  **uptake ratio** |
| --- | --- | --- | --- | --- | --- |
| VbE | 1660 (946-2138) | 14.19 (9.12-17.10) | 0.005 (0.003-0.006) | 172701 (172701-172701) | 100.00 (100.00-100.00) |
| VoD(H) | 1445 (556-2208) | 12.12 (7.15-15.10) | 0.24 (0.14-0.32) | 2969 (1681-3932) | 96.78 (95.45-97.68) |
| VoD | 1446 (556-2208) | 12.12 (7.15-15.10) | 0.22 (0.12-0.28) | 3334 (1864-4270) | 97.11 (95.61-97.95) |
| VoA | 4742 (1963-7000) | 40.33 (18.37-52.65) | 0.06 (0.02-0.10) | 45708 (13016-73336) | 91.61 (85.35-95.56) |
| VoA(H) | 4722 (1955-6973) | 40.17 (18.19-52.62) | 0.22 (0.12-0.29) | 11630 (5513-15678) | 87.12 (79.54-92.23) |
| VaR | 4723 (1955-6974) | 40.17 (18.20-52.62) | 0.21 (0.12-0.27) | 11883 (5920-15857) | 87.39 (79.73-92.61) |

**Response Table Summary of population health impact for various vaccination programmes from 2024 to 2034, with a +50% change in the rate of recovery after treatment** We report posterior means and accompanying 95% credible intervals. Estimates presented are relative to the baseline scenario of no intervention, with transmission probabilities from 2024 to 2034 kept stable at 2018 levels.

**Comment:** In the discussion, the author mentioned that the cost-effectiveness of the vaccination strategies was not analyzed due to the lack of publicly available cost data on gonorrhea treatment. However, Li et al. (2022) estimated the attributable disease burden in terms of discounted lifetime costs and quality-adjusted life-years (QALYs) lost for the United States. The author may want to revise this section of the discussion to clarify this point and avoid potential confusion.

**Response:** We thank the reviewer for the comment. We have amended this statement to be clear that there is no publicly available cost data on gonorrhea treatment in the study setting (Singapore):

*“However, in our exploration of the public health impacts of these vaccination programmes, we did not study the cost-effectiveness of these strategies, which departed from prior modelling work done in settings of historical increasing incidence settings [3], due to the lack of publicly available cost data on gonorrhoea treatment in the study setting”*

**Reviewer 2**

**Comment:** Thank you for the opportunity to review this paper. My comments are below.

**Response:** We thank the reviewer for the comments. Below, we provide our point-by-point responses to the comments given by the reviewer and hope that the amendments to the manuscript provide clarity and cogency to our findings.

**Comment:** In the methods section, revise "…reducing gonorrhea transmission in the men who have sex with men population…". Suggest changing to "…reducing gonorrhea transmission in men who have sex with men…"

**Response:** We thank the reviewer for the comment. We have now amended this statement:

*“We employed an integrated transmission-dynamic model, calibrated using Bayesian methods to local surveillance data to understand the potential public health impact of 4CMenB in reducing gonorrhea transmission in men who have sex with men (MSM) in Singapore”*

**Comment:** In the methods section, delete "to" in the following sentences: "…offering vaccination to individuals attending to sexual health clinics for testing…"

**Response:** We thank the reviewer for the comment. We have now amended this statement:

*“offering vaccination to individuals attending ~~to~~ sexual health clinics for testing (vaccination on attendance [VoA]);”*

**Comment:** Change "diagnoses" to "diagnosed" in the following sentence "…offering vaccination to individuals attending to sexual health clinics and were diagnoses with gonorrhea…".

**Response:** We thank the reviewer for the comment. We have now amended this statement:

*“offering vaccination on individuals attending sexual health clinics and were diagnosed with Gonorrhea”*

**Comment:** In vaccination scenarios 2 and 3, the authors discuss offering vaccines to "individuals". Do the authors mean all persons attending a sexual health clinic (SHC) or MSM attending a SHC.

**Response:** We thank the reviewer for the comment. We meant all MSM attending a sexual health clinic:

**Comment:** Does the diagnoses in vaccination scenario 4 represent any diagnoses at any time (ever diagnosed) or diagnoses in a prior time period eg diagnoses in past 12 months.

**Response:** We thank the reviewer for the comment. We meant diagnoses on attendance to a clinic and amended the statement for clarity:

*“or when they are diagnosed with gonorrhea at a clinic (vaccination on diagnosis [VoD)]).”*

**Comment:** Please be clear - In the background section, the authors state that they explored the efficacy of potential GC vaccination in the context of declining GC incidence. In the methods, the authors state that "they employed an integrated transmission dynamic model...in reducing GC transmission in the men who have sex with men population in Singapore" but the results section describes the effect on MSM. Are the authors modeling the impact of vaccination on GC in the general population or MSM OR they vaccinating the general population in the six scenarios highlighted to assess the impact on MSM??? This is not very clear and confusing. The authors should clarify this so that the reader does not have to infer the objective of the paper.

**Response:** We thank the reviewer for the comment. The study population of interest is the MSM population. After the background section, which was intended to provide the overview, we have made clear in the methods, findings and interpretation that our primary focus is the MSM population. Most vaccination programmes are conducted primarily in sexual health clinics for the MSM population (vaccination on attendance [VoA]) or when they are diagnosed with gonorrhea at a clinic (vaccination on diagnosis, vaccination according to risk and vaccination on attendance). However, for adolescent vaccinations (vaccination before entry), this is offered to all male adolescents before entering sexually mature age due to the difficulty of identifying MSMs at the point of time.

*“****Background*** *Gonorrhoea ranks the second most common sexually transmitted infections notified in Singapore in 2023, despite decreasing incidence over the years. Evidence suggests that the four-component serogroup B meningococcal (4CMenB) vaccine designed against Neisseria meningitidis can offer partial cross-protection against gonorrhoea. This has generated interest in using 4CMenB for the purpose of staving gonorrhoea transmission. Here, we explored the efficacy of potential gonorrhoea vaccination strategies in the context of historically declining gonorrhoea incidence.*

***Methods*** *We employed an integrated transmission-dynamic model, calibrated using Bayesian methods to local surveillance data to understand the potential public health impact of 4CMenB in reducing gonorrhea transmission in men who have sex with men (MSM) in Singapore. We explored the efficacy of implementing 6 vaccination programmes, (****1****) offering vaccination to all male adolescents in schools (vaccination before entry [VbE]), (****2****) offering vaccination to individuals attending sexual health clinics for testing (vaccination on attendance [VoA]); (****3****) offering vaccination on individuals attending sexual health clinics and were diagnosed with Gonorrhoea (vaccination on diagnosis [VoD]); or (****4****) vaccination according to risk (VaR), by offering vaccination to patients who were diagnosed with gonorrhoea plus individuals who tested negative but report having more than five sexual partners per year. We further examined how altering (****5****) VoA and (****6****) VoD strategies changed if the strategies only targeted high risk groups (VoA(H), VoD(H)). We assessed efficacy by examining vaccination impact relative to the baseline case of no vaccination and during when behavioural parameters (sexual risk behaviour and screening rates) were held constant. We further ascertained the effects of varying vaccine uptake (10%, 33%, 100%), vaccine efficacy (22%, 31%, 47%) and duration of protection (1.5, 4, 7.5 years) on the effectiveness of each vaccination strategy.*

***Findings*** *In a hypothetical 10-year vaccination programme, VbE had 14.18% of MSM Gonorrhoea cases averted over the intervention time. VoA had the highest protective impact on the MSM population with 40.26% averted cases (95% CrI 18.32%-52.57%) but required more vaccine doses than any other strategy, whereas VoD had a smaller impact (12.04% averted cases (95% CrI 7.12%-15.00%)) but three times efficient higher than that of VoA in terms of averted cases per dose. VoA(H) and VoD(H) improved the efficiency of VoA and VoD strategies by increasing averted cases per dose to 0.22 and 0.24 respectively, but conferred similar protective effects as VoA (VoA(H): 40.10% averted cases (95% CrI 18.14%-52.55%)) and VoD (VoD(H): 12.04% averted cases (95% CrI 7.12%-15.00%)) respectively. VaR (40.10% averted cases (95% CrI 18.14%-52.55%)) had almost the same impact as VoA but required fewer doses administered than VoA, with averted cases per dose at 0.21 (95% CrI: 0.12-0.27). Sensitivity analyses which varied vaccine duration of protection and efficacy indicated that VaR had the greatest public health impact with the highest number of averted cases per dose for vaccines of any efficacy or duration of protection (or both), although VoD and VoD(H) saved more vaccine resource and had the highest number averted MSM cases per dose for highly protective vaccines of long protection.”*

**Comment:** First sentence of the Interpretation. "Vaccination of MSM against gonorrhea…" This is the first reference to MSM without spelling out what it means. The term "men who have sex with men" was first used in the methods but the reference to the abbreviation, MSM, was not made.

**Response:** We thank the reviewer for the comment. We have now added the explanation to the abbreviation at the first mention of MSM, which was found in the methods:

*“We employed an integrated transmission-dynamic model, calibrated using Bayesian methods to local surveillance data to understand the potential public health impact of 4CMenB in reducing gonorrhea transmission in men who have sex with men (MSM) in Singapore”*

**Comment:** Introduction. 1st paragraph line 5. Change "asymptotic" to "asymptomatic".

**Response:** We thank the reviewer for the comment and have amended the typo:

“Among them, most male urogenital infections are symptomatic while the rectal and pharyngeal infections are asymptomatic [4,5]”

**Comment:** 1st paragraph, line 8. Change "urethral" to "urethra".

**Response:** We thank the reviewer for the comment and have now amended this:

*“According to the Department of Sexually Transmitted Infections Control (DSC), Singapore, the majority of MSM gonorrhoea incidence have single site infections at urethra”*

**Comment:** Paragraph 2: Authors should please list the "non-pharmaceutical interventions (NPIs)" that they are referring to. Are they referring to stay-at-home orders, lockdown that may have impacted sexual relationships or STI testing?

**Response:** We thank the reviewer for the comment. It is difficult to distinguish which NPI has impacted gonorrhoea transmission due to concurrent interventions to stave COVID-19 during this period. However, we have expanded this section to introduce the potential impacts that some COVID-19 NPIs have on the disease:

*“While gonorrhoea incidence has moderated in during the COVID-19 pandemic, which may be due to reductions in sexual contacts during lockdowns/stay-at-home orders and/or reduced testing, evidence showed that gonorrhoea transmission is recovering and may be surpassing pre-COVID-19 levels, due to relaxation of non-pharmaceutical measures motivated by COVID-19 [6].”*

**Comment:** Paragraph 3 line 6. Suggest changing "…as of date.,." to "…to date…"

**Response:** We thank the reviewer for the comment. We have amended this section:

*“Since then, the local antibiogram of gonorrhoea cultures has been closely monitored for decreased susceptibility and resistance to the recommended antibiotics, but to date, few resistant strains have been diagnosed in Singapore.”*

**Comment:** Paragraph 3 line 13. The authors state that 4CMenB has a 22% efficacy against GC and cite the Molina et al study (Doxycycline prophylaxis and meningococcal group B vaccine to prevent bacterial sexually transmitted infections in France (ANRS 174 DOXYVAC): a multicentre, open-label, randomised trial with a 2 × 2 factorial design. Lancet ID). However, this study did not detect a significant protective effect (aHR 0·78 [95% CI 0·60-1·01]; p=0·061) against GC for reasons Molina and colleagues clearly outline in their paper, such as a potentially underpowered sample size to detect a small effect size. It is not correct to state that this study suggested "…a more conservative 22% efficacy of MenB-4C vaccine in gonorrhea prevention…" when it was not significantly protective.

**Response:** We thank the reviewer for the comment and have amended this sentence to be accurate:

*“However, a recent study suggested a more conservative, statistically insignificant 22% efficacy of 4CMenB vaccine in gonorrhoea prevention which may be due to an underpowered sample size [7].”*

**Comment:** Last paragraph of introduction: Include a statement that clearly states the objective of this study. The last paragraph includes sentences that contain elements of the objective of their modelling study but it is left to the reader to try and decipher the study's objective.

**Response:** We thank the reviewer for the comment. We have now clearly stated the objective of this study in the last paragraph of the introduction:

“In this study, we aimed to guide potential public health decisions regarding the implementation of potential gonorrhoea vaccination programmes. We sought to devise and compare alternative, pragmatic approaches to implementing vaccination within sexual health clinics or educational institutions, in terms of their public health impact and efficiency. To do so, we used a high-resolution compartmental model of gonorrhoea and utilized a Bayesian evidence synthesis framework to characterize the transmission dynamics of gonorrhoea. This was calibrated using 3 different case time series of gonorrhoea incidence. We examined how the impact of vaccination programs depends on vaccine-specific factors, such as uptake, efficacy, and duration of protection, as well as different epidemic trajectories under consideration.”

**Comment:** First paragraph, line 1: MSM is already abbreviated in the intro (para 1, line 6). There is no need to abbreviate again in the methods. Also, please delete "the" before men who have sex with (MSM) population in Singapore (paragraph 1, line 2).

**Response:** We thank the reviewer for the comment. We have now made the suggested changes:

*“The infection typically affects the mucosal epithelium of ~~the~~ male urogenital tracts, the rectum, pharynx, or conjunctiva, manifesting with inflammation, discharge, and a burning sensation during urination [4].”*

*“****Added value of this study*** *To our knowledge this is the first analysis of gonorrhoea vaccination that accounts for its impact on future rates of infection in declining incidences. Our model showed that offering vaccination against gonorrhoea to all men who have sex with men ~~(MSM)~~”*

*“****Model structure*** *We adapted a previously published compartmental model to characterize gonorrhoea transmission among the men who have sex with men ~~(MSM)~~ population in Singapore [3].”*

**Comment:** Are the 2023 GC incidence estimates of MSM from the communicable surveillance system of Singapore similar to the proxy estimates from 2004-2018 that the authors calculated. I realize male same sex behavior was decriminalized in 2022 in Singapore but it is important to know to determine if the incidence estimate used for this analysis has any bearing on current estimates from 2023.

**Response:** We thank the reviewer for the comment. Unfortunately, we cannot comment on the rates of transmission post- 2020 due to a lack of publicly available data post-2020 on gonorrhoea incidences. We have however validated the predictive ability of our model in the set of available data to show that it can accurately project future gonorrhoea trends with reasonable error rates in the test set data:

*“We further assessed the predictive ability of our models through an expanding cross-validation scheme, where the data was split to initial testing and training sets (70%, 30%), and the model was calibrated to the training set and annual gonorrhoea incidence rates projected forward in time to assess concordance with the test-set observations. In the subsequent cross-validation steps, an additional year of data was added in and the procedure is repeated till the testing set is depleted. Results were inspected visually through whether the model could capture the general trends in annual gonorrhoea incidence rates in the test set, and quantified through mean absolute percentage errors.****”***

***Response Figure:*** *Fitted and predicted values of gonorrhoea incidences from the calibrated model.* *Results were generated through an expanding cross-validation scheme, where the data was split to initial testing and training sets (70%, 30%), and the model was calibrated to the training set and annual gonorrhoea incidence rates projected forward in time to assess concordance with the test-set observations. In the subsequent cross-validation steps, an additional year of data was added in and the procedure is repeated till the testing set is depleted.*

| Final year of training set | Mean absolute percentage error (%) |
| --- | --- |
| 2014 | 41.05% |
| 2015 | 38.45% |
| 2016 | 38.58% |
| 2017 | 12.74% |

***Response Table****: Mean absolute percentage errors of model in out-of-sample prediction of annual gonorrhoea incidences. Results were generated through an expanding cross-validation scheme, where the data was split to initial testing and training sets (70%, 30%), and the model was calibrated to the training set and annual gonorrhoea incidence rates projected forward in time to assess concordance with the test-set observations. In the subsequent cross-validation steps, an additional year of data was added in and the procedure is repeated till the testing set is depleted.*

**Comment:** Vaccination scenario: Paragraph 2: In the VBE scenario, authors state they considered vaccination of male adolescents before they reach sexually active age but do not state what this age. Please include in the paper.

**Response:** We thank the reviewer for the comment. We have added that adolescents are vaccinated at 15-years of age.

*“We considered vaccination of 4CMenB by offering vaccinations to all male adolescents as a nationwide school vaccination programme before they reach sexually active age (15-years-old, vaccination before entry [VbE])”*

**Comment:** VoA Scenario 2: In VoA, authors offer vaccination to MSM when they attend a SHC. How do the authors define MSM in this scenario - men who report ever having sex with a man OR men who had sex with a man within a specified period (such as in the previous 12 months or some other time interval)? Please include this information in the paper.

**Response:** We thank the reviewer for the comment. We defined MSM as men who report ever having sex with a man.

*“The model was calibrated using a Bayesian inference framework using proxies of annual gonorrhoea MSM incidence from 2004 to 2018 in Singapore. MSMs were defined to be men who report ever having sex with a man. As actual breakdowns of annual gonorrhoea MSM incidence were not available in the study setting,”*

**Comment:** Please check the confidence interval for the VbE strategy for the estimate of # of vaccinations administered (Table 4). The # of vaccinations administered, and the upper and lower limit of the confidence intervals are all 172701.

**Response:** We thank the reviewer for the comment. As population census data was used to obtain the number of entrants to the 15-year-old population (and assumed to be the same from 2024 to 2034), there is no uncertainty associated to the number vaccinated according to the VbE strategy. This is in contrast to the other strategies, where there is uncertainty associated to the number of individuals presenting to sexual health clinics due to the inherent uncertainty in epidemiological parameters (asymptomatic rate, number tested etc). We append the Main Text Table 4 below for reference:

| **Strategy** | **Averted cases**  **(total)** | **Averted cases**  **(%)** | **Averted cases**  **per dose** | **Number of vaccinations administered** | **Primary**  **uptake ratio** |
| --- | --- | --- | --- | --- | --- |
| VbE | 1648 (942-2120) | 14.18 (9.11-17.08) | 0.005 (0.003-0.006) | 172701 (172701-172701) | 100.00 (100.00-100.00) |
| VoD(H) | 1427 (551-2176) | 12.04 (7.12-15.00) | 0.24 (0.14-0.31) | 2954 (1674-3912) | 96.79 (95.47-97.69) |
| VoD | 1427 (551-2176) | 12.04 (7.12-15.00) | 0.22 (0.12-0.28) | 3317 (1853-4252) | 97.13 (95.63-97.96) |
| VoA | 4704 (1948-6947) | 40.26 (18.32-52.57) | 0.06 (0.02-0.10) | 45699 (13004-73327) | 91.62 (85.35-95.57) |
| VoA(H) | 4685 (1938-6922) | 40.10 (18.14-52.55) | 0.22 (0.12-0.28) | 11622 (5502-15669) | 87.13 (79.54-92.24) |
| VaR | 4685 (1939-6922) | 40.10 (18.14-52.55) | 0.21 (0.12-0.27) | 11874 (5906-15851) | 87.40 (79.73-92.61) |

***Table 4 Summary of population health impact for various vaccination programmes from 2024 to 2034.*** *We report posterior means and accompanying 95% credible intervals. Estimates presented are relative to the baseline scenario of no intervention, with transmission probabilities from 2024 to 2034 kept stable at 2018 levels.*

**Comment:** In the foot notes of Table 1, the authors state that the calculation for averted cases per dose is computed as the difference between # of treated GC patients between the baselines case and the respective vaccination strategy, divided by the # of vaccination doses. In Table 4, does this mean that the averted cases per dose (column 2) can be calculated as averted cases (column 2) divided by number of vaccinations administered (column 5). If this is interpretation is correct, the current calculation does not result in column 2 using the data in Table 4.

**Response:** We thank the reviewer for the comment. As two doses were administered per primary vaccination uptake, it would require a further division by 2 in order to get the averted cases per dose. I.e, for the case of VbE, where 172701 vaccinations were administered and 1648 cases were adverted, the averted cases per dose would be (1678/(172701*2)). Additionally, revaccination in sexual health clinics contribute one dose per time to number of vaccinations administrated when vaccination strategy applicable, e.g, VoD, VoD(H), VoA, VoA(H), and VaR. We append the mathematical expression in the Supplementary materials 15-17 for reference. We have made the captions for Table 4 clear on this point:

*We assume that primary vaccination required two doses, and that revaccination after protection has waned requires a single booster dose. We assumed that all individuals complete their vaccination regiment. Vaccination in sexual health clinics is offered to unvaccinated MSM and those whose protection has waned, i.e. those in strata* $i \in\left\{ X, W \right\}.$ *We denote the total number of vaccine doses administered in each stratum in year* $t$*as* $V^{i}\left( t \right),$ *where:*

$$\begin{aligned} \begin{aligned} V^{X}\left( t \right)=2\left( Mp^{VbE}+p_{j}^{VoS}Y_{U}^{X}\left( t \right)+p_{j}^{VoD}Y_{D}^{X}\left( t \right) \right)\#(15) \end{aligned} \end{aligned}$$

$$\begin{aligned} \begin{aligned} V^{W}\left( t \right)=p_{j}^{VoS}Y_{U}^{W}\left( t \right)+p_{j}^{VoD}Y_{D}^{W}\left( t \right)\#(16) \end{aligned} \end{aligned}$$

*Therefore, the total number of doses administered in year* $t$ *across the whole population is:*

$$\begin{aligned} \begin{aligned} V\left( t \right)=V^{X}\left( t \right)+V^{W}\left( t \right)\#(17) \end{aligned} \end{aligned}$$

*“****Table 4 Summary of population health impact for various vaccination programmes from 2024 to 2034.*** *We report posterior means and accompanying 95% credible intervals. Estimates presented are relative to the baseline scenario of no intervention, with transmission probabilities from 2024 to 2044 kept stable at 2018 levels.** *Assumes that individuals received two doses for primary uptake, and one dose for revaccination.”*

**Comment:** Vaccine profile. The authors used a vaccine effectiveness of 31% as the primary vaccine profile of 4CMenB vaccine this analysis (Table 2) and cite a study by Petousis-Harris et al. Effectiveness of a group B outer membrane vesicle meningococcal vaccine against gonorrhoea in New Zealand: a retrospective case-control study. Lancet). However, the Petousis-Harris study assessed the effectiveness of MenZB vaccine (a vaccine that is no longer available) against GC and not 4CMenB. Although the outer membrane protein components of 4CMenB are similar to MenZB, 4CMenB contains recombinant proteins that some research shows may provide additional protection over MenZB (Semchenko et al. 2019. The Serogroup B Meningococcal Vaccine Bexsero Elicits Antibodies to Neisseria gonorrhoeae). When Whittles and colleagues conducted their study, I concede that data of 4CMenB were limited but since then there are at least 7 studies on VE estimates of 4CMenB and 2 meta-analytical studies that have published estimated pooled VE estimates.

**Response:** We thank the reviewer for the comment. We understand the uncertainty surrounding the efficacy of the 4CMenB against gonorrhoea. This was the reason why we have explored the use of other ranges of vaccine efficacy at 22%, 47%, following Molina et al 2024 and Robison et al. 2023 respectively. We have now also included the latest pooled VE estimate by Abara et al. 2024 (32.4%) [8] as suggested by the reviewer, for the projections of public health impacts for each vaccination programme. As this was close to the 31% of the primary vaccine profile, we found no major changes to our main result estimates:

**Response Figure : Projected number of annual diagnosed cases by vaccination programme over 2024–2034** **under a vaccine efficacy of 32.4%** The posterior mean is marked with a square, and the 95% credible interval is indicated by the boundaries of the box. Projections assume that Transmission probabilities of 2024 to 2034 were kept constant at 2018 levels. 1000 posterior draws were used to generate future projections and calculate programme-specific metrics. The series of boxplots illustrate the annual future number of diagnosed cases under different vaccination strategies compared to a no-vaccination programme baseline. Each column represents a different vaccination programme. Respectively, these are denoted (**A**) vaccination before entry [VbE], (**B**) vaccination on attendance [VoA], (**C**) vaccination on diagnoses with gonorrhoea [VoD], (**D**) vaccination according to risk [VaR], (**E**) VoA and (**F**) VoD strategies by offering vaccinations in these groups to only high sexual activity individuals (VoA[H]), VoD[H]). The radar graph visualizes the impact, efficiency, and uptake of each vaccination programme by 10-year expectation. Metrics are averted cases by percentage (A.P.), averted cases per dose (A.D.), number of vaccinations administered (N.V.), and primary uptake ratio (P.U.). Each sample's metric value is normalized by the maximum and minimum across strategies for visual clarity, points closer to the center are closer to the overall strategy minimum metric score, and the radar border represents the overall maximum score each strategy can achieve.

**Main Text Figure 2: Projected number of annual diagnosed cases by vaccination programme over 2024–2034** **under a vaccine efficacy of 31%**The posterior mean is marked with a square, and the 95% credible interval is indicated by the boundaries of the box. Projections assume that Transmission probabilities of 2024 to 2034 were kept constant at 2018 levels. 1000 posterior draws were used to generate future projections and calculate programme-specific metrics. The series of boxplots illustrate the annual future number of diagnosed cases under different vaccination strategies compared to a no-vaccination programme baseline. Each column represents a different vaccination programme. Respectively, these are denoted (**A**) vaccination before entry [VbE], (**B**) vaccination on attendance [VoA], (**C**) vaccination on diagnoses with gonorrhoea [VoD], (**D**) vaccination according to risk [VaR], (**E**) VoA and (**F**) VoD strategies by offering vaccinations in these groups to only high sexual activity individuals (VoA[H]), VoD[H]). The radar graph visualizes the impact, efficiency, and uptake of each vaccination programme by 10-year expectation. Metrics are averted cases by percentage (A.P.), averted cases per dose (A.D.), number of vaccinations administered (N.V.), and primary uptake ratio (P.U.). Each sample's metric value is normalized by the maximum and minimum across strategies for visual clarity, points closer to the center are closer to the overall strategy minimum metric score, and the radar border represents the overall maximum score each strategy can achieve.

**Comment:** Results. Para 1.line 4. Suggest changing "infectees" to "infected persons".

**Response:** We thank the reviewer for the comment and have made the suggested amendment:

*“16.85% (95% CrI: 4.75%-27.76%) of treated infected persons are estimated to be asymptomatic and 83.15% (95% CrI: 68.34%-92.50%) symptomatic.”*

**Comment:** How did the authors estimate high risk population among MSM. What parameters were used? Whittles et al. 2022 used data from Genitourinary Medicine Clinic Activity Dataset and the Gonococcal Resistance to Antimicrobials Surveillance Programme. The authors of this paper used data from the communicable disease surveillance system of Singapore. How did the authors estimate the number of MSM to categorize as high-risk MSM (i.e. those that reported 5 or more partners annually) for this study (Table 2). Was this inferred from male or female incidence in the surveillance system? Please explain this similar to the way the authors estimated GC incidence estimate among MSM was explained.

**Response:** We thank the reviewer for the comment. Survey on sexual health behaviours are not publicly available in Singapore. This was why we proxied the proportion of population in low/high risk groups and the threshold for high-risk MSM to be 5 or more partners annually to follow the Whittles et al. 2022 study. Whereas the MSM size was taken as 139,000 following a Singapore specific estimate. The number of high/low risk MSM were therefore computed as 0.15*139,000 or 0.85*139,000 respectively.

As there is uncertainty surrounding the assumed parameters, we have further conducted an additional sensitivity analysis on varying the proportion of individuals in the low/high risk groups, as well as the partner change rate by $\pm$25,50% and found that significantly decreasing the proportion of individuals in the high-risk group reduces the raw public health impact of the strategies, whereas changing the partner change rate in either high/low risk groups had a mixed impact on the efficiency and raw public health impact of the vaccination strategies:

| **Strategy** | **Averted cases**  **(total)** | **Averted cases**  **(%)** | **Averted cases**  **per dose** | **Number of vaccinations administered** | **Primary**  **uptake ratio** |
| --- | --- | --- | --- | --- | --- |
| VbE | 1564 (868-2078) | 13.59 (9.27-16.47) | 0.005 (0.003-0.006) | 172701 (172701-172701) | 100.00 (100.00-100.00) |
| VoD(H) | 1433 (522-2243) | 12.22 (7.19-15.91) | 0.23 (0.13-0.31) | 3099 (1867-3885) | 96.64 (95.32-97.63) |
| VoD | 1434 (522-2243) | 12.22 (7.19-15.91) | 0.22 (0.12-0.30) | 3262 (2009-4059) | 96.80 (95.43-97.76) |
| VoA | 4459 (1725-6665) | 38.55 (17.05-51.47) | 0.06 (0.02-0.10) | 44300 (10341-71584) | 91.82 (86.06-95.58) |
| VoA(H) | 4452 (1720-6649) | 38.48 (17.03-51.38) | 0.21 (0.11-0.28) | 11420 (4680-15676) | 87.36 (80.96-92.57) |
| VaR | 4452 (1720-6649) | 38.48 (17.03-51.38) | 0.21 (0.11-0.27) | 11537 (4891-15805) | 87.48 (81.06-92.69) |

**SI Table Summary of population health impact for various vaccination programmes from 2024 to 2034, with a -50% change in the partner change rate for the low risk group.** We report posterior means and accompanying 95% credible intervals. Estimates presented are relative to the baseline scenario of no intervention, with transmission probabilities from 2024 to 2034 kept stable at 2018 levels.

| **Strategy** | **Averted cases**  **(total)** | **Averted cases**  **(%)** | **Averted cases**  **per dose** | **Number of vaccinations administered** | **Primary**  **uptake ratio** |
| --- | --- | --- | --- | --- | --- |
| VbE | 1594 (850-2105) | 13.72 (8.78-16.81) | 0.005 (0.002-0.006) | 172701 (172701-172701) | 100.00 (100.00-100.00) |
| VoD(H) | 1429 (451-2230) | 12.05 (7.05-15.26) | 0.24 (0.13-0.31) | 3043 (1563-3916) | 96.70 (95.13-97.64) |
| VoD | 1429 (451-2231) | 12.05 (7.05-15.26) | 0.22 (0.12-0.29) | 3307 (1754-4323) | 96.95 (95.45-97.86) |
| VoA | 4574 (1994-6784) | 39.15 (19.78-50.80) | 0.05 (0.02-0.09) | 46346 (13989-78220) | 91.59 (85.06-95.08) |
| VoA(H) | 4561 (1983-6766) | 39.04 (19.68-50.69) | 0.21 (0.11-0.28) | 11534 (5977-15607) | 87.23 (81.14-91.65) |
| VaR | 4561 (1983-6766) | 39.04 (19.69-50.69) | 0.21 (0.11-0.27) | 11720 (6374-15859) | 87.43 (81.29-91.93) |

**SI Table Summary of population health impact for various vaccination programmes from 2024 to 2034, with a -25% change in the partner change rate for the low risk group.** We report posterior means and accompanying 95% credible intervals. Estimates presented are relative to the baseline scenario of no intervention, with transmission probabilities from 2024 to 2034 kept stable at 2018 levels.

| **Strategy** | **Averted cases**  **(total)** | **Averted cases**  **(%)** | **Averted cases**  **per dose** | **Number of vaccinations administered** | **Primary**  **uptake ratio** |
| --- | --- | --- | --- | --- | --- |
| VbE | 1556 (788-2118) | 13.47 (8.82-16.76) | 0.005 (0.002-0.006) | 172701 (172701-172701) | 100.00 (100.00-100.00) |
| VoD(H) | 1314 (374-2047) | 11.13 (5.90-14.39) | 0.23 (0.12-0.31) | 2893 (1591-3858) | 96.86 (95.23-97.82) |
| VoD | 1315 (374-2048) | 11.14 (5.90-14.39) | 0.20 (0.10-0.26) | 3334 (1873-4381) | 97.25 (95.36-98.08) |
| VoA | 4468 (1675-6634) | 38.44 (19.36-50.46) | 0.05 (0.02-0.09) | 47296 (13573-77785) | 91.44 (85.15-95.25) |
| VoA(H) | 4441 (1666-6618) | 38.21 (19.17-50.16) | 0.20 (0.10-0.27) | 11664 (5055-15768) | 87.08 (80.87-91.96) |
| VaR | 4442 (1666-6618) | 38.22 (19.18-50.16) | 0.20 (0.10-0.26) | 11975 (5513-15934) | 87.41 (81.15-92.26) |

**SI Table Summary of population health impact for various vaccination programmes from 2024 to 2034, with a +25% change in the partner change rate for the low risk group.** We report posterior means and accompanying 95% credible intervals. Estimates presented are relative to the baseline scenario of no intervention, with transmission probabilities from 2024 to 2034 kept stable at 2018 levels.

| **Strategy** | **Averted cases**  **(total)** | **Averted cases**  **(%)** | **Averted cases**  **per dose** | **Number of vaccinations administered** | **Primary**  **uptake ratio** |
| --- | --- | --- | --- | --- | --- |
| VbE | 1680 (1004-2266) | 14.08 (9.63-17.47) | 0.005 (0.003-0.007) | 172701 (172701-172701) | 100.00 (100.00-100.00) |
| VoD(H) | 1401 (548-2140) | 11.53 (7.39-14.88) | 0.25 (0.15-0.33) | 2873 (1623-3770) | 96.88 (95.29-97.80) |
| VoD | 1402 (548-2141) | 11.54 (7.40-14.88) | 0.20 (0.12-0.28) | 3431 (1942-4401) | 97.36 (95.73-98.18) |
| VoA | 4633 (1926-6819) | 38.60 (18.53-51.42) | 0.06 (0.03-0.10) | 44035 (12515-74755) | 91.89 (85.36-95.75) |
| VoA(H) | 4598 (1917-6772) | 38.31 (18.30-51.16) | 0.22 (0.12-0.29) | 11264 (4875-15447) | 87.53 (81.77-92.81) |
| VaR | 4599 (1918-6773) | 38.32 (18.31-51.17) | 0.21 (0.12-0.27) | 11660 (5511-15773) | 87.95 (82.12-93.23) |

**SI Table Summary of population health impact for various vaccination programmes from 2024 to 2034, with a +50% change in the partner change rate for the low risk group.** We report posterior means and accompanying 95% credible intervals. Estimates presented are relative to the baseline scenario of no intervention, with transmission probabilities from 2024 to 2034 kept stable at 2018 levels.

| **Strategy** | **Averted cases**  **(total)** | **Averted cases**  **(%)** | **Averted cases**  **per dose** | **Number of vaccinations administered** | **Primary**  **uptake ratio** |
| --- | --- | --- | --- | --- | --- |
| VbE | 1706 (1345-1960) | 14.63 (11.21-16.60) | 0.005 (0.004-0.006) | 172701 (172701-172701) | 100.00 (100.00-100.00) |
| VoD(H) | 1592 (945-2060) | 13.54 (10.84-15.47) | 0.25 (0.20-0.29) | 3173 (2271-3767) | 96.56 (95.63-97.20) |
| VoD | 1592 (945-2060) | 13.54 (10.84-15.47) | 0.25 (0.19-0.29) | 3258 (2302-3850) | 96.65 (95.72-97.27) |
| VoA | 5641 (3124-7177) | 48.25 (27.63-58.40) | 0.07 (0.04-0.13) | 48046 (13980-80971) | 90.33 (83.75-93.81) |
| VoA(H) | 5638 (3122-7174) | 48.22 (27.59-58.40) | 0.22 (0.15-0.26) | 14090 (7223-18309) | 84.34 (78.05-89.29) |
| VaR | 5638 (3122-7174) | 48.22 (27.59-58.40) | 0.22 (0.15-0.25) | 14143 (7321-18327) | 84.40 (78.06-89.41) |

**SI Table Summary of population health impact for various vaccination programmes from 2024 to 2034, with a -50% change in the partner change rate for the high risk group.** We report posterior means and accompanying 95% credible intervals. Estimates presented are relative to the baseline scenario of no intervention, with transmission probabilities from 2024 to 2034 kept stable at 2018 levels.

| **Strategy** | **Averted cases**  **(total)** | **Averted cases**  **(%)** | **Averted cases**  **per dose** | **Number of vaccinations administered** | **Primary**  **uptake ratio** |
| --- | --- | --- | --- | --- | --- |
| VbE | 1991 (1243-2562) | 14.64 (10.32-17.50) | 0.006 (0.004-0.007) | 172701 (172701-172701) | 100.00 (100.00-100.00) |
| VoD(H) | 1821 (804-2854) | 13.17 (8.74-16.40) | 0.29 (0.18-0.38) | 3149 (2132-3973) | 96.58 (94.96-97.41) |
| VoD | 1823 (805-2858) | 13.18 (8.75-16.40) | 0.24 (0.15-0.31) | 3831 (2583-4875) | 97.16 (95.58-97.90) |
| VoA | 5876 (2426-8461) | 42.96 (19.71-54.57) | 0.08 (0.03-0.15) | 43695 (12050-73717) | 91.39 (84.32-94.86) |
| VoA(H) | 5837 (2407-8413) | 42.68 (19.61-54.29) | 0.25 (0.16-0.32) | 12457 (5599-16520) | 86.19 (79.18-91.09) |
| VaR | 5838 (2408-8413) | 42.69 (19.62-54.29) | 0.24 (0.15-0.31) | 12916 (6221-16938) | 86.67 (79.57-91.74) |

**SI Table Summary of population health impact for various vaccination programmes from 2024 to 2034, with a -25% change in the partner change rate for the high risk group.** We report posterior means and accompanying 95% credible intervals. Estimates presented are relative to the baseline scenario of no intervention, with transmission probabilities from 2024 to 2034 kept stable at 2018 levels.

| **Strategy** | **Averted cases**  **(total)** | **Averted cases**  **(%)** | **Averted cases**  **per dose** | **Number of vaccinations administered** | **Primary**  **uptake ratio** |
| --- | --- | --- | --- | --- | --- |
| VbE | 1659 (1136-2031) | 14.31 (10.75-16.79) | 0.005 (0.003-0.006) | 172701 (172701-172701) | 100.00 (100.00-100.00) |
| VoD(H) | 1453 (655-2166) | 12.31 (8.85-14.82) | 0.24 (0.17-0.30) | 2988 (1846-3863) | 96.76 (95.37-97.61) |
| VoD | 1454 (655-2166) | 12.32 (8.85-14.82) | 0.22 (0.15-0.28) | 3301 (2059-4279) | 97.05 (95.59-97.85) |
| VoA | 4763 (2103-6885) | 40.80 (20.86-54.58) | 0.07 (0.03-0.12) | 40542 (12940-68963) | 92.07 (86.88-95.40) |
| VoA(H) | 4749 (2095-6848) | 40.68 (20.82-54.58) | 0.22 (0.15-0.27) | 11592 (5363-16081) | 87.16 (80.44-92.26) |
| VaR | 4749 (2095-6848) | 40.68 (20.82-54.58) | 0.21 (0.15-0.26) | 11807 (5615-16233) | 87.39 (80.69-92.52) |

**SI Table Summary of population health impact for various vaccination programmes from 2024 to 2034, with a +25% change in the partner change rate for the high risk group.** We report posterior means and accompanying 95% credible intervals. Estimates presented are relative to the baseline scenario of no intervention, with transmission probabilities from 2024 to 2034 kept stable at 2018 levels.

| **Strategy** | **Averted cases**  **(total)** | **Averted cases**  **(%)** | **Averted cases**  **per dose** | **Number of vaccinations administered** | **Primary**  **uptake ratio** |
| --- | --- | --- | --- | --- | --- |
| VbE | 1667 (1002-2128) | 14.42 (10.69-17.02) | 0.005 (0.003-0.006) | 172701 (172701-172701) | 100.00 (100.00-100.00) |
| VoD(H) | 1487 (472-2282) | 12.59 (8.45-15.44) | 0.25 (0.16-0.32) | 3026 (1549-3949) | 96.71 (95.24-97.67) |
| VoD | 1487 (472-2282) | 12.59 (8.45-15.44) | 0.23 (0.14-0.29) | 3282 (1658-4235) | 96.96 (95.38-97.90) |
| VoA | 4638 (1638-6681) | 39.79 (14.87-51.68) | 0.07 (0.02-0.15) | 37545 (6281-65997) | 92.39 (85.68-95.72) |
| VoA(H) | 4628 (1638-6673) | 39.71 (14.86-51.64) | 0.22 (0.12-0.28) | 11111 (3733-15251) | 87.70 (81.03-92.62) |
| VaR | 4628 (1638-6673) | 39.71 (14.86-51.64) | 0.22 (0.12-0.28) | 11292 (3884-15407) | 87.90 (81.17-92.93) |

**SI Table Summary of population health impact for various vaccination programmes from 2024 to 2034, with a +50% change in the partner change rate for the high risk group.** We report posterior means and accompanying 95% credible intervals. Estimates presented are relative to the baseline scenario of no intervention, with transmission probabilities from 2024 to 2034 kept stable at 2018 levels.

| **Strategy** | **Averted cases**  **(total)** | **Averted cases**  **(%)** | **Averted cases**  **per dose** | **Number of vaccinations administered** | **Primary**  **uptake ratio** |
| --- | --- | --- | --- | --- | --- |
| VbE | 1390 (666-1868) | 12.65 (7.56-16.03) | 0.004 (0.002-0.005) | 172701 (172701-172701) | 100.00 (100.00-100.00) |
| VoD(H) | 1535 (427-2398) | 13.64 (8.08-17.27) | 0.30 (0.16-0.41) | 2549 (1301-3458) | 95.84 (93.42-97.08) |
| VoD | 1535 (427-2399) | 13.65 (8.08-17.28) | 0.25 (0.14-0.33) | 3066 (1438-4024) | 96.50 (93.84-97.68) |
| VoA | 4437 (1830-6497) | 40.12 (19.36-52.67) | 0.05 (0.02-0.10) | 48233 (10417-78854) | 91.29 (84.67-95.00) |
| VoA(H) | 4407 (1820-6459) | 39.85 (19.20-52.38) | 0.28 (0.13-0.37) | 8616 (3918-11252) | 85.66 (79.36-90.50) |
| VaR | 4407 (1821-6460) | 39.85 (19.21-52.38) | 0.26 (0.13-0.34) | 8980 (4653-11530) | 86.23 (80.11-91.32) |

**SI Table Summary of population health impact for various vaccination programmes from 2024 to 2034, with the proportion of individuals in the high risk group changed to 0.1.** We report posterior means and accompanying 95% credible intervals. Estimates presented are relative to the baseline scenario of no intervention, with transmission probabilities from 2024 to 2034 kept stable at 2018 levels.

| **Strategy** | **Averted cases**  **(total)** | **Averted cases**  **(%)** | **Averted cases**  **per dose** | **Number of vaccinations administered** | **Primary**  **uptake ratio** |
| --- | --- | --- | --- | --- | --- |
| VbE | 1847 (1133-2311) | 15.00 (10.86-17.59) | 0.005 (0.003-0.007) | 172701 (172701-172701) | 100.00 (100.00-100.00) |
| VoD(H) | 1345 (620-2058) | 10.75 (7.01-13.20) | 0.21 (0.13-0.26) | 3286 (2175-4199) | 97.33 (96.05-98.00) |
| VoD | 1346 (620-2059) | 10.75 (7.01-13.20) | 0.19 (0.12-0.24) | 3572 (2343-4503) | 97.53 (96.24-98.19) |
| VoA | 5104 (2085-7435) | 41.20 (19.83-53.53) | 0.06 (0.03-0.10) | 47314 (14913-74539) | 91.24 (85.42-95.01) |
| VoA(H) | 5089 (2077-7429) | 41.08 (19.81-53.50) | 0.18 (0.11-0.23) | 15214 (7096-20709) | 87.37 (81.39-92.45) |
| VaR | 5089 (2077-7429) | 41.08 (19.81-53.50) | 0.18 (0.11-0.22) | 15406 (7264-20812) | 87.52 (81.58-92.61) |

**SI Table Summary of population health impact for various vaccination programmes from 2024 to 2034, with the proportion of individuals in the high risk group changed to 0.2.** We report posterior means and accompanying 95% credible intervals. Estimates presented are relative to the baseline scenario of no intervention, with transmission probabilities from 2024 to 2034 kept stable at 2018 levels.

| **Strategy** | **Averted cases**  **(total)** | **Averted cases**  **(%)** | **Averted cases**  **per dose** | **Number of vaccinations administered** | **Primary**  **uptake ratio** |
| --- | --- | --- | --- | --- | --- |
| VbE | 2159 (1391-2765) | 15.39 (11.38-18.32) | 0.006 (0.004-0.008) | 172701 (172701-172701) | 100.00 (100.00-100.00) |
| VoD(H) | 1279 (524-2009) | 8.95 (5.98-11.11) | 0.16 (0.10-0.20) | 3984 (2248-5181) | 97.84 (96.78-98.42) |
| VoD | 1280 (524-2010) | 8.95 (5.98-11.11) | 0.15 (0.10-0.20) | 4172 (2369-5402) | 97.94 (96.81-98.50) |
| VoA | 5046 (1632-7700) | 35.74 (12.91-50.71) | 0.07 (0.03-0.11) | 39002 (8389-67793) | 92.32 (85.82-96.14) |
| VoA(H) | 5038 (1631-7693) | 35.68 (12.90-50.57) | 0.14 (0.08-0.18) | 18821 (5460-28216) | 89.61 (82.98-94.61) |
| VaR | 5039 (1631-7693) | 35.68 (12.90-50.57) | 0.14 (0.08-0.18) | 18956 (5587-28372) | 89.69 (83.08-94.71) |

**SI Table Summary of population health impact for various vaccination programmes from 2024 to 2034, with the proportion of individuals in the high risk group changed to 0.3.** We report posterior means and accompanying 95% credible intervals. Estimates presented are relative to the baseline scenario of no intervention, with transmission probabilities from 2024 to 2034 kept stable at 2018 levels.

**Comment:** Although describing the model is important, I would argue that the accuracy of input parameters are even more important as they inform the accuracy and interpretation of the findings, especially as data on MSM in Singapore is lacking.

**Response:** We thank the reviewer for the comment, and agree that the input parameters are extremely important for the work on projections of each vaccination programme. For both assumed and estimated parameters, we have now conducted extensive sensitivity analysis and decomposed the uncertainty in our results to further check the influence of these parameters on our final findings. As these included over 200 different scenarios, we urge the reviewer to refer to the updated supplementary information section for these results

*“We separately calibrated the transmission models based on the different proxies of MSM gonorrhea incidence (see* ***Data*** *above). We projected the future incidences based on constant or linearly trending transmission rates over 2024 – 2034 and assessed the influence of these separate models on vaccination programmes’ public health impacts over the projected time horizon. Furthermore, in order to ascertain how the uncertainty in different parameters contribute to the overall uncertainty of the results, we iteratively set each parameter by its median values and all other parameters to vary according to the sampled distribution. Thereafter each the vaccination programmes’s impacts over a 10-30year timeframe was projected, under the 3 separate case series.”*

*“Lastly, we examined the sensitivity of behavioural and epidemiological parameters on our model’s outputs of the prospective vaccination strategies efficiency and raw public health impacts. This was done by varying the parameters (i.e, screening rates, recovery rates, asymptomatic rates, proportion of individuals in high/low risk groups) by* $\pm$*25,50% individually while keeping all other parameters fixed where possible and reprojecting each vaccination strategy through 2024–2034.”*

**Comment:** I realize that the authors used a cut-off of 5 partners annually based on prior work by Whittles, 2022. However, did the authors consider other more proximal factors that may be suggestive of elevated sexual risk such as condomless anal sex with a casual partner, previous STD in past 12 months, HIV PrEP use (due (due to potential risk compensation behaviors), chemsex, or substance use history. Five sex partners in past 1 year by itself without context such as whether these are concurrent multiple partners or partners with whom they engaged in condomless anal sex does not necessarily speak to risk. MSM who report 5 main, non-concurrent, sex partners who are non-concurrent in the past year may in fact be at lower sexual risk than MSM who report 4 partners (1 main partner with three concurrent casual partners) in the past year. This is something to think about.

**Response:** We thank the reviewer for the comment. Proximal factors which may potentially impact transmission of disease were not taken into account due to a lack of data of that granularity. Additional sensitivity analyses also indicated that the partner change rate does not significantly alter our final results on each vaccination strategy’s impact and efficiency. However, the force of infection of the model is calibrated to historical gonorrhoea incidences and represented the force-of-infection in these groups, averaged across these proximal factors. We have discussed this limitation in the discussion section:

*“Our model only incorporated high/low risk sexual activity groups, but assumed homogeneity in behaviours within each group. Proximal factors, such as past sexual history, unprotected sexual behaviour or substance use were also not accounted for due to lack of available granular data. Therefore, estimation of the force of infection were coarsened to either of high/low risk population subgroups. Potentially important population subgroups, such as superspreaders, were not accounted for. Higher efficiency vaccination strategies, such as targeting those at very high risk, were therefore not explored, as parameters for these population subgroups were not available for model calibration and scenario analysis.”*

**Comment:** Discussion. Para 1 line 3. Authors state "…strategies which targeted individuals on diagnoses…" I suspect that the authors mean MSM. I urge the authors to stick to MSM rather than using population or individuals when they in fact mean MSM. It is confusing and a bit hard to follow.

**Response:** We thank the reviewer for the comment. We meant MSM and amended this sentence to be specific:

*“We calibrated a transmission model of gonorrhoea to explore the population health impacts of potential various vaccination strategies over a 10-year period. Over 2024 – 2034, we found that strategies which targeted MSM on diagnoses (VoD, VoD(H)) were highly efficient in terms of number of averted cases per dose, but had less population health impact, compared to vaccination of MSM on attendance (VoA), in terms of the total number of averted cases.”*

**Comment:** para 2:Do the authors think that the reduced efficiency of vaccination in VoA and VaR compared with VoD should play a role in informing vaccination recommendation strategies. In other words, does the efficiency of a vaccination strategy supersede the proportion of averted cases or vice versa?

**Response:** We thank the reviewer for the comment. The implementation of potential public health programmes invariably comes with efficiency and raw impact trade-offs. It is a practical question which would need to be affirmatively judged together (**1**) when there is a higher certainty of the gonorrhoea vaccine profile (**2**) when more granular epidemiological and costing data becomes available and (**3**) when the prospective effectiveness and cost-effectiveness of alternative strategies (antibiotics) in different future trajectories of antibiotic resistance becomes known.

Under our modelling framework, vaccination according to risk and vaccination according to attendance only leads to a minor decrease in efficiency which does not exceed 10% (in terms of averted cases per dose) but potentially can increase the number of averted cases 3-fold. This may be the recommended strategy which trades-off a small decline in efficiency for extensive public health impact (See main text Table 4 below):

| **Strategy** | **Averted cases**  **(total)** | **Averted cases**  **(%)** | **Averted cases**  **per dose** | **Number of vaccinations administered** | **Primary**  **uptake ratio** |
| --- | --- | --- | --- | --- | --- |
| VbE | 1648 (942-2120) | 14.18 (9.11-17.08) | 0.005 (0.003-0.006) | 172701 (172701-172701) | 100.00 (100.00-100.00) |
| VoD(H) | 1427 (551-2176) | 12.04 (7.12-15.00) | 0.24 (0.14-0.31) | 2954 (1674-3912) | 96.79 (95.47-97.69) |
| VoD | 1427 (551-2176) | 12.04 (7.12-15.00) | 0.22 (0.12-0.28) | 3317 (1853-4252) | 97.13 (95.63-97.96) |
| VoA | 4704 (1948-6947) | 40.26 (18.32-52.57) | 0.06 (0.02-0.10) | 45699 (13004-73327) | 91.62 (85.35-95.57) |
| VoA(H) | 4685 (1938-6922) | 40.10 (18.14-52.55) | 0.22 (0.12-0.28) | 11622 (5502-15669) | 87.13 (79.54-92.24) |
| VaR | 4685 (1939-6922) | 40.10 (18.14-52.55) | 0.21 (0.12-0.27) | 11874 (5906-15851) | 87.40 (79.73-92.61) |

**Table 4 Summary of population health impact for various vaccination programmes from 2024 to 2034.** We report posterior means and accompanying 95% credible intervals. Estimates presented are relative to the baseline scenario of no intervention, with transmission probabilities from 2024 to 2034 kept stable at 2018 levels.

**Comment:** General comment: The paper may require a minor bit of editing as there are some typos in the paper.

**Response:** We thank the reviewer for the comment. We have done an extensive check on the typos in the paper and have rectified them where possible. As these changes appear throughout the manuscript, we have precluded quoting them in the response document.

**Reviewer 3**

**Comment:** There is limited information regarding the justification of some key parameters of compartment model, particularly those related to the mixing between high- and low-risk groups. While the model uses an assortativity parameter, it would be useful to provide sensitivity analyses for varying (ϵ/ epsilon) values. This could highlight how different mixing assumptions affect transmission dynamics.

**Responses:** We thank the reviewer for the comments and note that the comments below crucially highlight the importance of conducting sensitivity analyses for various epidemiological parameters and specification of the transmission model. Primarily, we have ran the sensitivity analyses where it is possible and realistic to conduct. As over 200 scenarios of sensitivity analyses were required, we have provided the output for the efficacy and efficiency of the vaccination programmes for each of these sensitivity analyses, or the number of individuals per compartment when request, as they are the main output of our study. We hope that our responses provide clarity and cogency to our study.

We agree with the reviewer that a change in assortativity may issue transmission dynamics. We have the estimated assortativity parameter by $\pm25,50\%$and found that higher levels of assortativity leads to an increase in the efficacy of vaccination programmes (i.e VoA, VoA(H), VaR) over a 20 year time frame. We have included the exploration of this and the other suggested sensitivity analysis in the main text and the key results in the SI:

*“We separately calibrated the transmission models based on the different proxies of MSM gonorrhea incidence (see* ***Data*** *above). We projected the future incidences based on constant or linearly trending transmission rates over 2024 – 2034 and assessed the influence of these separate models on vaccination programmes’ public health impacts over the projected time horizon. Furthermore, in order to ascertain how the uncertainty in different parameters contribute to the overall uncertainty of the results, we iteratively set each parameter by its median values and all other parameters to vary according to the sampled distribution. Thereafter each the vaccination programmes’s impacts over a 10-30year timeframe was projected, under the 3 separate case series.*

*We further assessed the predictive ability of our models through an expanding cross-validation scheme, where the data was split to initial testing and training sets (70%, 30%), and the model was calibrated to the training set and annual gonnorhoea incidence rates projected forward in time to assess concordance with the test-set observations. In the subsequent cross-validation steps, an additional year of data was added in and the procedure is repeated till the testing set is depleted. Results were inspected visually through whether the model could capture the general trends in annual gonorrhoea incidence rates in the test set, and quantified through mean absolute percentage errors.*

*Lastly, we examined the sensitivity of behavioural and epidemiological parameters on our model’s outputs of the prospective vaccination strategies efficiency and raw public health impacts. This was done by varying the parameters (i.e, screening rates, recovery rates, asymptomatic rates, proportion of individuals in high/low risk groups) by* $\pm$*25,50% individually while keeping all other parameters fixed where possible and reprojecting each vaccination strategy through 2024–2034.”*

**Response figures below describe the projected number of annual diagnosed cases by vaccination programme over 2024–2044% by a -50%,-25%,+25% and +25% change in the estimated assortativity parameter:** The posterior mean is marked with a square, and the 95% credible interval is indicated by the boundaries of the box. Projections assume that transmission probabilities of 2024 to 2054 were kept constant at 2018 levels. 1000 posterior draws were used to generate future projections and calculate programme-specific metrics. The series of boxplots illustrate the annual future number of diagnosed cases under different vaccination strategies compared to a no-vaccination programme baseline. Each column represents a different vaccination programme. Respectively, these are denoted (**A**) vaccination before entry [VbE], (**B**) vaccination on attendance [VoA], (**C**) vaccination on diagnoses with gonorrhoea [VoD], (**D**) vaccination according to risk [VaR], (**E**) VoA and (**F**) VoD strategies by offering vaccinations in these groups to only high sexual activity individuals (VoA[H]), VoD[H]). The radar graph visualizes the impact, efficiency, and uptake of each vaccination programme by 10-year expectation. Metrics are averted cases by percentage (A.P.), averted cases per dose (A.D.), number of vaccinations administered (N.V.), and primary uptake ratio (P.U.). Each sample's metric value is normalized by the maximum and minimum across strategies for visual clarity, points closer to the center are closer to the overall strategy minimum metric score, and the radar border represents the overall maximum score each strategy can achieve.

**-50% change in assortativity** **parameter**

**-25% change in assortativity** **parameter**

**+25% change in assortativity** **parameter**

**+50% change in assortativity** **parameter**

**Comment:** The rate of transmission (β/beta) is modelled as a linear function over time (Equation 1 in the supplementary). However, real-world transmission dynamics are likely to be more complex. Have other functional forms (e.g., exponential decay) been tested? It would strengthen the paper to discuss why a linear trend was chosen and whether alternative forms were explored during model calibration.

**Responses:** We thank the reviewer for the comment. We have tested an exponentially decaying functional form for the rate of transmission:

$$\left( \beta_{t_{0}}+t*\exp(-(\beta_{t_{1}}/\beta_{t_{0}})/ \left( t_{1}-t_{0} \right)) \right)$$

in contrast to the linear trend:

$$\left( \beta_{t_{0}}+t\frac{\left( \beta_{t_{1}}-\beta_{t_{0}} \right)}{\left( t_{1}-t_{0} \right)} \right)$$

and refitted the model using the same procedure as the main results on annual incidence of Gonorrhoea diagnoses. We found that the exponentially decaying functional form for rate of transmission did not characterise observed historical trends well**.** We have now also further described why the linear functional form (and the constant trend) were chosen in the supplementary information section, and also supplemented the main text and supplementary information section with the additional exploration of exponentially decaying and constant rates of transmission to fit annual incidence of Gonorrhoea diagnoses:

*“The force of infection, i.e. the rate of acquisition of infection for an uninfected individual, in sexual activity group* $j$*,* $\lambda_{j} \left( t \right)$*, depends on the rate of partner change per year in each sexual activity group (*$c_{j}$*); the level of assortativity in sexual mixing between groups (*$\epsilon$*, where* $\epsilon$ *denotes proportionate mixing and* $\epsilon=1$ *denotes fully assortative contact [9]; the prevalence of infectees in each group (*$C_{j}\frac{t}{N_{j}}\left( t \right)$*); and the rate of transmission, which we allow to change linearly over time from the* $\beta$ *at* $t_{0}$ *= 2004, to last calibrated data year β at* $t_{1}$*=2018. A linear trend was used as it could characterise the historical changes in gonorrhoea incidence well (See Fig S3 below), in comparison to an exponentially decaying functional form.”*

*“****6. Sensitivity analysis of using other functional forms to fit annual incidence of Gonorrhoea diagnoses***

*We have explored using an exponentially decaying functional form for transmissibility, such that the force of infection is calculated as follows:*

$$\begin{aligned} \lambda_{j}\left( t \right)=c_{j}\left( \beta_{t_{0}}+t\exp(-(\beta_{t_{1}}/\beta_{t_{0}})/ \left( t_{1}-t_{0} \right)) \right)\left( \epsilon\frac{C_{j}\left( t \right)}{N_{j}\left( t \right)}+\left( 1-\epsilon\right)\left( \sum_{i\in\left\{ L,H \right\}} \pi_{i}\left( t \right)\frac{C_{i}\left( t \right)}{N_{i}\left( t \right)} \right) \right)\#\left( 24 \right) \end{aligned}$$

*and refitted the model using the same procedure as the main results on annual incidence of Gonorrhoea diagnoses. We found that the exponentially decaying functional form for rate of transmission did not characterise observed historical patterns well and did not use this for further evaluation of vaccination programmes post-2020.*

***Figure S9:*** ***Comparison of gonorrhoea annual diagnosed projection from calibrated model and observation under an exponentially decaying rate of transmission.*** *The mean of each box is marked as a line, and the 95% credible interval is indicated by the boundaries of the box. Simulated epidemic trajectories (based on a sample of 1,000 parameter sets from the joint posterior) are in boxes, and annual gonorrhoea incidence of Singapore MSM are in squares.”*

**Comment:**    The use of Markov chain Monte Carlo (MCMC) for model calibration is appropriate, but the description of the convergence diagnostics could be more detailed. Specifically, did the authors assess convergence using statistical tests (e.g., Gelman-Rubin diagnostics) or rely solely on visual inspection of trace plots? It would be helpful to provide quantitative convergence statistics in the supplementary material to support the claim of robust calibration.

**Responses:** We thank the reviewer for the comment. The Gelman-Rubin statistic and effective sample size were used to support the claim of robust calibration previously, and were reported together with the trace plots. We append the description from the main text and results in the supplementary material below:

*“Convergence was assessed using visual inspection of trace plots, the Gelman-Rubin statistic, and the effective sample size.”*

**(A)**

**(B)**

**(C)**

**Figure S2: Trace plots of posterior parameter estimates under different observation scenarios.** Trace plots shows the MCMC posterior parameter estimates from eight chains, each with 500,000 iterations. Colored lines represent individual chains. Effective sample size (ESS) and Gelman-Rubin (GR) diagnostics accompany each plot to quantify convergence. The acceptance rate is printed with posterior traces. The first 100,000 iterations for each chain were discarded as burn-in. Model fitted to different observation scenarios, namely, the (**1**) male incidence less the female incidence of gonorrhoea (**2**) the male incidence of gonorrhoea, (**3**) the male incidence multiplied by the proportion of reported gonorrhoea cases in the MSM population in the UK setting.

**Comment:** While the model is calibrated to historical gonorrhoea incidence in Singapore, it is not clear whether any model validation was performed. The authors could strengthen their argument by splitting the dataset into calibration and validation sets, and then testing how well the calibrated model predicts the validation data.

**Responses:** We thank the reviewer for the comment. We have now a conducted model validation by using a block-cross validation scheme. That is, we split the data to initial testing and training sets (70%, 30%), calibrated the model on the train set and projected forward in time, the annual gonorrhoea incidence rates in the test set. We then moved forward in each cross-validation step by adding one more year of training data into the train set and recalibrated the model. This was done until the remaining data in the test set is depleted. We visualized the differences in observed and projected incidences, as well as error rates in terms of the mean absolute percentage error (MAPE). In general, the model could capture the overall decline in incidences well with low MAPE values:

*“We further assessed the predictive ability of our models through an expanding cross-validation scheme, where the data was split to initial testing and training sets (70%, 30%), and the model was calibrated to the training set and annual gonnorhoea incidence rates projected forward in time to assess concordance with the test-set observations. In the subsequent cross-validation steps, an additional year of data was added in and the procedure is repeated till the testing set is depleted. Results were inspected visually through whether the model could capture the general trends in annual gonorrhoea incidence rates in the test set, and quantified through mean absolute percentage errors.****”***

***Response Figure:*** *Fitted and predicted values of gonorrhoea incidences from the calibrated model.* *Results were generated through an expanding cross-validation scheme, where the data was split to initial testing and training sets (70%, 30%), and the model was calibrated to the training set and annual gonorrhoea incidence rates projected forward in time to assess concordance with the test-set observations. In the subsequent cross-validation steps, an additional year of data was added in and the procedure is repeated till the testing set is depleted.*

| Final year of training set | Mean absolute percentage error (%) |
| --- | --- |
| 2014 | 41.05% |
| 2015 | 38.45% |
| 2016 | 38.58% |
| 2017 | 12.74% |

***Response Table****: Mean absolute percentage errors of model in out-of-sample prediction of annual gonorrhoea incidences. Results were generated through an expanding cross-validation scheme, where the data was split to initial testing and training sets (70%, 30%), and the model was calibrated to the training set and annual gonorrhoea incidence rates projected forward in time to assess concordance with the test-set observations. In the subsequent cross-validation steps, an additional year of data was added in and the procedure is repeated till the testing set is depleted.*

**Comment:** The sensitivity analyses cover a good range of vaccine uptake (10%, 33%, 100%) and efficacy (22%, 31%, 47%) parameters. However, it would be useful to explore interactions between vaccine efficacy and duration of protection. For instance, does increasing efficacy reduce the importance of long-duration vaccines, or is there a synergistic effect between high efficacy and long duration?

**Responses:** We thank the reviewer for the comment. We have visualized the changes in efficacy, uptake and duration of protection in the Main Text Figure 4. The changes in impact can be compared on a vaccination strategy-specific basis comparing duration of 1.5, 4, 7.5yrs versus low/medium and high efficacies (mild, normal and strong) by the columns. Higher durations of protection increased averted cases by percentage (as averted cases per dose remained similar), but vaccine efficacy impacted averted cases by percentage and averted cases per dose as they both changed as vaccine efficacy increased. For jointly higher durations of protection and higher vaccine efficacies, the averted cases per dose increased moderately but averted cases by percentage remaining roughly similar. We appended the Main Text Figure 4 below for reference, and supplemented the main text descriptions to describe these interactions:

*“Across uptake rates, higher durations of vaccine protection increased averted cases by percentage but kept averted cases per dose similar, whereas higher vaccine efficacy increased both averted cases by percentage and averted cases per dose. For jointly higher durations of protection and higher vaccine efficacies, only averted cases per dose increased moderately (Figure 4).”*

**Main Text Figure 4 Vaccine programme public health impact and efficiency under different vaccine profiles and uptake ratios** Results presented are relative to the baseline case of no vaccination programmes being implemented. Projections assume that transmission probabilities of 2024 to 2034 were kept constant at 2018 levels. The series of boxplots illustrate the differences in vaccination strategy impact by varying vaccine uptake rates, duration of protection, and vaccine efficacy. The uptake rates were set at 10%, 33%, and 100% in both school based and sexual health clinic vaccination programme strategies. Respectively, these are denoted (**A**) vaccination before entry [VbE], (**B**) vaccination on attendance [VoA], (**C**) vaccination on diagnoses with gonorrhoea [VoD], (**D**) vaccination according to risk [VaR], (**E**) VoA and (**F**) VoD strategies by offering vaccinations in these groups to only high sexual activity individuals (VoA[H]), VoD[H]). The point estimate (posterior mean) is marked with a square, and the 95% credible interval is indicated by the boundaries of the box.

Vaccine quality was set based on clinical observations with vaccine efficacy categorized as mild (M) at 22%, normal (N) at 31%, and strong (S) at 47%, with protection durations of 1.5 years, 4 years, and 7.5 years.

**Comment:** Sensitivity analyses focus primarily on vaccination parameters. Since behavioural parameters (e.g., screening rates, risk behaviour) play a crucial role in transmission dynamics, including them in the sensitivity analyses would provide a more comprehensive understanding of model behaviour under different scenarios.

**Responses:** We thank the reviewer for the comment. We have now included an additional sensitivity analysis, to change behavioural parameters by $\pm$25 and 50% in value (i.e assortativity parameter, screening rates) and re-projected the changes in gonorrhoea incidences in a 10 to 20 year time-frame and programme-specific values (i.e uptake rates, averted cases etc). Higher levels of assortativity and screening rate for asymptomatics increased impact of vaccination programmes (VoA, VoA(H) and VaR). However, as screening rates increased to very high levels (+50%), this increased impact on vaccination programmes seemed to taper off slightly:

| **Strategy** | **Averted cases**  **(total)** | **Averted cases**  **(%)** | **Averted cases**  **per dose** | **Number of vaccinations administered** | **Primary**  **uptake ratio** |
| --- | --- | --- | --- | --- | --- |
| VbE | 1648 (942-2120) | 14.18 (9.11-17.08) | 0.005 (0.003-0.006) | 172701 (172701-172701) | 100.00 (100.00-100.00) |
| VoD(H) | 1427 (551-2176) | 12.04 (7.12-15.00) | 0.24 (0.14-0.31) | 2954 (1674-3912) | 96.79 (95.47-97.69) |
| VoD | 1427 (551-2176) | 12.04 (7.12-15.00) | 0.22 (0.12-0.28) | 3317 (1853-4252) | 97.13 (95.63-97.96) |
| VoA | 4704 (1948-6947) | 40.26 (18.32-52.57) | 0.06 (0.02-0.10) | 45699 (13004-73327) | 91.62 (85.35-95.57) |
| VoA(H) | 4685 (1938-6922) | 40.10 (18.14-52.55) | 0.22 (0.12-0.28) | 11622 (5502-15669) | 87.13 (79.54-92.24) |
| VaR | 4685 (1939-6922) | 40.10 (18.14-52.55) | 0.21 (0.12-0.27) | 11874 (5906-15851) | 87.40 (79.73-92.61) |

**Main Text Table 4 Summary of population health impact for various vaccination programmes from 2024 to 2034.** We report posterior means and accompanying 95% credible intervals. Estimates presented are relative to the baseline scenario of no intervention, with transmission probabilities from 2024 to 2034 kept stable at 2018 levels.

| **Strategy** | **Averted cases**  **(total)** | **Averted cases**  **(%)** | **Averted cases**  **per dose** | **Number of vaccinations administered** | **Primary**  **uptake ratio** |
| --- | --- | --- | --- | --- | --- |
| VbE | 618 (0-1813) | 16.00 (6.32-21.78) | 0.002 (0.000-0.005) | 172701 (172701-172701) | 100.00 (100.00-100.00) |
| VoD(H) | 340 (0-1448) | 4.63 (0.00-12.35) | 0.09 (0.00-0.27) | 909 (0-2836) | 99.02 (96.29-100.00) |
| VoD | 340 (0-1449) | 4.63 (0.00-12.36) | 0.08 (0.00-0.22) | 1093 (0-3468) | 99.18 (96.93-100.00) |
| VoA | 1549 (0-5335) | 37.53 (11.56-54.56) | 0.02 (0.00-0.07) | 44641 (12405-72189) | 92.00 (85.52-96.17) |
| VoA(H) | 1539 (0-5292) | 37.29 (11.49-54.36) | 0.08 (0.00-0.24) | 10665 (3813-15111) | 88.20 (80.25-93.77) |
| VaR | 1539 (0-5292) | 37.30 (11.49-54.36) | 0.07 (0.00-0.23) | 10786 (3835-15282) | 88.33 (80.29-93.99) |

**Response Table Summary of population health impact for various vaccination programmes from 2024 to 2034, with a -50% change in the assortativity** **parameter** We report posterior means and accompanying 95% credible intervals. Estimates presented are relative to the baseline scenario of no intervention, with transmission probabilities from 2024 to 2034 kept stable at 2018 levels.

| **Strategy** | **Averted cases**  **(total)** | **Averted cases**  **(%)** | **Averted cases**  **per dose** | **Number of vaccinations administered** | **Primary**  **uptake ratio** |
| --- | --- | --- | --- | --- | --- |
| VbE | 948 (11-1900) | 17.50 (10.75-21.81) | 0.003 (0.000-0.006) | 172701 (172701-172701) | 100.00 (100.00-100.00) |
| VoD(H) | 549 (0-1635) | 7.17 (0.17-12.97) | 0.14 (0.00-0.28) | 1434 (22-3040) | 98.45 (96.12-99.93) |
| VoD | 549 (0-1636) | 7.18 (0.17-12.98) | 0.12 (0.00-0.23) | 1693 (23-3674) | 98.68 (96.78-99.94) |
| VoA | 2409 (23-5596) | 42.15 (18.31-54.51) | 0.03 (0.00-0.08) | 44906 (12652-72313) | 91.91 (85.49-96.04) |
| VoA(H) | 2395 (23-5561) | 41.95 (18.21-54.38) | 0.12 (0.00-0.25) | 10892 (4158-15231) | 87.95 (80.06-93.39) |
| VaR | 2396 (23-5562) | 41.95 (18.22-54.38) | 0.11 (0.00-0.24) | 11062 (4376-15475) | 88.13 (80.16-93.73) |

**Response Table Summary of population health impact for various vaccination programmes from 2024 to 2034, with a -25% change in the assortativity** **parameter** We report posterior means and accompanying 95% credible intervals. Estimates presented are relative to the baseline scenario of no intervention, with transmission probabilities from 2024 to 2034 kept stable at 2018 levels.

| **Strategy** | **Averted cases**  **(total)** | **Averted cases**  **(%)** | **Averted cases**  **per dose** | **Number of vaccinations administered** | **Primary**  **uptake ratio** |
| --- | --- | --- | --- | --- | --- |
| VbE | 1906 (1067-2530) | 11.37 (7.04-15.70) | 0.006 (0.003-0.007) | 172701 (172701-172701) | 100.00 (100.00-100.00) |
| VoD(H) | 2426 (713-4395) | 13.71 (7.97-17.66) | 0.28 (0.15-0.37) | 4364 (1945-6481) | 95.24 (92.28-97.22) |
| VoD | 2426 (714-4395) | 13.71 (7.97-17.66) | 0.26 (0.14-0.35) | 4722 (2324-6605) | 95.57 (92.35-97.67) |
| VoA | 6265 (2461-9736) | 36.58 (17.83-49.13) | 0.07 (0.03-0.13) | 46535 (13603-74063) | 91.26 (85.12-94.92) |
| VoA(H) | 6246 (2461-9716) | 36.46 (17.67-48.87) | 0.27 (0.15-0.36) | 12450 (6331-16380) | 86.19 (78.89-91.45) |
| VaR | 6246 (2461-9716) | 36.46 (17.68-48.87) | 0.26 (0.14-0.36) | 12712 (6563-16554) | 86.47 (79.04-91.91) |

**Response Table Summary of population health impact for various vaccination programmes from 2024 to 2034, with a +25% change in the assortativity** **parameter** We report posterior means and accompanying 95% credible intervals. Estimates presented are relative to the baseline scenario of no intervention, with transmission probabilities from 2024 to 2034 kept stable at 2018 levels.

| **Strategy** | **Averted cases**  **(total)** | **Averted cases**  **(%)** | **Averted cases**  **per dose** | **Number of vaccinations administered** | **Primary**  **uptake ratio** |
| --- | --- | --- | --- | --- | --- |
| VbE | 1975 (1084-2661) | 9.99 (5.96-15.25) | 0.006 (0.003-0.008) | 172701 (172701-172701) | 100.00 (100.00-100.00) |
| VoD(H) | 3053 (783-5572) | 14.25 (8.34-18.48) | 0.29 (0.16-0.39) | 5282 (2073-8035) | 94.22 (90.47-97.00) |
| VoD | 3054 (784-5572) | 14.25 (8.34-18.48) | 0.27 (0.14-0.38) | 5593 (2497-8073) | 94.51 (90.47-97.47) |
| VoA | 7021 (2461-11414) | 34.33 (16.97-47.79) | 0.08 (0.03-0.15) | 47101 (14848-74162) | 90.98 (85.07-94.72) |
| VoA(H) | 7004 (2461-11395) | 34.23 (16.97-47.68) | 0.29 (0.15-0.40) | 13035 (6473-17173) | 85.53 (78.28-91.23) |
| VaR | 7004 (2461-11396) | 34.23 (16.97-47.68) | 0.28 (0.15-0.39) | 13270 (6563-17349) | 85.78 (78.38-91.65) |

**Response Table Summary of population health impact for various vaccination programmes from 2024 to 2034, with a +50% change in the assortativity** **parameter** We report posterior means and accompanying 95% credible intervals. Estimates presented are relative to the baseline scenario of no intervention, with transmission probabilities from 2024 to 2034 kept stable at 2018 levels.

| **Strategy** | **Averted cases**  **(total)** | **Averted cases**  **(%)** | **Averted cases**  **per dose** | **Number of vaccinations administered** | **Primary**  **uptake ratio** |
| --- | --- | --- | --- | --- | --- |
| VbE | 1697 (955-2214) | 12.51 (8.18-15.21) | 0.005 (0.003-0.006) | 172701 (172701-172701) | 100.00 (100.00-100.00) |
| VoD(H) | 1659 (693-2507) | 12.03 (7.26-15.14) | 0.25 (0.14-0.33) | 3386 (2088-4295) | 96.31 (94.96-97.27) |
| VoD | 1659 (693-2507) | 12.03 (7.26-15.14) | 0.22 (0.12-0.28) | 3849 (2337-4864) | 96.73 (95.10-97.65) |
| VoA | 3621 (1540-5478) | 26.39 (14.12-33.96) | 0.05 (0.02-0.08) | 41970 (11478-68276) | 92.90 (86.56-96.46) |
| VoA(H) | 3589 (1529-5413) | 26.16 (13.92-33.79) | 0.24 (0.14-0.31) | 7788 (4174-10286) | 91.43 (86.96-94.48) |
| VaR | 3590 (1529-5414) | 26.17 (13.92-33.79) | 0.23 (0.13-0.30) | 8179 (4668-10709) | 91.83 (87.47-94.77) |

**Response Table Summary of population health impact for various vaccination programmes from 2024 to 2034, with a -50% change in the asymptomatic screening rate in high risk group** We report posterior means and accompanying 95% credible intervals. Estimates presented are relative to the baseline scenario of no intervention, with transmission probabilities from 2024 to 2034 kept stable at 2018 levels.

| **Strategy** | **Averted cases**  **(total)** | **Averted cases**  **(%)** | **Averted cases**  **per dose** | **Number of vaccinations administered** | **Primary**  **uptake ratio** |
| --- | --- | --- | --- | --- | --- |
| VbE | 1685 (973-2183) | 13.30 (8.62-16.14) | 0.005 (0.003-0.006) | 172701 (172701-172701) | 100.00 (100.00-100.00) |
| VoD(H) | 1561 (625-2370) | 12.11 (7.42-15.13) | 0.25 (0.14-0.32) | 3187 (1918-4096) | 96.54 (95.22-97.44) |
| VoD | 1561 (626-2371) | 12.11 (7.42-15.13) | 0.22 (0.12-0.28) | 3600 (2106-4536) | 96.91 (95.36-97.80) |
| VoA | 4287 (1799-6396) | 33.56 (16.35-43.86) | 0.05 (0.02-0.09) | 43861 (12061-70829) | 92.32 (86.06-96.03) |
| VoA(H) | 4262 (1790-6367) | 33.37 (16.17-43.65) | 0.23 (0.13-0.30) | 9735 (4872-13042) | 89.25 (83.17-93.34) |
| VaR | 4263 (1790-6368) | 33.37 (16.18-43.65) | 0.22 (0.13-0.29) | 10051 (5254-13387) | 89.58 (83.46-93.66) |

**Response Table Summary of population health impact for various vaccination programmes from 2024 to 2034, with a -25% change in the asymptomatic screening rate in high risk group** We report posterior means and accompanying 95% credible intervals. Estimates presented are relative to the baseline scenario of no intervention, with transmission probabilities from 2024 to 2034 kept stable at 2018 levels.

| **Strategy** | **Averted cases**  **(total)** | **Averted cases**  **(%)** | **Averted cases**  **per dose** | **Number of vaccinations administered** | **Primary**  **uptake ratio** |
| --- | --- | --- | --- | --- | --- |
| VbE | 1579 (856-2058) | 15.13 (9.65-18.34) | 0.005 (0.002-0.006) | 172701 (172701-172701) | 100.00 (100.00-100.00) |
| VoD(H) | 1260 (372-1969) | 11.77 (6.74-14.71) | 0.23 (0.13-0.30) | 2687 (1360-3698) | 97.09 (95.59-98.01) |
| VoD | 1260 (372-1970) | 11.78 (6.74-14.71) | 0.21 (0.11-0.27) | 3001 (1508-3994) | 97.38 (95.80-98.25) |
| VoA | 4837 (2031-6885) | 46.25 (20.41-59.58) | 0.06 (0.02-0.10) | 47488 (14086-75559) | 90.82 (84.49-95.06) |
| VoA(H) | 4823 (2022-6878) | 46.11 (20.29-59.50) | 0.20 (0.10-0.27) | 13452 (6086-18205) | 85.06 (76.15-91.24) |
| VaR | 4823 (2022-6878) | 46.11 (20.29-59.50) | 0.19 (0.10-0.26) | 13650 (6463-18359) | 85.28 (76.28-91.53) |

**Response Table Summary of population health impact for various vaccination programmes from 2024 to 2034, with a +25% change in the asymptomatic screening rate in high risk group** We report posterior means and accompanying 95% credible intervals. Estimates presented are relative to the baseline scenario of no intervention, with transmission probabilities from 2024 to 2034 kept stable at 2018 levels.

| **Strategy** | **Averted cases**  **(total)** | **Averted cases**  **(%)** | **Averted cases**  **per dose** | **Number of vaccinations administered** | **Primary**  **uptake ratio** |
| --- | --- | --- | --- | --- | --- |
| VbE | 1474 (639-1979) | 16.16 (10.14-19.68) | 0.004 (0.002-0.006) | 172701 (172701-172701) | 100.00 (100.00-100.00) |
| VoD(H) | 1067 (227-1774) | 11.26 (5.75-14.22) | 0.22 (0.10-0.29) | 2387 (1030-3429) | 97.42 (95.78-98.45) |
| VoD | 1067 (227-1775) | 11.26 (5.75-14.23) | 0.20 (0.09-0.26) | 2655 (1149-3725) | 97.66 (96.06-98.63) |
| VoA | 4688 (1933-6723) | 51.40 (22.72-64.48) | 0.05 (0.02-0.10) | 49224 (15113-77844) | 89.96 (83.50-94.48) |
| VoA(H) | 4677 (1925-6712) | 51.28 (22.60-64.35) | 0.17 (0.06-0.25) | 15222 (6663-20682) | 83.06 (73.03-90.26) |
| VaR | 4677 (1925-6712) | 51.29 (22.60-64.35) | 0.17 (0.06-0.24) | 15375 (7018-20739) | 83.23 (73.11-90.47) |

**Response Table Summary of population health impact for various vaccination programmes from 2024 to 2034, with a +50% change in the asymptomatic screening rate in high risk group** We report posterior means and accompanying 95% credible intervals. Estimates presented are relative to the baseline scenario of no intervention, with transmission probabilities from 2024 to 2034 kept stable at 2018 levels.

**Comment:** The model assumes that treated individuals (TjT) return to the uninfected state (Uj) with the same risk of infection as those who were never infected. This assumption overlooks the potential differences in behaviour, immunity, or vulnerability between previously infected and never-infected individuals. For example, individuals who are treated for gonorrhoea might have a different risk of reinfection due to changes in sexual networks, or the possibility of partial immunity from treatment.

**Responses:** We thank the reviewer for the comment and the helpful suggestion. This modification would entail incorporating separate recovered and once-infected compartments into our model structure across both high/low risk groups across unvaccinated/vaccine-protected and vaccine-protection waned compartments, which will significantly increase the complexity of the model. There is also no data on this granularity to parameterise the fine-scale potential differences in behaviour, immunity, or vulnerability between previously infected and never-infected individuals. The transition rates from uninfected to incubation stages therefore represent the weighted average transition rates of previously infected and never-infected individuals.

**Comment:** While the model acknowledges the presence of asymptomatic cases in the population, it does not fully explore their impact on the overall transmission dynamics. Asymptomatic individuals may unknowingly spread the infection over extended periods, potentially making them significant drivers of the epidemic. If asymptomatic individuals are not screened or treated as often, their role in sustaining transmission could be underestimated.

**Responses:** We thank the reviewer for the comment and agree that asymptomatic infections can potentially drive epidemics and influence transmissibility of disease. We have now included additional sensitivity analyses on all parameters related to asymptomatic individuals, i.e, we changed the initial asymptomatic prevalence, the rate of asymptomatic screening and probability that the incident infection is symptomatic by $\pm$25 and 50% in value and re-projected the changes in gonnorhoea incidences in a 10 year time-frame and programme-specific values (i.e uptake rates, averted cases etc):

| **Strategy** | **Averted cases**  **(total)** | **Averted cases**  **(%)** | **Averted cases**  **per dose** | **Number of vaccinations administered** | **Primary**  **uptake ratio** |
| --- | --- | --- | --- | --- | --- |
| VbE | 1697 (955-2214) | 12.51 (8.18-15.21) | 0.005 (0.003-0.006) | 172701 (172701-172701) | 100.00 (100.00-100.00) |
| VoD(H) | 1659 (693-2507) | 12.03 (7.26-15.14) | 0.25 (0.14-0.33) | 3386 (2088-4295) | 96.31 (94.96-97.27) |
| VoD | 1659 (693-2507) | 12.03 (7.26-15.14) | 0.22 (0.12-0.28) | 3849 (2337-4864) | 96.73 (95.10-97.65) |
| VoA | 3621 (1540-5478) | 26.39 (14.12-33.96) | 0.05 (0.02-0.08) | 41970 (11478-68276) | 92.90 (86.56-96.46) |
| VoA(H) | 3589 (1529-5413) | 26.16 (13.92-33.79) | 0.24 (0.14-0.31) | 7788 (4174-10286) | 91.43 (86.96-94.48) |
| VaR | 3590 (1529-5414) | 26.17 (13.92-33.79) | 0.23 (0.13-0.30) | 8179 (4668-10709) | 91.83 (87.47-94.77) |

**Response Table Summary of population health impact for various vaccination programmes from 2024 to 2034, with a -50% change in the asymptomatic screening rate in high risk group** We report posterior means and accompanying 95% credible intervals. Estimates presented are relative to the baseline scenario of no intervention, with transmission probabilities from 2024 to 2034 kept stable at 2018 levels.

| **Strategy** | **Averted cases**  **(total)** | **Averted cases**  **(%)** | **Averted cases**  **per dose** | **Number of vaccinations administered** | **Primary**  **uptake ratio** |
| --- | --- | --- | --- | --- | --- |
| VbE | 1685 (973-2183) | 13.30 (8.62-16.14) | 0.005 (0.003-0.006) | 172701 (172701-172701) | 100.00 (100.00-100.00) |
| VoD(H) | 1561 (625-2370) | 12.11 (7.42-15.13) | 0.25 (0.14-0.32) | 3187 (1918-4096) | 96.54 (95.22-97.44) |
| VoD | 1561 (626-2371) | 12.11 (7.42-15.13) | 0.22 (0.12-0.28) | 3600 (2106-4536) | 96.91 (95.36-97.80) |
| VoA | 4287 (1799-6396) | 33.56 (16.35-43.86) | 0.05 (0.02-0.09) | 43861 (12061-70829) | 92.32 (86.06-96.03) |
| VoA(H) | 4262 (1790-6367) | 33.37 (16.17-43.65) | 0.23 (0.13-0.30) | 9735 (4872-13042) | 89.25 (83.17-93.34) |
| VaR | 4263 (1790-6368) | 33.37 (16.18-43.65) | 0.22 (0.13-0.29) | 10051 (5254-13387) | 89.58 (83.46-93.66) |

**Response Table Summary of population health impact for various vaccination programmes from 2024 to 2034, with a -25% change in the asymptomatic screening rate in high risk group** We report posterior means and accompanying 95% credible intervals. Estimates presented are relative to the baseline scenario of no intervention, with transmission probabilities from 2024 to 2034 kept stable at 2018 levels.

| **Strategy** | **Averted cases**  **(total)** | **Averted cases**  **(%)** | **Averted cases**  **per dose** | **Number of vaccinations administered** | **Primary**  **uptake ratio** |
| --- | --- | --- | --- | --- | --- |
| VbE | 1579 (856-2058) | 15.13 (9.65-18.34) | 0.005 (0.002-0.006) | 172701 (172701-172701) | 100.00 (100.00-100.00) |
| VoD(H) | 1260 (372-1969) | 11.77 (6.74-14.71) | 0.23 (0.13-0.30) | 2687 (1360-3698) | 97.09 (95.59-98.01) |
| VoD | 1260 (372-1970) | 11.78 (6.74-14.71) | 0.21 (0.11-0.27) | 3001 (1508-3994) | 97.38 (95.80-98.25) |
| VoA | 4837 (2031-6885) | 46.25 (20.41-59.58) | 0.06 (0.02-0.10) | 47488 (14086-75559) | 90.82 (84.49-95.06) |
| VoA(H) | 4823 (2022-6878) | 46.11 (20.29-59.50) | 0.20 (0.10-0.27) | 13452 (6086-18205) | 85.06 (76.15-91.24) |
| VaR | 4823 (2022-6878) | 46.11 (20.29-59.50) | 0.19 (0.10-0.26) | 13650 (6463-18359) | 85.28 (76.28-91.53) |

**Response Table Summary of population health impact for various vaccination programmes from 2024 to 2034, with a +25% change in the asymptomatic screening rate in high risk group** We report posterior means and accompanying 95% credible intervals. Estimates presented are relative to the baseline scenario of no intervention, with transmission probabilities from 2024 to 2034 kept stable at 2018 levels.

| **Strategy** | **Averted cases**  **(total)** | **Averted cases**  **(%)** | **Averted cases**  **per dose** | **Number of vaccinations administered** | **Primary**  **uptake ratio** |
| --- | --- | --- | --- | --- | --- |
| VbE | 1474 (639-1979) | 16.16 (10.14-19.68) | 0.004 (0.002-0.006) | 172701 (172701-172701) | 100.00 (100.00-100.00) |
| VoD(H) | 1067 (227-1774) | 11.26 (5.75-14.22) | 0.22 (0.10-0.29) | 2387 (1030-3429) | 97.42 (95.78-98.45) |
| VoD | 1067 (227-1775) | 11.26 (5.75-14.23) | 0.20 (0.09-0.26) | 2655 (1149-3725) | 97.66 (96.06-98.63) |
| VoA | 4688 (1933-6723) | 51.40 (22.72-64.48) | 0.05 (0.02-0.10) | 49224 (15113-77844) | 89.96 (83.50-94.48) |
| VoA(H) | 4677 (1925-6712) | 51.28 (22.60-64.35) | 0.17 (0.06-0.25) | 15222 (6663-20682) | 83.06 (73.03-90.26) |
| VaR | 4677 (1925-6712) | 51.29 (22.60-64.35) | 0.17 (0.06-0.24) | 15375 (7018-20739) | 83.23 (73.11-90.47) |

**Response Table Summary of population health impact for various vaccination programmes from 2024 to 2034, with a +50% change in the asymptomatic screening rate in high risk group** We report posterior means and accompanying 95% credible intervals. Estimates presented are relative to the baseline scenario of no intervention, with transmission probabilities from 2024 to 2034 kept stable at 2018 levels.

| **Strategy** | **Averted cases**  **(total)** | **Averted cases**  **(%)** | **Averted cases**  **per dose** | **Number of vaccinations administered** | **Primary**  **uptake ratio** |
| --- | --- | --- | --- | --- | --- |
| VbE | 1648 (942-2120) | 14.18 (9.11-17.08) | 0.005 (0.003-0.006) | 172701 (172701-172701) | 100.00 (100.00-100.00) |
| VoD(H) | 1427 (551-2176) | 12.04 (7.12-15.00) | 0.24 (0.14-0.31) | 2954 (1674-3912) | 96.79 (95.47-97.69) |
| VoD | 1427 (551-2176) | 12.04 (7.12-15.00) | 0.22 (0.12-0.28) | 3317 (1853-4252) | 97.13 (95.63-97.96) |
| VoA | 4704 (1948-6947) | 40.26 (18.32-52.57) | 0.06 (0.02-0.10) | 45699 (13004-73327) | 91.62 (85.35-95.57) |
| VoA(H) | 4685 (1938-6922) | 40.10 (18.14-52.55) | 0.22 (0.12-0.28) | 11622 (5502-15669) | 87.13 (79.54-92.24) |
| VaR | 4685 (1939-6922) | 40.10 (18.14-52.55) | 0.21 (0.12-0.27) | 11874 (5906-15851) | 87.40 (79.73-92.61) |

**Response Table Summary of population health impact for various vaccination programmes from 2024 to 2034, with a -50% change in the initial prevalence of asymptomatic infection in the low risk group.** We report posterior means and accompanying 95% credible intervals. Estimates presented are relative to the baseline scenario of no intervention, with transmission probabilities from 2024 to 2034 kept stable at 2018 levels.

| **Strategy** | **Averted cases**  **(total)** | **Averted cases**  **(%)** | **Averted cases**  **per dose** | **Number of vaccinations administered** | **Primary**  **uptake ratio** |
| --- | --- | --- | --- | --- | --- |
| VbE | 1648 (942-2120) | 14.18 (9.11-17.08) | 0.005 (0.003-0.006) | 172701 (172701-172701) | 100.00 (100.00-100.00) |
| VoD(H) | 1427 (551-2176) | 12.04 (7.12-15.00) | 0.24 (0.14-0.31) | 2954 (1674-3912) | 96.79 (95.47-97.69) |
| VoD | 1427 (551-2176) | 12.04 (7.12-15.00) | 0.22 (0.12-0.28) | 3317 (1853-4252) | 97.13 (95.63-97.96) |
| VoA | 4704 (1948-6947) | 40.26 (18.32-52.57) | 0.06 (0.02-0.10) | 45699 (13004-73327) | 91.62 (85.35-95.57) |
| VoA(H) | 4685 (1938-6922) | 40.10 (18.14-52.55) | 0.22 (0.12-0.28) | 11622 (5502-15669) | 87.13 (79.54-92.24) |
| VaR | 4685 (1939-6922) | 40.10 (18.14-52.55) | 0.21 (0.12-0.27) | 11874 (5906-15851) | 87.40 (79.73-92.61) |

**Response Table Summary of population health impact for various vaccination programmes from 2024 to 2034, with a -25% change in the initial prevalence of asymptomatic infection in the low risk group.** We report posterior means and accompanying 95% credible intervals. Estimates presented are relative to the baseline scenario of no intervention, with transmission probabilities from 2024 to 2034 kept stable at 2018 levels.

| **Strategy** | **Averted cases**  **(total)** | **Averted cases**  **(%)** | **Averted cases**  **per dose** | **Number of vaccinations administered** | **Primary**  **uptake ratio** |
| --- | --- | --- | --- | --- | --- |
| VbE | 1648 (942-2120) | 14.18 (9.11-17.08) | 0.005 (0.003-0.006) | 172701 (172701-172701) | 100.00 (100.00-100.00) |
| VoD(H) | 1427 (551-2176) | 12.04 (7.12-15.00) | 0.24 (0.14-0.31) | 2954 (1674-3912) | 96.79 (95.47-97.69) |
| VoD | 1427 (551-2176) | 12.04 (7.12-15.00) | 0.22 (0.12-0.28) | 3317 (1853-4252) | 97.13 (95.63-97.96) |
| VoA | 4704 (1948-6947) | 40.26 (18.32-52.57) | 0.06 (0.02-0.10) | 45699 (13004-73327) | 91.62 (85.35-95.57) |
| VoA(H) | 4685 (1938-6922) | 40.10 (18.14-52.55) | 0.22 (0.12-0.28) | 11622 (5502-15669) | 87.13 (79.54-92.24) |
| VaR | 4685 (1939-6922) | 40.10 (18.14-52.55) | 0.21 (0.12-0.27) | 11874 (5906-15851) | 87.40 (79.73-92.61) |

**Response Table Summary of population health impact for various vaccination programmes from 2024 to 2034, with a +25% change in the initial prevalence of asymptomatic infection in the low risk group.** We report posterior means and accompanying 95% credible intervals. Estimates presented are relative to the baseline scenario of no intervention, with transmission probabilities from 2024 to 2034 kept stable at 2018 levels.

| **Strategy** | **Averted cases**  **(total)** | **Averted cases**  **(%)** | **Averted cases**  **per dose** | **Number of vaccinations administered** | **Primary**  **uptake ratio** |
| --- | --- | --- | --- | --- | --- |
| VbE | 1648 (942-2120) | 14.18 (9.11-17.08) | 0.005 (0.003-0.006) | 172701 (172701-172701) | 100.00 (100.00-100.00) |
| VoD(H) | 1427 (551-2176) | 12.04 (7.12-15.00) | 0.24 (0.14-0.31) | 2954 (1674-3912) | 96.79 (95.47-97.69) |
| VoD | 1427 (551-2176) | 12.04 (7.12-15.00) | 0.22 (0.12-0.28) | 3317 (1853-4252) | 97.13 (95.63-97.96) |
| VoA | 4704 (1948-6947) | 40.26 (18.32-52.57) | 0.06 (0.02-0.10) | 45699 (13004-73327) | 91.62 (85.35-95.57) |
| VoA(H) | 4685 (1938-6922) | 40.10 (18.14-52.55) | 0.22 (0.12-0.28) | 11622 (5502-15669) | 87.13 (79.54-92.24) |
| VaR | 4685 (1939-6922) | 40.10 (18.14-52.55) | 0.21 (0.12-0.27) | 11874 (5906-15851) | 87.40 (79.73-92.61) |

**Response Table Summary of population health impact for various vaccination programmes from 2024 to 2034, with a +50% change in the initial prevalence of asymptomatic infection in the low risk group.** We report posterior means and accompanying 95% credible intervals. Estimates presented are relative to the baseline scenario of no intervention, with transmission probabilities from 2024 to 20˙34 kept stable at 2018 levels.

| **Strategy** | **Averted cases**  **(total)** | **Averted cases**  **(%)** | **Averted cases**  **per dose** | **Number of vaccinations administered** | **Primary**  **uptake ratio** |
| --- | --- | --- | --- | --- | --- |
| VbE | 1647 (942-2120) | 14.18 (9.11-17.09) | 0.005 (0.003-0.006) | 172701 (172701-172701) | 100.00 (100.00-100.00) |
| VoD(H) | 1425 (550-2176) | 12.03 (7.12-14.99) | 0.24 (0.14-0.31) | 2952 (1671-3906) | 96.80 (95.47-97.69) |
| VoD | 1426 (550-2176) | 12.04 (7.12-14.99) | 0.22 (0.12-0.28) | 3315 (1852-4247) | 97.13 (95.63-97.96) |
| VoA | 4702 (1948-6941) | 40.27 (18.32-52.58) | 0.06 (0.02-0.10) | 45698 (13003-73326) | 91.62 (85.35-95.57) |
| VoA(H) | 4683 (1938-6920) | 40.11 (18.14-52.55) | 0.22 (0.12-0.28) | 11621 (5499-15663) | 87.13 (79.54-92.24) |
| VaR | 4683 (1938-6921) | 40.11 (18.14-52.55) | 0.21 (0.12-0.27) | 11873 (5903-15849) | 87.40 (79.73-92.61) |

**Response Table Summary of population health impact for various vaccination programmes from 2024 to 2034, with a -50% change in the initial prevalence of asymptomatic infection in the high risk group.** We report posterior means and accompanying 95% credible intervals. Estimates presented are relative to the baseline scenario of no intervention, with transmission probabilities from 2024 to 2034 kept stable at 2018 levels.

| **Strategy** | **Averted cases**  **(total)** | **Averted cases**  **(%)** | **Averted cases**  **per dose** | **Number of vaccinations administered** | **Primary**  **uptake ratio** |
| --- | --- | --- | --- | --- | --- |
| VbE | 1647 (942-2120) | 14.18 (9.11-17.08) | 0.005 (0.003-0.006) | 172701 (172701-172701) | 100.00 (100.00-100.00) |
| VoD(H) | 1426 (551-2176) | 12.04 (7.12-14.99) | 0.24 (0.14-0.31) | 2953 (1673-3910) | 96.79 (95.47-97.69) |
| VoD | 1427 (551-2176) | 12.04 (7.12-14.99) | 0.22 (0.12-0.28) | 3317 (1852-4250) | 97.13 (95.63-97.96) |
| VoA | 4703 (1948-6945) | 40.26 (18.32-52.57) | 0.06 (0.02-0.10) | 45699 (13004-73327) | 91.62 (85.35-95.57) |
| VoA(H) | 4684 (1938-6922) | 40.10 (18.14-52.55) | 0.22 (0.12-0.28) | 11622 (5501-15667) | 87.13 (79.54-92.24) |
| VaR | 4684 (1938-6922) | 40.10 (18.14-52.55) | 0.21 (0.12-0.27) | 11874 (5905-15851) | 87.40 (79.73-92.61) |

**Response Table Summary of population health impact for various vaccination programmes from 2024 to 2034, with a -25% change in initial prevalence of asymptomatic infection in the high risk group.** We report posterior means and accompanying 95% credible intervals. Estimates presented are relative to the baseline scenario of no intervention, with transmission probabilities from 2024 to 2034 kept stable at 2018 levels.

| **Strategy** | **Averted cases**  **(total)** | **Averted cases**  **(%)** | **Averted cases**  **per dose** | **Number of vaccinations administered** | **Primary**  **uptake ratio** |
| --- | --- | --- | --- | --- | --- |
| VbE | 1648 (942-2120) | 14.17 (9.11-17.07) | 0.005 (0.003-0.006) | 172701 (172701-172701) | 100.00 (100.00-100.00) |
| VoD(H) | 1427 (551-2177) | 12.04 (7.12-15.00) | 0.24 (0.14-0.31) | 2954 (1674-3913) | 96.79 (95.47-97.69) |
| VoD | 1428 (551-2177) | 12.04 (7.12-15.00) | 0.22 (0.12-0.28) | 3318 (1853-4253) | 97.13 (95.63-97.96) |
| VoA | 4704 (1948-6948) | 40.26 (18.32-52.57) | 0.06 (0.02-0.10) | 45700 (13004-73328) | 91.62 (85.35-95.57) |
| VoA(H) | 4685 (1939-6922) | 40.09 (18.14-52.55) | 0.22 (0.12-0.28) | 11623 (5502-15671) | 87.13 (79.54-92.24) |
| VaR | 4685 (1939-6923) | 40.09 (18.14-52.55) | 0.21 (0.12-0.27) | 11874 (5907-15852) | 87.40 (79.73-92.61) |

**Response Table Summary of population health impact for various vaccination programmes from 2024 to 2034, with a +25% change in the initial prevalence of asymptomatic infection in the high risk group.** We report posterior means and accompanying 95% credible intervals. Estimates presented are relative to the baseline scenario of no intervention, with transmission probabilities from 2024 to 2034 kept stable at 2018 levels.

| **Strategy** | **Averted cases**  **(total)** | **Averted cases**  **(%)** | **Averted cases**  **per dose** | **Number of vaccinations administered** | **Primary**  **uptake ratio** |
| --- | --- | --- | --- | --- | --- |
| VbE | 1648 (942-2120) | 14.17 (9.11-17.07) | 0.005 (0.003-0.006) | 172701 (172701-172701) | 100.00 (100.00-100.00) |
| VoD(H) | 1428 (551-2177) | 12.04 (7.12-15.00) | 0.24 (0.14-0.31) | 2955 (1674-3914) | 96.79 (95.47-97.69) |
| VoD | 1428 (551-2177) | 12.04 (7.12-15.00) | 0.22 (0.12-0.28) | 3318 (1854-4253) | 97.13 (95.63-97.96) |
| VoA | 4705 (1948-6948) | 40.25 (18.32-52.57) | 0.06 (0.02-0.10) | 45700 (13004-73328) | 91.62 (85.35-95.57) |
| VoA(H) | 4685 (1939-6923) | 40.09 (18.14-52.55) | 0.22 (0.12-0.28) | 11623 (5502-15672) | 87.13 (79.54-92.24) |
| VaR | 4686 (1939-6923) | 40.09 (18.14-52.55) | 0.21 (0.12-0.27) | 11875 (5907-15852) | 87.40 (79.73-92.61) |

**Response Table Summary of population health impact for various vaccination programmes from 2024 to 2034, with a +50% change in the initial prevalence of asymptomatic infection in the high risk group.** We report posterior means and accompanying 95% credible intervals. Estimates presented are relative to the baseline scenario of no intervention, with transmission probabilities from 2024 to 2034 kept stable at 2018 levels.

| **Strategy** | **Averted cases**  **(total)** | **Averted cases**  **(%)** | **Averted cases**  **per dose** | **Number of vaccinations administered** | **Primary**  **uptake ratio** |
| --- | --- | --- | --- | --- | --- |
| VbE | 1249 (705-1625) | 8.50 (6.30-10.06) | 0.004 (0.002-0.005) | 172701 (172701-172701) | 100.00 (100.00-100.00) |
| VoD(H) | 1352 (547-2257) | 8.97 (5.47-11.22) | 0.18 (0.10-0.23) | 3828 (2405-5124) | 95.83 (94.05-97.00) |
| VoD | 1353 (547-2257) | 8.97 (5.48-11.23) | 0.16 (0.09-0.20) | 4307 (2807-5708) | 96.27 (94.36-97.37) |
| VoA | 4137 (1387-6499) | 27.83 (11.84-39.24) | 0.05 (0.02-0.08) | 46126 (13883-73512) | 91.46 (85.30-95.37) |
| VoA(H) | 4108 (1372-6457) | 27.63 (11.69-39.02) | 0.18 (0.10-0.24) | 11977 (6076-15965) | 86.72 (79.42-91.95) |
| VaR | 4108 (1372-6458) | 27.64 (11.70-39.03) | 0.18 (0.10-0.23) | 12362 (6468-16417) | 87.13 (79.68-92.37) |

**Response Table Summary of population health impact for various vaccination programmes from 2024 to 2034, with a -50% change in the number of asymptomatics per incident infection.** We report posterior means and accompanying 95% credible intervals. Estimates presented are relative to the baseline scenario of no intervention, with transmission probabilities from 2024 to 2034 kept stable at 2018 levels.

| **Strategy** | **Averted cases**  **(total)** | **Averted cases**  **(%)** | **Averted cases**  **per dose** | **Number of vaccinations administered** | **Primary**  **uptake ratio** |
| --- | --- | --- | --- | --- | --- |
| VbE | 1593 (917-2100) | 10.68 (7.37-12.61) | 0.005 (0.003-0.006) | 172701 (172701-172701) | 100.00 (100.00-100.00) |
| VoD(H) | 1725 (695-2811) | 11.31 (6.98-14.30) | 0.23 (0.13-0.31) | 3774 (2445-4839) | 95.89 (94.28-96.97) |
| VoD | 1726 (695-2812) | 11.32 (6.98-14.31) | 0.20 (0.12-0.27) | 4249 (2774-5485) | 96.33 (94.54-97.39) |
| VoA | 5106 (1863-7800) | 34.01 (15.05-46.01) | 0.06 (0.03-0.10) | 46199 (13928-73587) | 91.43 (85.30-95.21) |
| VoA(H) | 5078 (1852-7744) | 33.83 (14.97-45.76) | 0.23 (0.13-0.30) | 12049 (6269-16047) | 86.64 (79.37-91.82) |
| VaR | 5078 (1852-7744) | 33.83 (14.97-45.77) | 0.22 (0.13-0.29) | 12407 (6795-16357) | 87.02 (79.62-92.15) |

**Response Table Summary of population health impact for various vaccination programmes from 2024 to 2034, with a -25% change in the number of asymptomatics per incident infection.** We report posterior means and accompanying 95% credible intervals. Estimates presented are relative to the baseline scenario of no intervention, with transmission probabilities from 2024 to 2034 kept stable at 2018 levels.

| **Strategy** | **Averted cases**  **(total)** | **Averted cases**  **(%)** | **Averted cases**  **per dose** | **Number of vaccinations administered** | **Primary**  **uptake ratio** |
| --- | --- | --- | --- | --- | --- |
| VbE | 917 (61-1640) | 18.75 (11.16-22.35) | 0.003 (0.000-0.005) | 172701 (172701-172701) | 100.00 (100.00-100.00) |
| VoD(H) | 447 (3-1259) | 7.65 (0.75-12.19) | 0.14 (0.01-0.24) | 1353 (102-2685) | 98.54 (96.15-99.68) |
| VoD | 447 (3-1259) | 7.65 (0.75-12.19) | 0.13 (0.01-0.22) | 1512 (117-2963) | 98.68 (96.50-99.72) |
| VoA | 2231 (130-4651) | 44.05 (18.80-56.70) | 0.03 (0.00-0.06) | 44808 (11875-72591) | 91.94 (85.44-96.10) |
| VoA(H) | 2223 (129-4636) | 43.90 (18.73-56.61) | 0.11 (0.01-0.21) | 10848 (4077-15234) | 88.00 (79.87-93.60) |
| VaR | 2223 (129-4636) | 43.90 (18.73-56.61) | 0.11 (0.01-0.20) | 10948 (4225-15263) | 88.11 (79.97-93.66) |

**Response Table Summary of population health impact for various vaccination programmes from 2024 to 2034, with a +25% change in the number of asymptomatics per incident infection.** We report posterior means and accompanying 95% credible intervals. Estimates presented are relative to the baseline scenario of no intervention, with transmission probabilities from 2024 to 2034 kept stable at 2018 levels.

| **Strategy** | **Averted cases**  **(total)** | **Averted cases**  **(%)** | **Averted cases**  **per dose** | **Number of vaccinations administered** | **Primary**  **uptake ratio** |
| --- | --- | --- | --- | --- | --- |
| VbE | 170 (0-912) | 16.53 (7.57-21.94) | 0.000 (0.000-0.003) | 172701 (172701-172701) | 100.00 (100.00-100.00) |
| VoD(H) | 45 (0-364) | 1.68 (0.00-7.63) | 0.03 (0.00-0.14) | 259 (0-1319) | 99.72 (97.15-100.00) |
| VoD | 45 (0-364) | 1.68 (0.00-7.63) | 0.03 (0.00-0.13) | 288 (0-1439) | 99.75 (97.35-100.00) |
| VoA | 382 (0-2140) | 36.48 (11.24-53.26) | 0.00 (0.00-0.03) | 44285 (10734-72153) | 92.12 (85.51-96.37) |
| VoA(H) | 381 (0-2139) | 36.34 (11.24-53.08) | 0.02 (0.00-0.11) | 10389 (3183-14872) | 88.51 (80.22-94.07) |
| VaR | 381 (0-2139) | 36.34 (11.24-53.08) | 0.02 (0.00-0.11) | 10407 (3201-14872) | 88.53 (80.25-94.22) |

**Response Table Summary of population health impact for various vaccination programmes from 2024 to 2034, with a +50% change in the number of asymptomatics per incident infection.** We report posterior means and accompanying 95% credible intervals. Estimates presented are relative to the baseline scenario of no intervention, with transmission probabilities from 2024 to 2034 kept stable at 2018 levels.

**Comment:** The authors mention the use of Bayesian methods for parameter estimation but provide limited details on the prior distributions used for key parameters (e.g., transmission rate β\beta, recovery rate, vaccine efficacy). Additionally, sensitivity to different prior assumptions could be tested, especially if certain priors were based on weak or uncertain evidence.

**Responses:** We thank the reviewer for the comment. We have provided information on the prior distributions used for the parameters in the supplementary information tables S3,4 (attached below). The priors used for behavioural parameters were uninformative with a wide prior support and would not significantly influence the posterior estimates. These included the level of assortativity and rate of screening. Whereas priors on the transmission parameters such as the incubation period, natural recovery rate and asymptomatic rate were based on the original study by Whittles et al. 2022. These prior distributions had wide prior support as well and are not expected to change significantly between study settings and at-risk populations.

**Table S3**: Fitted demographic parameters: notation, definition, prior distribution, and posterior estimates.

| **θ** | **Definition** | **Prior distribution** | **Parameter bounds** | **Posterior estimate** | | |
| --- | --- | --- | --- | --- | --- | --- |
|  |  |  |  | Main scenario | Upper bound | Lower bound |
| $\beta_{2004}$ | Probability of transmission  per-partnership in 2004 | U [0, 100%] | [0, 100%] | 44.44% (33.39%, 52.81%) | 41.05% (30.44%, 52.28%) | 39.94% (29.65%, 50.76%) |
| $\beta_{2018}$ | Probability of transmission  per-partnership in 2018 | U [0, 100%] | [0, 100%] | 44.30% (32.66%, 53.05%) | 40.21% (28.97%, 51.86%) | 39.65% (29.25%, 50.68%) |
| $A_{L}\left( t0 \right)/N_{L}$ | Initial prevalence of  asymptomatic infection in group L | U [0, 1.5%](Main)  U [0, 1.8%](Upper)  U [0, 1.3%](Lower) | [0, 1.5%](Main)  [0, 1.8%](Upper)  [0, 1.3%](Lower) | 0.74% (0.03%, 1.46%) | 0.77% (0.03%, 1.74%) | 0.55% (0.02%, 1.26%) |
| $A_{H}\left( t0 \right)/N_{H}$ | Initial prevalence of asymptomatic infection in group H | U [0, 8.6%](Main)  U [0, 10.6%](Upper)  U [0, 7.4%](Lower) | [0, 8.6%](Main)  [0, 10.6%](Upper)  [0, 7.4%](Lower) | 7.70% (5.65%, 8.57%) | 9.38% (6.99%, 10.56%) | 6.63 (4.96%, 7.37%) |
| $\varepsilon$ | Level of assortativity in sexual mixing | U [0, 1] | [0, 1] | 0.40 (0.02, 0.96) | 0.40 (0.01, 0.94) | 0.45 (0.02, 0.96) |
| $\eta_{H}$ | Rate of asymptomatic screening  in group H | U [0,4] | [0, 4] | 0.17 (0.08, 0.26) | 0.17 (0.08, 0.27) | 0.17 (0.08, 0.26) |

**Table S3**: Fitted transmission parameters: notation, definition, prior distribution, parameter bounds, and posterior estimates. Transition rate parameters ($\theta\epsilon\{\nu, \sigma, \mu,\rho\}$) are presented on an annual basis, giving a mean time to transition of $365/\theta$ days.

| **θ** | **Definition** | **Prior distribution** | **Parameter bounds** | **Posterior estimate** | | |
| --- | --- | --- | --- | --- | --- | --- |
|  |  |  |  | Main scenario | Upper bound | Lower bound |
| $\omega$ | Ratio of screening rate  in group L vs H | N(0.475, 0.168)* | [0, 1] | 0.55 (0.25, 0.89) | 0.55 (0.22, 0.87) | 0.61 (0.29, 0.93) |
| $\nu$ | Rate of natural recovery  (A → U) | N (3.08, 1.13) * | [3, 6] | 5.17 (3.75, 5.97) | 4.54 (3.24, 5.85) | 4.63 (3.33, 5.84) |
| $\sigma$ | Rate of leaving incubation period (I → S/A) | N (99.9, 30.587) * | [26, 180] | 109.62 (53.57, 163.92) | 107.48 (54.12, 166.41) | 104.35 (49.62, 161.74) |
| $\mu$ | Rate of seeking treatment  due to symptoms (S → T) | N (218, 109.21) * | [52, 365] | 217.93 (72.60, 347.18) | 216.79 (70.97, 350.30) | 221.16 (75.53, 351.02) |
| $\psi$ | Probability that incident infection is symptomatic | N (0.15, 0.0419) * | [0.1, 0.2] | 0.14 (0.11, 0.18) | 0.14 (0.10, 0.19) | 0.14 (0.11, 0.19) |
| $\rho$ | Rate of recovery after treatment  (T → U) | N (54, 5.816) * | [12, 90] | 53.99 (43.34, 65.58) | 55.42 (44.48, 66.58) | 54.84 (43.86, 65.99) |
| $\kappa_{D}$ | Shape parameter of communicable disease surveillance data | U [0, 1] | [0, 1] | 0.03 (0.01, 0.09) | 0.03 (0.01, 0.09) | 0.03 (0.01, 0.10) |

** Gonorrhoea transmissional parameters’ prior distributions referring previous study in a separate UK context*[3]*.*

**Comment:** The formulation of the force of infection (λj\lambda) is correct, but the description in the text could be clearer. Specifically, more detail could be provided on the implications of the assortative parameter (ϵ\epsilon) and the proportionate mixing assumption. Providing a plot or example of how varying assortative affects transmission dynamics would improve the reader's understanding.

**Responses:** We thank the reviewer for the comment. We have now provided plots on how the assortative parameter and proportionate mixing assumptions can potentially affect transmission dynamics by providing a sensitivity analyses. We have varied this parameter by -50,-25,25 and 50% and examined the number of individuals in each compartment. Higher levels of assortativity increased the incidence of gonorrhoea in the high risk groups moderately:

| **Category** | **High risk** | **Low risk** | **Total** |
| --- | --- | --- | --- |
| Screened | 35234 (9469-54051) | 111617 (20972-202991) | 146852 (32628-251268) |
| Screened (%) | 25.29 (15.26-41.05) | 74.71 (42.69-83.47) |  |
| Incidence | 19200 (0-65654) | 4155 (0-14958) | 23355 (0-80615) |
| Incidence (%) | 83.59 (81.22-86.27) | 16.41 (13.59-18.55) |  |
| Treated | 3081 (0-10049) | 615 (0-2122) | 3696 (0-12203) |
| Treated (%) | 84.64 (81.87-87.19) | 15.36 (12.28-17.77) |  |
| Asymptomatic and diagnosed | 523 (0-1983) | 61 (0-263) | 585 (0-2196) |
| Asymptomatic diagnosed (%) | 90.19 (83.52-95.48) | 9.81 (2.64-14.75) |  |
| Symptomatic and diagnosed | 2558 (0-8534) | 553 (0-1931) | 3111 (0-10475) |
| Symptomatic and diagnosed (%) | 83.59 (81.22-86.27) | 16.41 (13.59-18.55) |  |
| Treated/Incidence (%) | 16.76 (12.25-21.15) | 15.51 (11.08-19.93) | 16.55 (12.06-20.88) |
| Asymptomatic and treated (%) | 17.11 (4.75-28.22) | 10.36 (1.71-20.28) | 16.09 (4.39-26.71) |
| Symptomatic and treated (%) | 82.89 (68.09-92.43) | 89.64 (75.69-96.71) | 83.91 (69.63-93.07) |

**Response Table : Summary of number of individuals transitioning to each compartment in the transmission model by risk group from 2024 – 2034** Results presented are relative to the main baseline scenario, where no vaccination programmes were implemented. Transmission probabilities of 2024 to 2034 were kept constant at 2018 levels. Assortativity was changed by -50% from estimated levels from the initial model.

| **Category** | **High risk** | **Low risk** | **Total** |
| --- | --- | --- | --- |
| Screened | 34905 (9331-53435) | 111590 (20966-202978) | 146494 (32503-250849) |
| Screened (%) | 25.12 (15.03-40.75) | 74.88 (43.05-83.58) |  |
| Incidence | 30162 (355-69272) | 5824 (33-15299) | 35986 (387-84501) |
| Incidence (%) | 85.67 (81.27-91.61) | 14.33 (8.06-18.46) |  |
| Treated | 4884 (65-10832) | 869 (6-2172) | 5754 (71-12838) |
| Treated (%) | 86.60 (82.10-92.11) | 13.40 (7.45-17.54) |  |
| Asymptomatic and diagnosed | 827 (7-2221) | 87 (0-278) | 914 (8-2460) |
| Asymptomatic diagnosed (%) | 91.53 (83.78-96.51) | 8.47 (2.33-14.07) |  |
| Symptomatic and diagnosed | 4057 (53-9155) | 782 (5-2014) | 4840 (58-11104) |
| Symptomatic and diagnosed (%) | 85.67 (81.27-91.61) | 14.33 (8.06-18.46) |  |
| Treated/Incidence (%) | 16.69 (12.25-21.07) | 15.46 (11.08-19.88) | 16.52 (12.07-20.85) |
| Asymptomatic and treated (%) | 16.91 (4.75-27.91) | 10.22 (1.70-19.77) | 16.02 (4.40-26.50) |
| Symptomatic and treated (%) | 83.09 (68.33-92.49) | 89.78 (76.56-96.71) | 83.98 (69.70-93.07) |

**Response Table : Summary of number of individuals transitioning to each compartment in the transmission model by risk group from 2024 – 2034** Results presented are relative to the main baseline scenario, where no vaccination programmes were implemented. Transmission probabilities of 2024 to 2034 were kept constant at 2018 levels. Assortativity was changed by -25% from estimated levels from the initial model.

| **Category** | **High risk** | **Low risk** | **Total** |
| --- | --- | --- | --- |
| Screened | 32903 (8730-50579) | 111551 (20952-202991) | 144453 (32098-246360) |
| Screened (%) | 24.06 (14.07-39.47) | 75.94 (44.21-84.67) |  |
| Incidence | 96489 (39338-142942) | 8285 (-0-17330) | 104774 (47837-147828) |
| Incidence (%) | 91.51 (81.35-100.00) | 8.49 (-0.00-18.23) |  |
| Treated | 16017 (6593-25100) | 1250 (-0-2560) | 17266 (7860-25500) |
| Treated (%) | 92.08 (82.27-100.00) | 7.92 (-0.00-17.12) |  |
| Asymptomatic and diagnosed | 2672 (549-4834) | 123 (-0-347) | 2794 (609-4952) |
| Asymptomatic diagnosed (%) | 95.09 (84.42-100.00) | 4.91 (-0.00-13.06) |  |
| Symptomatic and diagnosed | 13345 (5456-21529) | 1127 (-0-2356) | 14472 (6659-22174) |
| Symptomatic and diagnosed (%) | 91.51 (81.35-100.00) | 8.49 (-0.00-18.23) |  |
| Treated/Incidence (%) | 16.68 (12.25-21.06) | 24.98 (10.89-79.81) | 16.57 (12.09-20.96) |
| Asymptomatic and treated (%) | 16.85 (4.75-27.77) | 23.73 (1.36-82.45) | 16.31 (4.42-26.92) |
| Symptomatic and treated (%) | 83.15 (68.34-92.50) | 76.27 (12.60-96.65) | 83.69 (69.00-92.91) |

**Response Table : Summary of number of individuals transitioning to each compartment in the transmission model by risk group from 2024 – 2034** Results presented are relative to the main baseline scenario, where no vaccination programmes were implemented. Transmission probabilities of 2024 to 2034 were kept constant at 2018 levels. Assortativity was changed by +25% from estimated levels from the initial model.

| **Category** | **High risk** | **Low risk** | **Total** |
| --- | --- | --- | --- |
| Screened | 32238 (8551-49679) | 111568 (20951-202991) | 143806 (31830-245724) |
| Screened (%) | 23.69 (13.63-38.58) | 76.31 (45.01-85.02) |  |
| Incidence | 118697 (39925-182525) | 7279 (-0-18124) | 125977 (47955-183222) |
| Incidence (%) | 93.57 (81.38-100.00) | 6.43 (-0.00-18.09) |  |
| Treated | 19719 (7004-31455) | 1092 (-0-2728) | 20811 (8408-31651) |
| Treated (%) | 94.01 (82.34-100.00) | 5.99 (-0.00-16.93) |  |
| Asymptomatic and diagnosed | 3283 (603-6122) | 106 (-0-368) | 3390 (638-6200) |
| Asymptomatic diagnosed (%) | 96.30 (84.83-100.00) | 3.70 (-0.00-12.63) |  |
| Symptomatic and diagnosed | 16436 (5746-28063) | 985 (-0-2561) | 17421 (7000-28063) |
| Symptomatic and diagnosed (%) | 93.57 (81.38-100.00) | 6.43 (-0.00-18.09) |  |
| Treated/Incidence (%) | 16.68 (12.25-21.06) | 31.52 (10.89-85.79) | 16.60 (12.10-21.06) |
| Asymptomatic and treated (%) | 16.85 (4.75-27.77) | 33.76 (1.36-82.86) | 16.44 (4.43-27.25) |
| Symptomatic and treated (%) | 83.15 (68.34-92.50) | 66.24 (12.34-96.53) | 83.56 (68.64-92.87) |

**Response Table : Summary of number of individuals transitioning to each compartment in the transmission model by risk group from 2024 – 2034** Results presented are relative to the main baseline scenario, where no vaccination programmes were implemented. Transmission probabilities of 2024 to 2034 were kept constant at 2018 levels. Assortativity was changed by +50% from estimated levels from the initial model.

**Comment:** The choice of a Negative Binomial distribution to model gonorrhoea case counts is sound, given the over-dispersion typically observed in such data. However, the shape parameter (κ\kappa) is introduced but not fully explained. The paper would benefit from further discussion on how κ\kappaκ was estimated or calibrated and whether alternative distributions (e.g., Poisson or zero-inflated models) were considered and compared.

**Responses:** We thank the reviewer for the comment. We have now explained the shape parameter in the supplementary information after describing the observation process of the data. We have also ran a hypothesis test for overdispersion in our data and found that the annual gonnorhea incidence rates had significant overdispersion at the 95% level (p=0.025), based on Cameron and Trivedi (1990), where year was taken as the explanatory variable of interest and annual gonorrhoea incidence was taken as the dependent variable.

*“We used a negative binomial distribution to characterise the assumed observation process as it was count data, and hypothesis testing indicated that there was significant overdispersion in this dataset [10].* *If* $x$ *∼ NegBinom(*$m$*,* $\kappa$*), with mean* $m$ *and shape parameter* $\kappa$*, then:*

$$\begin{aligned} f_{X}\left( x | m,\kappa\right)=\frac{\Gamma\left( \kappa+x \right)}{x!\Gamma\left( \kappa\right)}\left( \frac{\kappa}{\kappa+m} \right)^{\kappa}\left( \frac{m}{\kappa+m} \right)^{x}\#\left( 8 \right) \end{aligned}$$

*where the shape parameter characterises the level of clustering or heterogeneity in the observation process, and Γ(·) is the Gamma function. Under the Negative-Binomial distribution Var*$\left[ x \right]=m+ \frac{m^{2}}{k}$*.”*

**Comment:** The authors appropriately report 95% credible intervals for key results, but they could further elaborate on how uncertainty in different parameters (e.g., vaccine efficacy, behavioural parameters) contributes to overall uncertainty in the results. A decomposition of uncertainty by source would provide valuable insights into which assumptions or parameters have the most significant impact on model outcomes.

**Responses:** We thank the reviewer for the comment. We have now decomposed the uncertainty of the population health impacts for the various vaccination programmes by iteratively setting each parameter by its median values and all other parameters to vary according to the sampled distribution, and thereafter projecting the vaccination programmes’s impacts over a 10-30year timeframe. As we have varied over 10 parameters and regenerated over 200 vaccination scenarios, we have only described the key results in the main text and append some of the key results here for the reviewer’s perusal.

*“Furthermore, in order to ascertain how the uncertainty in different parameters contribute to the overall uncertainty of the results, we iteratively set each parameter by its median values and all other parameters to vary according to the sampled distribution. Thereafter each the vaccination programmes’s impacts over a 10-30year timeframe was projected, under the 3 separate case serieses”*

*“By decomposing the uncertainty in our projections, we found that epidemiological and behavioural parameters, such as transmission rates at the start, treatment seeking behaviour and screening rates had a large influence on the uncertainty of the estimated impacts for the vaccination programmes, whereas parameters such as the rate of leaving the incubation period, and the probability of symptomatic infection did not contribute to large variations in projected results.”*

Example of SI plot:

***Response figure Projected number of annual diagnosed cases by vaccination programme over 2024–2044.*** *The posterior mean is marked with a square, and the 95% credible interval is indicated by the boundaries of the box. Projections assume that transmission probabilities of 2024 to 2054 were kept constant at 2018 levels. The initial transmission rate of 2004 was set to its estimated median values and all other parameters to vary according to the sampled distribution. 1000 posterior draws were used to generate future projections and calculate programme-specific metrics. The series of boxplots illustrate the annual future number of diagnosed cases under different vaccination strategies compared to a no-vaccination programme baseline. Each column represents a different vaccination programme. Respectively, these are denoted (****A****) vaccination before entry [VbE], (****B****) vaccination on attendance [VoA], (****C****) vaccination on diagnoses with gonorrhoea [VoD], (****D****) vaccination according to risk [VaR], (****E****) VoA and (****F****) VoD strategies by offering vaccinations in these groups to only high sexual activity individuals (VoA[H]), VoD[H]). The radar graph visualizes the impact, efficiency, and uptake of each vaccination programme by 10-year expectation. Metrics are averted cases by percentage (A.P.), averted cases per dose (A.D.), number of vaccinations administered (N.V.), and primary uptake ratio (P.U.). Each sample's metric value is normalized by the maximum and minimum across strategies for visual clarity, points closer to the center are closer to the overall strategy minimum metric score, and the radar border represents the overall maximum score each strategy can achieve.*

**Comment:** The model distinguishes between high and low sexual activity groups but assumes homogeneity within each group. In reality, there might be significant heterogeneity in both behaviour and transmission risks within these groups. A discussion on how heterogeneity within groups could impact model results would be valuable, and considering a more granular approach (e.g., splitting the high-risk group into further subgroups) could yield different insights.

**Responses:** We thank the reviewer for the comment. We agree that there may still be significant heterogeneity in behaviour and transmission risks within the high/low risk groups. We have now added a discussion on this limitation in the discussion section:

*“Our model only incorporated high/low risk sexual activity groups, but assumed homogeneity in behaviours within each group. Therefore, specific and important population subgroups, such as superspreaders, were not accounted for. Potentially high efficiency vaccination strategies, such as targeting those at very high risk, were also not explored, as parameters for these population subgroups were not available for model calibration and scenario analysis.”*

**Comment:** The model assumes a constant population size over time, with individuals entering and exiting the sexually active population. While this is a common assumption in transmission models, it would be useful to acknowledge potential demographic changes (e.g., shifts in the age distribution, migration) and how they might influence the results. Sensitivity analysis based on varying population growth rates could provide further insights into the model's robustness.

**Responses:** We thank the reviewer for the comment and agree that changes in population size can influence our results. We have now conducted sensitivity analysis based on either upward or downward population trends (either of a compounding 1% increase or decrease over time) and found that there were correspondingly increasing/decreasing averted cases but relatively similar averted cases per dose from modelled strategies:

**Response Figure Projected number of annual diagnosed cases and evaluation metrics by vaccination programme over 2024–2034 under hypothetically increasing number of population entrants (1% increase per year)** Posterior means are marked with a square, and the 95% credible intervals are indicated by the boundaries of the box. Model is calibrated to case series of male incidence multiplied by the proportion of reported gonorrhoea cases in the UK MSM population. Projections assume transmission probabilities in 2024 – 2034 were set to decrease following the trend of 2004 to 2018. 1000 posterior draws were used to generate future projections and calculate programme-specific metrics. The series of boxplots illustrate the annual future number of diagnosed cases under different vaccination strategies compared to a no-vaccination programme baseline. Each column represents a different vaccination programme. Respectively, these are denoted (**A**) vaccination before entry [VbE], (**B**) vaccination on attendance [VoA], (**C**) vaccination on diagnoses with gonorrhoea [VoD], (**D**) vaccination according to risk [VaR], (**E**) VoA and (**F**) VoD strategies by offering vaccinations in these groups to only high sexual activity individuals (VoA[H]), VoD[H]).

The radar graph visualizes the impact, efficiency, and uptake of each vaccination programme from 2024 – 2034. Metrics are averted cases by percentage (A.P.), averted cases per dose (A.D.), number of vaccinations administered (N.V.), and primary uptake ratio (P.U.). Each sample's metric value is normalized by the maximum and minimum across strategies for visual clarity, points closer to the center are closer to the overall strategy minimum metric score, and the radar border represents the overall maximum score each strategy can achieve.

**Response Figure Projected number of annual diagnosed cases and evaluation metrics by vaccination programme over 2024–2034 under hypothetically decreasing number of population entrants (-1% increase per year)** Posterior means are marked with a square, and the 95% credible intervals are indicated by the boundaries of the box. Model is calibrated to case series of male incidence multiplied by the proportion of reported gonorrhoea cases in the UK MSM population. Projections assume transmission probabilities in 2024 – 2034 were set to decrease following the trend of 2004 to 2018. 1000 posterior draws were used to generate future projections and calculate programme-specific metrics. The series of boxplots illustrate the annual future number of diagnosed cases under different vaccination strategies compared to a no-vaccination programme baseline. Each column represents a different vaccination programme. Respectively, these are denoted (**A**) vaccination before entry [VbE], (**B**) vaccination on attendance [VoA], (**C**) vaccination on diagnoses with gonorrhoea [VoD], (**D**) vaccination according to risk [VaR], (**E**) VoA and (**F**) VoD strategies by offering vaccinations in these groups to only high sexual activity individuals (VoA[H]), VoD[H]).

The radar graph visualizes the impact, efficiency, and uptake of each vaccination programme from 2024 – 2034. Metrics are averted cases by percentage (A.P.), averted cases per dose (A.D.), number of vaccinations administered (N.V.), and primary uptake ratio (P.U.). Each sample's metric value is normalized by the maximum and minimum across strategies for visual clarity, points closer to the center are closer to the overall strategy minimum metric score, and the radar border represents the overall maximum score each strategy can achieve.

We have now added a discussion on this limitation in the discussion section:

*“A decreasing population size over time would lead to a gradual depletion of susceptibles and lead to gradually lower raw public health impacts for the vaccination programmes, and vice versa, for an increasing population size. However, data on the future population age structure and numbers over the next 10–20 years is currently not available and these scenarios are precluded from our modelling study.”*

**Comment:** The model assumes a constant natural recovery rate for asymptomatic infections. It might be worth exploring if recovery rates vary based on factors like the time since infection, health status, or co-infections. Additionally, it would be helpful to mention if there is any empirical evidence that supports the assumed constant recovery rate, or if any variability in this parameter was tested during sensitivity analysis.

**Responses:** We thank the reviewer for the comment. Unfortunately, data on variations of the natural recovery rate are not available. This would require individual-level information on the epidemiological confounders listed. Evidence on the duration of untreated gonococcal infection (and thereby, the natural recovery rate) is also sparse, there is only data from a single study of 16 women [11], whereas the only similar studies we found in men were decades old and identified infections using techniques that are known to have lower sensitivities. In the referenced studies, it was translated to a natural recovery rate in the range of 45.5 (38.6, 54.4) and 91.3 (77.3, 108.6) days for males and females respectively after bootstrapping of empirical data [12]. This covered our posterior estimate of 70.60 (61.14, 97.33) days for the natural recovery rate. We have conducted an additional sensitivity analysis by varying this rate by $\pm$25, 50% and examined the influence on our results and found that efficacies increased as the natural recovery rate increased:

*“Lastly, we examined the sensitivity of behavioural and epidemiological parameters on our model’s outputs of the prospective vaccination strategies efficiency and raw public health impacts. This was done by varying the parameters (i.e, screening rates, recovery rates, asymptomatic rates, proportion of individuals in high/low risk groups) by* $\pm$*25,50% individually while keeping all other parameters fixed where possible and reprojecting each vaccination strategy through 2024–2034.”*

| **Strategy** | **Averted cases**  **(total)** | **Averted cases**  **(%)** | **Averted cases**  **per dose** | **Number of vaccinations administered** | **Primary**  **uptake ratio** |
| --- | --- | --- | --- | --- | --- |
| VbE | 1558 (863-2117) | 2.42 (1.66-4.35) | 0.005 (0.002-0.006) | 172701 (172701-172701) | 100.00 (100.00-100.00) |
| VoD(H) | 5936 (1920-9080) | 8.83 (5.87-11.84) | 0.22 (0.13-0.30) | 14485 (5392-18299) | 83.87 (78.00-90.42) |
| VoD | 5965 (1921-9157) | 8.87 (5.92-11.85) | 0.18 (0.11-0.24) | 17783 (5667-24148) | 86.61 (79.93-92.09) |
| VoA | 8076 (3219-12387) | 12.25 (7.25-19.49) | 0.08 (0.04-0.13) | 55061 (24732-80740) | 87.81 (83.60-91.22) |
| VoA(H) | 7837 (3183-11850) | 11.92 (7.04-19.03) | 0.23 (0.13-0.31) | 18840 (11421-22816) | 78.93 (72.88-84.83) |
| VaR | 7865 (3184-11906) | 11.96 (7.05-19.05) | 0.20 (0.12-0.27) | 22047 (12002-27912) | 81.77 (75.14-86.84) |

**Response Table Summary of population health impact for various vaccination programmes from 2024 to 2034, with a -50% change in the natural recovery rate.** We report posterior means and accompanying 95% credible intervals. Estimates presented are relative to the baseline scenario of no intervention, with transmission probabilities from 2024 to 2034 kept stable at 2018 levels.

| **Strategy** | **Averted cases**  **(total)** | **Averted cases**  **(%)** | **Averted cases**  **per dose** | **Number of vaccinations administered** | **Primary**  **uptake ratio** |
| --- | --- | --- | --- | --- | --- |
| VbE | 1943 (1112-2585) | 3.88 (3.24-4.35) | 0.006 (0.003-0.007) | 172701 (172701-172701) | 100.00 (100.00-100.00) |
| VoD(H) | 6114 (1920-9545) | 11.98 (7.95-14.99) | 0.28 (0.16-0.38) | 11297 (5392-13641) | 87.49 (83.89-90.72) |
| VoD | 6124 (1921-9559) | 12.00 (7.97-15.01) | 0.24 (0.14-0.32) | 13239 (5667-16554) | 89.20 (85.15-92.22) |
| VoA | 9420 (3533-14186) | 18.60 (10.75-23.58) | 0.10 (0.04-0.16) | 52253 (22566-78177) | 88.89 (84.15-91.74) |
| VoA(H) | 9287 (3492-14090) | 18.34 (10.63-23.38) | 0.30 (0.17-0.40) | 17006 (11006-20542) | 81.02 (75.18-85.65) |
| VaR | 9296 (3493-14094) | 18.36 (10.64-23.39) | 0.27 (0.16-0.35) | 18815 (11772-22841) | 82.76 (76.68-87.40) |

**Response Table Summary of population health impact for various vaccination programmes from 2024 to 2034, with a -25% change in the natural recovery rate.** We report posterior means and accompanying 95% credible intervals. Estimates presented are relative to the baseline scenario of no intervention, with transmission probabilities from 2024 to 2034 kept stable at 2018 levels.

Asymptomatics recover too quickly in the case of +25,50% change in natural recovery rates, leading to unstable estimates:

| **Strategy** | **Averted cases**  **(total)** | **Averted cases**  **(%)** | **Averted cases**  **per dose** | **Number of vaccinations administered** | **Primary**  **uptake ratio** |
| --- | --- | --- | --- | --- | --- |
| VbE | 316 (0-1782) | 11.42 (2.56-22.59) | 0.001 (0.000-0.005) | 172701 (172701-172701) | 100.00 (100.00-100.00) |
| VoD(H) | 182 (0-1428) | 2.53 (0.06-12.91) | 0.05 (0.00-0.27) | 447 (0-2782) | 99.52 (96.24-100.00) |
| VoD | 182 (0-1429) | 2.53 (0.06-12.91) | 0.04 (0.00-0.23) | 505 (0-3156) | 99.57 (96.56-100.00) |
| VoA | 788 (0-4949) | 26.56 (4.30-54.28) | 0.01 (0.00-0.06) | 44372 (10543-72141) | 92.09 (85.53-96.48) |
| VoA(H) | 785 (0-4918) | 26.45 (4.24-54.17) | 0.04 (0.00-0.23) | 10462 (3078-14869) | 88.43 (80.20-94.45) |
| VaR | 785 (0-4919) | 26.45 (4.24-54.17) | 0.04 (0.00-0.22) | 10498 (3078-14869) | 88.47 (80.20-94.56) |

**Response Table Summary of population health impact for various vaccination programmes from 2024 to 2034, with a +25% change in the natural recovery rate.** We report posterior means and accompanying 95% credible intervals. Estimates presented are relative to the baseline scenario of no intervention, with transmission probabilities from 2024 to 2034 kept stable at 2018 levels.

| **Strategy** | **Averted cases**  **(total)** | **Averted cases**  **(%)** | **Averted cases**  **per dose** | **Number of vaccinations administered** | **Primary**  **uptake ratio** |
| --- | --- | --- | --- | --- | --- |
| VbE | 316 (0-1782) | 11.07 (20.97-22.68) | 0.001 (0.000-0.005) | 172701 (172701-172701) | 100.00 (100.00-100.00) |
| VoD(H) | 182 (0-1428) | 2.53 (0.00-12.91) | 0.05 (0.00-0.27) | 447 (-0-2782) | 99.52 (96.24-100.00) |
| VoD | 182 (0-1429) | 2.53 (0.00-12.91) | 0.04 (0.00-0.23) | 505 (-0-3156) | 99.57 (96.56-100.00) |
| VoA | 788 (0-4949) | 24.64 (0.00-54.28) | 0.01 (0.00-0.06) | 44372 (10543-72141) | 92.09 (85.53-96.48) |
| VoA(H) | 785 (0-4918) | 24.55 (0.00-54.17) | 0.04 (0.00-0.23) | 10462 (3078-14869) | 88.43 (80.20-94.45) |
| VaR | 785 (0-4919) | 24.54 (0.00-54.17) | 0.04 (0.00-0.22) | 10498 (3078-14869) | 88.47 (80.20-94.56) |

**Response Table Summary of population health impact for various vaccination programmes from 2024 to 2034, with a +50% change in the natural recovery rate.** We report posterior means and accompanying 95% credible intervals. Estimates presented are relative to the baseline scenario of no intervention, with transmission probabilities from 2024 to 2034 kept stable at 2018 levels.

**Comment:** The paper presents a screening rate equation (Equation 2 in the supplementary), which assumes a fixed relationship between the high- and low-risk groups (ω\omega). However, screening rates could vary over time based on factors like public health campaigns, changes in healthcare access, or risk perception. Modelling time-varying screening rates, or at least discussing their potential influence, would enhance the realism of the model.

**Responses:** We thank the reviewer for the comment. We agree that differences in screening rates may change over time and our model coarsely captures the average screening rate over the period where incidence rates were available for model calibration. As our output is primarily focused on projecting the potential future impacts of the vaccination programmes, it is difficult to also ascertain the changes in public health campaigns, changes in healthcare access, or risk perception over the future 10 to 20-year time horizon. We therefore relied on a constant, risk-group specific screening rate for these projections. We have discussed their limitations in the discussion section:

*“As our output is primarily focused on projecting the potential future impacts of the vaccination programmes, it is difficult to forecast and incorporate changes in public health campaigns, changes in healthcare access, or risk perception, which may influence screening rates over the future 10 to 20-year time horizon. We therefore relied on a constant, risk-group specific screening rate for these projections. Hypothetically, increases in screening rates due to intensified public health campaigns may increase the public health impacts of strategies such as VoA and VoD, by provision of vaccination to more individuals attending sexual health clinics.”*

**Comment:** The manuscript mentions the use of proxies for MSM incidence due to unavailable data (Supplementary Information, Section 2.1). While this approach is reasonable, it would be beneficial to provide more detail on how uncertainty due to these data limitations is incorporated into the model. Specifically, if the authors explored the impact of different proxy assumptions on the model outputs, it would demonstrate the robustness of the conclusions to data-related uncertainty.

**Responses:** We thank the reviewer for the comment. We have proxied MSM incidence by (**1**) male incidence less the female incidence of gonorrhoea (**2**) the male incidence of gonorrhoea, i.e the upper bound to the annual gonorrhoea MSM incidence (**3**) the male incidence multiplied by the proportion of reported gonorrhoea cases in the MSM population in other study settings. These respectively formed the central, upper and lower bound of MSM incidence in Singapore. In the SI, we have calibrated our model to each of these incidence rates, projected the future incidences based on constant or linearly trending transmission rates over 2024 – 2034 and assessed the vaccination programmes’ public health impacts over these horizons. We apologise for omitting a summary of these sensitivity analyses and results in the main text and have amended the text as below:

“As actual breakdowns of annual gonorrhoea MSM incidence were not available in the study setting, we assumed that this was proxied by (**1**) male incidence less the female incidence of gonorrhoea (**2**) the male incidence of gonorrhoea, i.e the upper bound to the annual gonorrhoea MSM incidence (**3**) the male incidence multiplied by the proportion of reported gonorrhoea cases in the MSM population in other study settings (See supplementary information, Table S1). Raw annual gonorrhoea incidence was obtained from the communicable disease surveillance system of Singapore from 2004-2020 [1] and excluded observations in 2019, 2020 due to COVID-19. We calibrated our model under each scenario and reported the first scenario as our main results.”

“We separately calibrated the transmission models based on the different proxies of MSM gonorrhea incidence (see **Data** above). We projected the future incidences based on constant or linearly trending transmission rates over 2024 – 2034 and assessed the influence of these separate models on vaccination programmes’ public health impacts over the projected time horizon.”

*“Simulating a more optimistic baseline case where gonorrhoea transmission probabilities decreased in 2024 to 2034 following the trend from 2004-2014, and/or calibration of the transmission model to alternative time series of gonorrhoea incidence in MSM, did not significantly affect our results on vaccination programme efficiency and efficacy (See supplementary information section 5).”*

*“However, in our exploration of the public health impacts of these vaccination programmes, we did not study the cost-effectiveness of these strategies, which departed from prior modelling work done in settings of historical increasing incidence settings [3], due to the lack of publicly available cost data on gonorrhoea treatment. We did not calibrate our model to actual gonorrhoea incidence in MSM populations, as these were not available. Instead, we calibrated our model to 3 alternative epidemiological time series which could realistically proxy epidemiological patterns in MSM populations across the years – across these different scenarios, we found that our results on vaccination programme impact and efficiency did not vary significantly.”*

We also append the visualizations of the sensitivity analyses from the SI below:

**5.1 Projections of gonorrhoea epidemiology** **under decreasing transmission probability from 2024 – 2034, calibrated to case series of male incidence less the female incidence of gonorrhoea**

**Figure S4 Projected number of annual diagnosed cases and evaluation metrics by vaccination programme over 2024–2034** The posterior mean is marked with a square, and the 95% credible interval is indicated by the boundaries of the box. Model is calibrated to case series of the male incidence of gonorrhea. Projections assume that transmission probabilities decrease linearly in 2024 – 2034 following the trend from 2004 to 2018. 1000 posterior draws were used to generate future projections and calculate programme-specific metrics. The series of boxplots illustrate the annual future number of diagnosed cases under different vaccination strategies compared to a no-vaccination programme baseline. Each column represents a different vaccination programme. Respectively, these are denoted (**A**) vaccination before entry [VbE], (**B**) vaccination on attendance [VoA], (**C**) vaccination on diagnoses with gonorrhoea [VoD], (**D**) vaccination according to risk [VaR], (**E**) VoA and (**F**) VoD strategies by offering vaccinations in these groups to only high sexual activity individuals (VoA[H]), VoD[H]).

The radar graph visualizes the impact, efficiency, and uptake of each vaccination programme from 2024 – 2034. Metrics are averted cases by percentage (A.P.), averted cases per dose (A.D.), number of vaccinations administered (N.V.), and primary uptake ratio (P.U.). Each sample's metric value is normalized by the maximum and minimum across strategies for visual clarity, points closer to the center are closer to the overall strategy minimum metric score, and the radar border represents the overall maximum score each strategy can achieve.

**5.2 Results of model calibrated to the case series of male incidence of gonorrhea, with projected transmission probabilities in 2024 – 2034 set to that of 2018**

**Figure S5 Projected number of annual diagnosed cases and evaluation metrics by vaccination programme over 2024–2034**

The posterior mean is marked with a square, and the 95% credible interval is indicated by the boundaries of the box. Model is calibrated to case series of the male incidence of gonorrhea. Projections assume that transmission probabilities remain stable in 2024 – 2034 at 2018 levels. 1000 posterior draws were used to generate future projections and calculate programme-specific metrics. The series of boxplots illustrate the annual future number of diagnosed cases under different vaccination strategies compared to a no-vaccination programme baseline. Each column represents a different vaccination programme. Respectively, these are denoted (**A**) vaccination before entry [VbE], (**B**) vaccination on attendance [VoA], (**C**) vaccination on diagnoses with gonorrhoea [VoD], (**D**) vaccination according to risk [VaR], (**E**) VoA and (**F**) VoD strategies by offering vaccinations in these groups to only high sexual activity individuals (VoA[H]), VoD[H]).

The radar graph visualizes the impact, efficiency, and uptake of each vaccination programme from 2024 – 2034. Metrics are averted cases by percentage (A.P.), averted cases per dose (A.D.), number of vaccinations administered (N.V.), and primary uptake ratio (P.U.). Each sample's metric value is normalized by the maximum and minimum across strategies for visual clarity, points closer to the center are closer to the overall strategy minimum metric score, and the radar border represents the overall maximum score each strategy can achieve

**5.3 Results of model calibrated to the case series of male incidence of gonorrhea, with projected transmission probabilities in 2024 – 2034 set to decrease following the trend of 2004 to 2018**

**Figure S6 Projected number of annual diagnosed cases and evaluation metrics by vaccination programme over 2024–2034** Posterior means are marked with a square, and the 95% credible interval is indicated by the boundaries of the box. Model is calibrated to case series of the male incidence of gonorrhea. Projections assume that transmission probabilities decrease in 2024 – 2034 following trends from 2004 to 2018. 1000 posterior draws were used to generate future projections and calculate programme-specific metrics. The series of boxplots illustrate the annual future number of diagnosed cases under different vaccination strategies compared to a no-vaccination programme baseline. Each column represents a different vaccination programme. Respectively, these are denoted (**A**) vaccination before entry [VbE], (**B**) vaccination on attendance [VoA], (**C**) vaccination on diagnoses with gonorrhoea [VoD], (**D**) vaccination according to risk [VaR], (**E**) VoA and (**F**) VoD strategies by offering vaccinations in these groups to only high sexual activity individuals (VoA[H]), VoD[H]).

The radar graph visualizes the impact, efficiency, and uptake of each vaccination programme from 2024 – 2034. Metrics are averted cases by percentage (A.P.), averted cases per dose (A.D.), number of vaccinations administered (N.V.), and primary uptake ratio (P.U.). Each sample's metric value is normalized by the maximum and minimum across strategies for visual clarity, points closer to the center are closer to the overall strategy minimum metric score, and the radar border represents the overall maximum score each strategy can achieve.

**5.4 Results of model calibrated to the case series of male incidence multiplied by the proportion of reported gonorrhoea cases in the UK MSM population, with projected transmission probabilities in 2024 – 2034 set to that of 2018**

**Figure S7 Projected number of annual diagnosed cases and evaluation metrics by vaccination programme over 2024–2034** Posterior means are marked with a square, and the 95% credible interval are indicated by the boundaries of the box. Model is calibrated to case series of male incidence multiplied by the proportion of reported gonorrhoea cases in the UK MSM population. Projections assume that where transmission probabilities remain stable in 2024 – 2034 at 2018 levels. 1000 posterior draws were used to generate future projections and calculate programme-specific metrics. The series of boxplots illustrate the annual future number of diagnosed cases under different vaccination strategies compared to a no-vaccination programme baseline. Each column represents a different vaccination programme. Respectively, these are denoted (**A**) vaccination before entry [VbE], (**B**) vaccination on attendance [VoA], (**C**) vaccination on diagnoses with gonorrhoea [VoD], (**D**) vaccination according to risk [VaR], (**E**) VoA and (**F**) VoD strategies by offering vaccinations in these groups to only high sexual activity individuals (VoA[H]), VoD[H]).

The radar graph visualizes the impact, efficiency, and uptake of each vaccination programme from 2024 – 2034. Metrics are averted cases by percentage (A.P.), averted cases per dose (A.D.), number of vaccinations administered (N.V.), and primary uptake ratio (P.U.). Each sample's metric value is normalized by the maximum and minimum across strategies for visual clarity, points closer to the center are closer to the overall strategy minimum metric score, and the radar border represents the overall maximum score each strategy can achieve.

**5.5 Results of model calibrated to the case series of male incidence multiplied by the proportion of reported gonorrhoea cases in the UK MSM population, with projected transmission probabilities in 2024 – 2034 set to decrease following the trend of 2004 to 2018**

**Figure S8 Projected number of annual diagnosed cases and evaluation metrics by vaccination programme over 2024–2034** Posterior means are marked with a square, and the 95% credible intervals are indicated by the boundaries of the box. Model is calibrated to case series of male incidence multiplied by the proportion of reported gonorrhoea cases in the UK MSM population. Projections assume transmission probabilities in 2024 – 2034 were set to decrease following the trend of 2004 to 2018. 1000 posterior draws were used to generate future projections and calculate programme-specific metrics. The series of boxplots illustrate the annual future number of diagnosed cases under different vaccination strategies compared to a no-vaccination programme baseline. Each column represents a different vaccination programme. Respectively, these are denoted (**A**) vaccination before entry [VbE], (**B**) vaccination on attendance [VoA], (**C**) vaccination on diagnoses with gonorrhoea [VoD], (**D**) vaccination according to risk [VaR], (**E**) VoA and (**F**) VoD strategies by offering vaccinations in these groups to only high sexual activity individuals (VoA[H]), VoD[H]).

The radar graph visualizes the impact, efficiency, and uptake of each vaccination programme from 2024 – 2034. Metrics are averted cases by percentage (A.P.), averted cases per dose (A.D.), number of vaccinations administered (N.V.), and primary uptake ratio (P.U.). Each sample's metric value is normalized by the maximum and minimum across strategies for visual clarity, points closer to the center are closer to the overall strategy minimum metric score, and the radar border represents the overall maximum score each strategy can achieve.

**Comment:** The manuscript provides a good analysis of how vaccine efficacy and duration of protection influence outcomes. However, there is limited discussion on how realistic these assumptions are. Are the vaccine characteristics assumed in the model (e.g., 31% efficacy, 4-year duration of protection) based on real-world clinical data, or are they optimistic estimates? Including a section on how these values compare to available empirical data on vaccine performance (both for meningococcal and potential gonorrhoea vaccines) would ground the results in reality.

**Responses:** We thank the reviewer for the comment. The estimates were based on real world clinical data and have been justified in the Vaccine profile and uptake section of the manuscript:

*“A retrospective observational case-control study confirmed that the N. meningitidis serogroup B OMV vaccine (MeNZB [GSK]) provided a 31% (95% CrI 21–39) efficacy against gonorrhoea with a 3-dose program [13]. Subsequent studies provided further genetic evidence that the anti-gonococcal antibodies induced by MeNZB can offer additional cross-protection against gonorrhoea [14]. Furthermore, studies from the U.S., Canada, and Australia have reported that 4CMenB, an alternative vaccine option for Gonorrhoea, can be more effective than MeNZB, with efficacies ranging between 33% and 46% [15]. However, recent study also suggested a more conservative 22% efficacy of 4CMenB vaccine in gonorrhoea prevention [7].”*

*“We used the efficacy of 4CMenB at 31% (95% CrI: 21–39) as the primary vaccine profile to run simulations of each vaccination programme [13].”*

*“While the duration of protection of 4CMenB against gonorrhoea is still unclear, observations reported high human complement serum bactericidal antibody assay (hSBA) tilters against fHbp, NadA and PorA on adolescents for 18–36 months [16], young adults for 4 to 7.5 years [17] after 2-dose of primary 4CMenB uptake, suggesting the vaccination with 4CMenb can confer humoral immunity against gonorrhoea for around 4 years, which we used as our central estimate.”*

**Comment:** The authors could enhance their interpretation by comparing their results to similar models used for other sexually transmitted infections (e.g., HPV, chlamydia). This comparison would provide context to the findings, particularly regarding the efficiency of different vaccination strategies in low-prevalence settings. Such a comparison might also highlight generalizable lessons or key differences in vaccination program design across diseases.

**Responses:** We thank the reviewer for the comment and have supplemented our discussion with results from models of other STIs:

***“****Our work corroborates with literature on the potential public health benefits of vaccination programmes of sexually transmitted infections. Gonorrhea vaccination in settings of increasing incidence are projected to be cost-effective when administered according to risk in sexual health clinics [3]. Pre-emptive vaccination is expected to reduce the likelihood of monkeypox outbreaks in the same study setting, especially in targeting of dense and well-connected sexual contact networks [18]. Whereas vaccination for human papillomavirus can reduce the risk of cervical cancer with increasing improvement in equality in cervical cancer incidence in the future [19].****”***

**Comment:** The model is projected over a 10-year period. This time frame may not fully capture the longer-term effects of vaccination programs, especially for interventions like adolescent vaccination (VbE) that may have delayed benefits. Extending the model to 20 or 30 years could provide insights into whether long-term herd immunity can be achieved or if the benefits of certain strategies increase over time.

**Responses:** We thank the reviewer for the comment and have now extended the projections to a 20- to 30-year horizon as an additional sensitivity analysis. We have found that the hypothetical reductions in incidence tapers off for VoA, VoA(H) and VaR around the 20 year mark and remains at a suppressed level, assuming a constant rate of transmission at 2018 levels:

“*Furthermore, in order to ascertain how the uncertainty in different parameters contribute to the overall uncertainty of the results, we iteratively set each parameter by its median values and all other parameters to vary according to the sampled distribution. Thereafter each the vaccination programmes’s impacts over a 10-30year timeframe was projected*”

*“Simulating our results over a 30-year instead of 20-year timeframe also indicated that the hypothetical reductions in incidence can be maintained for VoA, VoA(H) and VaR around the 20 year mark and remains at a suppressed level, assuming a constant rate of transmission at 2018 levels.”*

***Response Figure: Projected number of annual diagnosed cases by vaccination programme over 2024–2044*** *The posterior mean is marked with a square, and the 95% credible interval is indicated by the boundaries of the box. Projections assume that Transmission probabilities of 2024 to 2034 were kept constant at 2018 levels. 1000 posterior draws were used to generate future projections and calculate programme-specific metrics. The series of boxplots illustrate the annual future number of diagnosed cases under different vaccination strategies compared to a no-vaccination programme baseline. Each column represents a different vaccination programme. Respectively, these are denoted (****A****) vaccination before entry [VbE], (****B****) vaccination on attendance [VoA], (****C****) vaccination on diagnoses with gonorrhoea [VoD], (****D****) vaccination according to risk [VaR], (****E****) VoA and (****F****) VoD strategies by offering vaccinations in these groups to only high sexual activity individuals (VoA[H]), VoD[H]).*

*The radar graph visualizes the impact, efficiency, and uptake of each vaccination programme by 10-year expectation. Metrics are averted cases by percentage (A.P.), averted cases per dose (A.D.), number of vaccinations administered (N.V.), and primary uptake ratio (P.U.). Each sample's metric value is normalized by the maximum and minimum across strategies for visual clarity, points closer to the center are closer to the overall strategy minimum metric score, and the radar border represents the overall maximum score each strategy can achieve.*

**Comment:** While the authors present credible intervals for key results, some figures could benefit from a clearer representation of uncertainty. For example, Figures 2 and 3 use box plots and radar graphs to display outcomes, but adding additional uncertainty visualization (such as density plots or violin plots) might make the distributions of outcomes more apparent.

**Responses:** We thank the reviewer for the comment, and have now added density plots in the supplementary information to accompany each radar graph which were presented in the main text. As these appear throughout the SI for other results, we provide an example of the visualizations which appear in the SI below:

***Response Figure: Projected number of annual diagnosed cases by vaccination programme over 2024–2034*** *The posterior densities of averted cases by percentage, averted cases per dose, number of vaccinations and primary uptake ratio. Projections assume that transmission probabilities of 2024 to 2034 were kept constant at 2018 levels. 1000 posterior draws were used to generate future projections and calculate programme-specific metrics. Each column represents a different vaccination programme. Respectively, these are denoted (****A****) vaccination before entry [VbE], (****B****) vaccination on attendance [VoA], (****C****) vaccination on diagnoses with gonorrhoea [VoD], (****D****) vaccination according to risk [VaR], (****E****) VoA and (****F****) VoD strategies by offering vaccinations in these groups to only high sexual activity individuals (VoA[H]), VoD[H]).*

**Comment:** It appears that the table currently labelled Table S3 in the supplementary file, which presents "Fitted transmission parameters: notation, definition, prior distribution, parameter bounds, and posterior estimates," should actually be Table S4 based on the content flow. It seems that there are other tables (e.g., Tables S1, S2) preceding it. Therefore, the numbering of Table S3 should be revised to Table S4, and the subsequent tables should be renumbered accordingly to maintain consistency.

**Responses:** We thank the reviewer for the comment. Each table and figure has now been renumbered to ensure consistency. As the changes are minor but made throughout the SI, please refer to the SI itself for these changes.

**Comment:** The graphical representation of vaccination strategy impact (Figure 2) is effective. However, some of the efficiency metrics (e.g., averted cases per dose) could benefit from a clearer legend and explanation in the figure captions. Additionally, radar charts are visually appealing but may obscure some important nuances in the data.

**Responses:** We thank the reviewer for the comment, and have now added density plots in the supplementary information to accompany each radar graph in the main text for clarity (Please see above density plot as the example).

**Editorial comments**

**Comment:** We ask every co-author listed on the manuscript to fill in a contributing author statement, making sure to declare all competing interests. If any of the co-authors have not filled in the statement, we will remind them to do so when the paper is revised. If all statements are not completed in a timely fashion this could hold up the re-review process. If new competing interests are declared later in the revision process, this may also hold up the submission. Should there be a problem getting one of your co-authors to fill in a statement we will be in contact. Please do not add or remove authors without first discussing this with the handling editor. You can see our competing interests policy here: <http://journals.plos.org/plosmedicine/s/competing-interests>.

**Response**: We have now filled the ICMJE conflict of interest file for all authors and attached this in the resubmission.

**Comment:** Please upload any figures associated with your paper as individual TIF or EPS files with 300dpi resolution at resubmission; please read our figure guidelines for more information on our requirements: <http://journals.plos.org/plosmedicine/s/figures>. While revising your submission, please upload your figure files to the PACE digital diagnostic tool, <https://pacev2.apexcovantage.com/>. PACE helps ensure that figures meet PLOS requirements. To use PACE, you must first register as a user. Then, login and navigate to the UPLOAD tab, where you will find detailed instructions on how to use the tool. If you encounter any issues or have any questions when using PACE, please email us at [PLOSMedicine@plos.org](mailto:PLOSMedicine@plos.org).

**Response**: We have now uploaded the main text figures as TIF files with 300dpi.

**Comment:** Abstract: Please structure your abstract using the PLOS Medicine headings (Background, Methods and Findings, Conclusions). Please combine the Methods and Findings sections into one section.

**Response**: We have now changed the abstract to the required format:

*“****Background*** *Gonorrhoea ranks the second most common sexually transmitted infections notified in Singapore in 2023, despite decreasing incidence over the years. Evidence suggests that the four-component serogroup B meningococcal (4CMenB) vaccine designed against Neisseria meningitidis can offer partial cross-protection against gonorrhoea. This has generated interest in using 4CMenB for the purpose of staving gonorrhoea transmission. Here, we explored the efficacy of potential gonorrhoea vaccination strategies in the context of historically declining gonorrhoea incidence.*

***Methods and Findings*** *We employed an integrated transmission-dynamic model, calibrated using Bayesian methods to local surveillance data to understand the potential public health impact of 4CMenB in reducing gonorrhea transmission in men who have sex with men (MSM) in Singapore. We explored the efficacy of implementing 6 vaccination programmes, (****1****) offering vaccination to all male adolescents in schools (vaccination before entry [VbE]), (****2****) offering vaccination to individuals attending sexual health clinics for testing (vaccination on attendance [VoA]); (****3****) offering vaccination on individuals attending sexual health clinics and were diagnosed with Gonorrhoea (vaccination on diagnosis [VoD]); or (****4****) vaccination according to risk (VaR), by offering vaccination to patients who were diagnosed with gonorrhoea plus individuals who tested negative but report having more than five sexual partners per year. We further examined how altering (****5****) VoA and (****6****) VoD strategies changed if the strategies only targeted high risk groups (VoA(H), VoD(H)). We assessed efficacy by examining vaccination impact relative to the baseline case of no vaccination and during when behavioural parameters (sexual risk behaviour and screening rates) were held constant. We further ascertained the effects of varying vaccine uptake (10%, 33%, 100%), vaccine efficacy (22%, 31%, 47%) and duration of protection (1.5, 4, 7.5 years) on the effectiveness of each vaccination strategy.*

*In a hypothetical 10-year vaccination programme, VbE had 14.18% of MSM Gonorrhoea cases averted over the intervention time. VoA had the highest protective impact on the MSM population with 40.26% averted cases (95% CrI 18.32%-52.57%) but required more vaccine doses than any other strategy, whereas VoD had a smaller impact (12.04% averted cases (95% CrI 7.12%-15.00%)) but three times efficient higher than that of VoA in terms of averted cases per dose. VoA(H) and VoD(H) improved the efficiency of VoA and VoD strategies by increasing averted cases per dose to 0.22 and 0.24 respectively, but conferred similar protective effects as VoA (VoA(H): 40.10% averted cases (95% CrI 18.14%-52.55%)) and VoD (VoD(H): 12.04% averted cases (95% CrI 7.12%-15.00%)) respectively. VaR (40.10% averted cases (95% CrI 18.14%-52.55%)) had almost the same impact as VoA but required fewer doses administered than VoA, with averted cases per dose at 0.21 (95% CrI: 0.12-0.27). Sensitivity analyses which varied vaccine duration of protection and efficacy indicated that VaR had the greatest public health impact with the highest number of averted cases per dose for vaccines of any efficacy or duration of protection (or both), although VoD and VoD(H) saved more vaccine resource and had the highest number averted MSM cases per dose for highly protective vaccines of long protection.*

***Conclusions*** *Vaccination of MSM against gonorrhoea according to risk in sexual health clinics in Singapore can be considered. Development of gonorrhoea-specific vaccines should prioritise maximising efficacy over duration of protection.”*

**Comment:** At this stage, we ask that you include a short, non-technical Author Summary of your research to make findings accessible to a wide audience that includes both scientists and non-scientists. The Author Summary should immediately follow the Abstract in your revised manuscript. This text is subject to editorial change and should be distinct from the scientific abstract. Ideally each sub-heading should contain 2-3 single sentence, concise bullet points containing the most salient points from your study. In the final bullet point of 'What Do These Findings Mean?', please include the main limitations of the study in non-technical language. Please see our author guidelines for more information: <https://journals.plos.org/plosmedicine/s/revising-your-manuscript#loc-author-summary> (Please remove the ‘Research in context’ section.)

**Response**: We have now added a short non-technical summary immediately following the abstract. We have removed the research in context section:

***“Author Summary***

- *This article explores the efficacy of potential gonorrhoea vaccination strategies in the context of historically declining gonorrhoea incidence, using an integrated transmission-dynamic model, calibrated using Bayesian methods to local surveillance data*
- *Vaccination to high risk groups was projected to have the greatest public health impact with the highest number of averted cases per dose for vaccines of any efficacy or duration of protection (or both), but vaccination after diagnoses, or vaccination after diagnoses in high risk group individuals saved more vaccine resource and had the highest number averted cases per dose for highly protective vaccines of long protection. Gonorrhoea-specific vaccines can confer greater public health impacts if they prioritise maximising efficacy over the duration of protection.*
- ***What Do These Findings Mean?*** *Vaccination of MSM against gonorrhoea according to risk in sexual health clinics can be potentially efficient and confer high public health impacts, in settings of declining gonorrhoea incidence, such as Singapore.”*

**Comment:** Please express the main results with 95% CIs as well as p values. When reporting p values please report as p<0.001 and where higher as the exact p value p=0.002, for example. Throughout, suggest reporting statistical information as follows to improve clarity for the reader "22% (95% CI [13%,28%]; p</=)". Please be sure to define all numerical values at first use.

**Response**: We have expressed all results in credible intervals, as Bayesian methods were used to calibrate the models and characterise associated uncertainty.

**Comment:** Please include page numbers and line numbers in the manuscript file. Use continuous line numbers (do not restart the numbering on each page).

**Response**: We have now included page and line numbers in the manuscript file.

**Comment:** Please cite the reference numbers in square brackets. Citations should precede punctuation.

**Response**: We have now provided the reference numbers in square brackets.

**Comment:** Please provide titles and legends for all figures and tables (including those in Supporting Information files).

**Response**: We have now provided titles and legends for all figures and tables.

**Comment:** Please define all abbreviations used in each figure/table (including those in Supporting Information files).

**Response**: We have defined all abbreviations used in each figure/table

**Comment:** Please consider avoiding the use of red and green in order to make your figure more accessible to those with color blindness.

**Response**: We have minimized the use of red and green in order to make the figure more accessible to those with color blindness.

**Comment:** Please note that supplementary material will be posted as supplied by the authors. Therefore, please amend it according to the relevant comments outlined here.

**Response**: We have noted this point.

**Comment:** Please cite your Supporting Information as outlined here: <https://journals.plos.org/plosmedicine/s/supporting-information>

**Response**: We have cited the SI accordingly.

**Comment:** PLOS uses the numbered citation (citation-sequence) method and first six authors, et al.

**Response**: We have cited the references accordingly.

**Comment:** Please ensure that journal name abbreviations match those found in the National Center for Biotechnology Information (NCBI) databases (<http://www.ncbi.nlm.nih.gov/nlmcatalog/journals>), and are appropriately formatted and capitalised.

**Response**: We have cited the references accordingly.

**Comment:** Where website addresses are cited, please include the complete URL and specify the date of access (e.g. [accessed: 12/06/2023]).

**Response**: We have cited the references accordingly.

**Comment:** Please also see <https://journals.plos.org/plosmedicine/s/submission-guidelines#loc-references> for further details on reference formatting.

**Response**: We have cited the references accordingly.

**Comment:** If pertinent, please provide a diagram that shows the model structure, including how the natural history of the disease is represented, the process and determinants of disease acquisition, and how the putative intervention could affect the system.

**Response**: We have provided this diagram in the main text and a further elaboration in the SI.

**Comment:** Please provide a complete list of model parameters, including clear and precise descriptions of the meaning of each parameter, together with the values or ranges for each, with justification or the primary source cited and important caveats about the use of these values noted.

**Response**: We have provided this in the SI tables.

**Comment:** Please provide a clear statement about how the model was fitted to the data, including goodness-of-fit measure, the numerical algorithm used, which parameter varied, constraints imposed on parameter values, and starting conditions.

**Response**: We have provided this in the SI.

**Comment:** For uncertainty analyses, please state the sources of uncertainties quantified and not quantified [can include parameter, data, and model structure].

**Response**: We have provided this in the SI.

**Comment:** Please provide sensitivity analyses to identify which parameter values are most important in the model. Uncertainty estimates seek to derive a range of credible results on the basis of an exploration of the range of reasonable parameter values. The choice of method should be presented and justified.

**Response**: We have provided this in the SI with a full sensitivity analysis for the parameters where possible.

**Comment:** Please discuss the scientific rationale for the choice of model structure and identify points where this choice could influence conclusions drawn. Please also describe the strength of the scientific basis underlying the key model assumptions.

**Response**: We have provided this in the main text and SI.

**Comment:** For studies that develop a prediction model or evaluate its performance, please ensure that the study is reported according to the TRIPOD statement (<https://www.equator-network.org/reporting-guidelines/tripod-statement>) and include the completed checklist as Supporting Information. Please add the following statement, or similar, to the Methods: "This study is reported as per the Transparent Reporting of a Multivariable Prediction Model for Individual Prognosis Or Diagnosis (TRIPOD) statement (S1 Checklist)." For studies using machine learning, please use the TRIPOD-AI checklist. When completing the checklist, please use section and paragraph numbers, rather than page numbers.

**Response**: We have noted this comment. This paper does not have a prediction model.

Reference:

1.	MOH | Communicable Diseases Surveillance in Singapore 2019-2020 [Internet]. [cited 2024 May 14]. Available from: https://www.moh.gov.sg/resources-statistics/reports/communicable-diseases-surveillance-in-singapore-2019-2020

2.	Unemo M, Lahra MM, Escher M, Eremin S, Cole MJ, Galarza P, et al. WHO global antimicrobial resistance surveillance for Neisseria gonorrhoeae 2017–18: a retrospective observational study. Lancet Microbe. 2021 Nov 1;2(11):e627–36.

3.	Whittles LK, Didelot X, White PJ. Public health impact and cost-effectiveness of gonorrhoea vaccination: an integrated transmission-dynamic health-economic modelling analysis. Lancet Infect Dis. 2022 Jul 1;22(7):1030–41.

4.	Unemo M, Seifert HS, Hook EW, Hawkes S, Ndowa F, Dillon JAR. Gonorrhoea. Nat Rev Dis Primer. 2019 Nov 21;5(1):1–23.

5.	Unemo M, Shafer WM. Antimicrobial Resistance in Neisseria gonorrhoeae in the 21st Century: Past, Evolution, and Future. Clin Microbiol Rev. 2014 Jul;27(3):587–613.

6.	Sinka K. The global burden of sexually transmitted infections. Clin Dermatol. 2024 Mar 1;42(2):110–8.

7.	Molina JM, Bercot B, Assoumou L, Rubenstein E, Algarte-Genin M, Pialoux G, et al. Doxycycline prophylaxis and meningococcal group B vaccine to prevent bacterial sexually transmitted infections in France (ANRS 174 DOXYVAC): a multicentre, open-label, randomised trial with a 2 × 2 factorial design. Lancet Infect Dis. 2024 May;S1473309924002366.

8.	Abara WE, Kirkcaldy RD, Bernstein KT, Galloway E, Learner ER. Effectiveness of MenB-4C Vaccine Against Gonorrhea: A Systematic Review and Meta-analysis. J Infect Dis. 2024 Jul 31;jiae383.

9.	Garnett GP, Mertz KJ, Finelli L, Levine WC, St Louis ME. The transmission dynamics of gonorrhoea: modelling the reported behaviour of infected patients from Newark, New Jersey. Philos Trans R Soc B Biol Sci. 1999 Apr 29;354(1384):787–97.

10.	Cameron AC, Trivedi PK. Regression-based tests for overdispersion in the Poisson model. J Econom. 1990 Dec 1;46(3):347–64.

11.	Stupiansky NW, Van Der Pol B, Williams JA, Weaver B, Taylor SE, Fortenberry JD. The Natural History of Incident Gonococcal Infection in Adolescent Women. Sex Transm Dis. 2011 Aug;38(8):750.

12.	Kreisel KM, Weston EJ, St. Cyr SB, Spicknall IH. Estimates of the Prevalence and Incidence of Chlamydia and Gonorrhea Among US Men and Women, 2018. Sex Transm Dis. 2021 Apr;48(4):222.

13.	Effectiveness of a group B outer membrane vesicle meningococcal vaccine against gonorrhoea in New Zealand: a retrospective case-control study - ClinicalKey [Internet]. [cited 2024 May 20]. Available from: https://www-clinicalkey-com.remotexs.ntu.edu.sg/#!/content/playContent/1-s2.0-S0140673617314496?returnurl=null&referrer=null

14.	Semchenko EA, Tan A, Borrow R, Seib KL. The Serogroup B Meningococcal Vaccine Bexsero Elicits Antibodies to Neisseria gonorrhoeae. Clin Infect Dis. 2019 Sep 13;69(7):1101–11.

15.	Ladhani SN, White PJ, Campbell H, Mandal S, Borrow R, Andrews N, et al. Use of a meningococcal group B vaccine (4CMenB) in populations at high risk of gonorrhoea in the UK. Lancet Infect Dis [Internet]. 2024 Mar 20 [cited 2024 May 23];0(0). Available from: https://www-thelancet-com.remotexs.ntu.edu.sg/journals/laninf/article/PIIS1473-3099(24)00031-8/fulltext

16.	Santolaya ME, O’Ryan M, Valenzuela MT, Prado V, Vergara RF, Muñoz A, et al. Persistence of antibodies in adolescents 18−24 months after immunization with one, two, or three doses of 4CMenB meningococcal serogroup B vaccine. Hum Vaccines Immunother. 2013 Nov 1;9(11):2304–10.

17.	Nolan T, Santolaya ME, de Looze F, Marshall H, Richmond P, Henein S, et al. Antibody persistence and booster response in adolescents and young adults 4 and 7.5 years after immunization with 4CMenB vaccine. Vaccine. 2019 Feb 21;37(9):1209–18.

18.	Gan G, Janhavi A, Tong G, Lim JT, Dickens BL. The need for pre-emptive control strategies for mpox in Asia and Oceania. Infect Dis Model. 2024 Mar 1;9(1):214–23.

19.	Johnson HC, Lafferty EI, Eggo RM, Louie K, Soldan K, Waller J, et al. Effect of HPV vaccination and cervical cancer screening in England by ethnicity: a modelling study. Lancet Public Health. 2018 Jan 1;3(1):e44–51.
