# Supplementary material for: Potential public health impacts of gonorrhea vaccination programmes under declining incidences: A modeling study
Source: PLoS Med. 2025 Feb 7;22(2):e1004521. doi: 10.1371/journal.pmed.1004521 (PMC11805383; doi:10.1371/journal.pmed.1004521)
Supplement: Attachment — Submitted filename: Response_Document_28122024.docx [file pmed.1004521.s005.docx]

**Editor Comments**

Dear Dr. Geng,

Thank you very much for re-submitting your manuscript "Potential public health impacts of gonorrhoea vaccination programmes under declining incidences: a modelling analysis" (PMEDICINE-D-24-02533R2) for review by PLOS Medicine. I am writing on behalf of my colleague Dr. Sunny.

I have discussed the paper with my colleagues and the academic editor and it was also seen again by 2 of the original reviewers. I am pleased to say that provided the remaining editorial and production issues are dealt with we are planning to accept the paper for publication in the journal.

The remaining issues that need to be addressed are listed at the end of this email. Any accompanying reviewer attachments can be seen via the link below. Please take these into account before resubmitting your manuscript:

***Please note while forming your response, if your article is accepted, you may have the opportunity to make the peer review history publicly available. The record will include editor decision letters (with reviews) and your responses to reviewer comments. If eligible, we will contact you to opt in or out.***

In revising the manuscript for further consideration here, please ensure you address the specific points made by each reviewer and the editors. In your rebuttal letter you should indicate your response to the reviewers' and editors' comments and the changes you have made in the manuscript. Please submit a clean version of the paper as the main article file. A version with changes marked must also be uploaded as a marked up manuscript file.

Please also check the guidelines for revised papers at <http://journals.plos.org/plosmedicine/s/revising-your-manuscript> for any that apply to your paper. If you haven't already, we ask that you provide a short, non-technical Author Summary of your research to make findings accessible to a wide audience that includes both scientists and non-scientists. The Author Summary should immediately follow the Abstract in your revised manuscript. This text is subject to editorial change and should be distinct from the scientific abstract.

We expect to receive your revised manuscript within 1 week. Please email us ([plosmedicine@plos.org](mailto:plosmedicine@plos.org)) if you have any questions or concerns.

We ask every co-author listed on the manuscript to fill in a contributing author statement. If any of the co-authors have not filled in the statement, we will remind them to do so when the paper is revised. If all statements are not completed in a timely fashion this could hold up the re-review process. Should there be a problem getting one of your co-authors to fill in a statement we will be in contact. YOU MUST NOT ADD OR REMOVE AUTHORS UNLESS YOU HAVE ALERTED THE EDITOR HANDLING THE MANUSCRIPT TO THE CHANGE AND THEY SPECIFICALLY HAVE AGREED TO IT.

Please ensure that the paper adheres to the PLOS Data Availability Policy (see <http://journals.plos.org/plosmedicine/s/data-availability>), which requires that all data underlying the study's findings be provided in a repository or as Supporting Information. For data residing with a third party, authors are required to provide instructions with contact information for obtaining the data. PLOS journals do not allow statements supported by "data not shown" or "unpublished results." For such statements, authors must provide supporting data or cite public sources that include it.

To enhance the reproducibility of your results, we recommend that you deposit your laboratory protocols in [protocols.io](http://protocols.io/), where a protocol can be assigned its own identifier (DOI) such that it can be cited independently in the future. Additionally, PLOS ONE offers an option to publish peer-reviewed clinical study protocols. Read more information on sharing protocols at <https://plos.org/protocols?utm_medium=editorial-email&utm_source=authorletters&utm_campaign=protocols>

Please review your reference list to ensure that it is complete and correct. If you have cited papers that have been retracted, please include the rationale for doing so in the manuscript text, or remove these references and replace them with relevant current references. Any changes to the reference list should be mentioned in the rebuttal letter that accompanies your revised manuscript.

Please note, when your manuscript is accepted, an uncorrected proof of your manuscript will be published online ahead of the final version, unless you've already opted out via the online submission form. If, for any reason, you do not want an earlier version of your manuscript published online or are unsure if you have already indicated as such, please let the journal staff know immediately at [plosmedicine@plos.org](mailto:plosmedicine@plos.org).

If you have any questions in the meantime, please contact me or the journal staff on [plosmedicine@plos.org](mailto:plosmedicine@plos.org).  

We look forward to receiving the revised manuscript by Dec 30 2024 11:59PM.   

Sincerely,

Alison Farrell, PhD
Senior Editor 
PLOS Medicine
[plosmedicine.org](http://plosmedicine.org/)

------------------------------------------------------------
**Requests from Editors:**

**Comment:** We ask that you address the remaining comments from Reviewer 3 in the main text and discussion, expand the limitations discussion to address their concerns, and clarify where limited data availability may have impacted GC incidence estimates.

**Response:** We thank editors for reviewing our revised manuscript and the editing comments made for this round. Below, we responded point by point to each comment by editors and reviewers. We addressed the justifications in the discussion section to clarify the data limitation that may impact gonorrhoea modelling estimates.

**Comments:** Please also address the following text edits:

**Comment:** line 16: designed to protect against Neisseria meningitidis infection

**Response:** We thank the editor for the comments and have amended the statement:

***“****Evidence suggests that the four-component serogroup B meningococcal (4CMenB) vaccine designed to protect against Neisseria meningitidis”*

**Comment:** line 23: should this be 'acquisition and transmission' or only 'transmission'?

**Response:** We thank the editor for the comments and have amended the statement:

*“understand the potential public health impact of 4CMenB in reducing gonorrhea acquisition and transmission in men”*

**Comment:** lines 26,27: offering vaccination to individuals attending sexual health clinics and who were diagnosed with gonorrhoea

**Response:** We thank the editor for the comments and have amended the statement:

***“****offering vaccination to individuals attending sexual health clinics and who were diagnosed with gonorrhoea****”***

**Comment:** line 31: delete 'during'

**Response:** We thank the editor for the comments and have amended the statement

**Comment:** line 37: what is the intervention time?

**Response:** We thank the editor for the comments and have amended the statement to be clear that it was the time over the vaccination programme was implemented:

*“For a hypothetical 10-year vaccination programme, VbE had 14.18% of MSM gonorrhea cases averted over the time the programme was implemented”*

**Comment:** line 39: please correct phrasing ' but three times efficient higher'

**Response:** We thank the editor for the comments and have amended the statement:

*“VoD had a smaller impact (12.04% averted cases (95% CrI 7.12%-15.00%)) but was three times more efficient than VoA in terms of averted cases per dose.”*

**Comment:** line 45: replace which with that

**Response:** We thank the editor for the comments and have amended the statement:

*“Sensitivity analyses that varied vaccine duration of protection and efficacy indicated that VaR had the greatest public health impact”*

**Comment:** line 51: can be considered for what? Please qualify and more clearly articulate the study implications.

**Response:** We thank the editor for the comments and have amended the statement to more clearly articulate the study implications

*“****Conclusions*** *Vaccination of MSM against gonorrhea according to risk in sexual health clinics in Singapore can be considered to reduce gonorrhea acquisition and transmission. Development of gonorrhea-specific vaccines which focuses on protective efficacy and the implementation of efficient vaccination programmes can maximize public health impact.”*

**Comment:** lines 51, 52: The abstract does not focus on duration of protection, so why does the concluding sentence? Please revise sentence, and/or provide a sentence in the results section of the abstract pertaining to this issue. I suggest focusing the last sentence on the development of gonorrhoea-specific vaccines and the design and implementation of vaccination programmes to maximise their protective efficacy and public health impact.

**Response:** We thank the editor for the comments. We have now amended the statement to focus on optimal vaccination programmes, and a vaccine profile which optimizes protective efficacy, so that it segues better from the methods and findings section of the abstract:

*“****Conclusions*** *Vaccination of MSM against gonorrhea according to risk in sexual health clinics in Singapore can be considered to reduce gonorrhea acquisition and transmission. Development of gonorrhea-specific vaccines which focuses on protective efficacy and the implementation of efficient vaccination programmes can maximize public health impact.”*

**Comment:** line 57: Vaccination of

**Response:** We thank the editor for the comments and have amended the statement:

*“Vaccination of high risk groups”*

**Comment:** line 61: use of "can confer" seems too strong given that there are no approved gonorhhea-specific vaccines. Please consider revising phrasing. E.g. n. Gonorrhoea-specific vaccines may confer greater public health impacts by maximising efficacy over the duration of protection. Separately, vaccine developers typically prioritize efficacy, therefore is this qualification (relative to duration) necessary, versus focusing on the implementation of targeted vaccination programmes, which the study models?

**Response:** We thank the editor for the comments and we have amended the statement, such that it more specifically shows that under the targeted vaccination programmes which are modelled, prioritizing efficacy vs. duration of protective brings forth greater public health impacts:

*“Gonorrhoea-specific vaccines may confer greater public health impacts by maximising efficacy over the duration of protection under modelled vaccination programmes.”*

**Comment:** lines 95-97: please include in this sentence the pathogen targeted by MenB.

**Response:** We thank the editor for the comments and have amended the statement:

*“The interest in vaccine prevention against N. gonorrhoeae arose from observations of declining gonorrhea incidence with the use of the MenB (one type of meningococcal serogroups) outer membrane vesicles (OMV) vaccine in Norway [1] and Cuba [2,3], which were used to protect against meningococcal disease caused by meningococcal group B bacteria”*

**Comment:** line 102: here and throughout, please use lower case g for gonorrhea

**Response:** We thank the editor for the comments and have amended to use lower case g for gonorrhea throughout the text.

**Comment:** lines 118, 119: 'potential' used 2x. Please remove one use.

**Response:** We thank the editor for the comments and have amended the statement:

*“In this study, we aimed to guide potential public health decisions regarding the implementation of ~~potential~~ different gonorrhea vaccination programmes.”*

**Comment:** line 123: add 'among MSM'? Also mention the time range(s) of the analyses and modelling?

**Response:** We thank the editor for the comments and have amended the statement:

*“This was calibrated using 3 different case time series of gonorrhoea incidence among MSM from 2004 to 2018. We examined how the impact of vaccination programs over 2024 to 2034 depends on vaccine-specific factors, such as uptake, efficacy, and duration of protection, as well as different epidemic trajectories.”*

**Comment:** replace monkeypox with mpox

**Response:** We thank the editor for the comments and have amended the statement:

*“Pre-emptive vaccination is expected to reduce the likelihood of mpox outbreaks in the same study setting, especially in targeting of dense and well-connected sexual contact networks”*

**Comment:** Please start paragraph discussing study limitations with a sentence to that effect.

**Response:** We thank the editor for the comment. We have now started the paragraph discussing study limitations with a summary statement:

*"There are certain limitations to this research study. In our exploration of....""*

**Comment:** Please directly state in the text that there aren’t publicly available gonorrhea incidence estimates for Singapore beyond 2020 (as per your reply to reviewer 3)

**Response:** We thank the editor for the comments and have amended the statement:

*“****Data*** *The model was calibrated using a Bayesian inference framework using proxies of annual gonorrhoea MSM incidence from 2004 to 2018 in Singapore. MSMs were defined to be men who report ever having sex with a man. As actual breakdowns of annual gonorrhoea MSM incidence were not available in the study setting, we assumed that this was proxied by (****1****) male incidence less the female incidence of gonorrhoea (****2****) the male incidence of gonorrhoea, i.e the upper bound to the annual gonorrhoea MSM incidence (****3****) the male incidence multiplied by the proportion of reported gonorrhoea cases in the MSM population in other study settings (See supplementary information, Table S1). Raw annual gonorrhoea incidence was obtained from the communicable disease surveillance system of Singapore from 2004-2020 [4] and excluded observations in 2019, 2020 due to COVID-19. Publicly available estimates for gonorrhea incidence are not available beyond 2020. We calibrated our model under each scenario and reported model outputs based on the first scenario as our main results.”*

**Comments:**
We also ask you to make the following formatting edits to the manuscript:
* It appears that one or more study authors is affiliated with one or more of the agencies that funded the study. Thus, the statement “The funders had no role in study design, data collection and analysis, decision to publish, or preparation of the manuscript” does not apply. Please revise the Financial Disclosure accordingly, as in "[Author name] is [author's role] at [funding agency]. The funders had no other role in study design…..”

**Response:** We have revised the Financial Disclosure accordingly in the submission portal.

**Comment:** Please expand upon your data availability statement.
"* The Data Availability Statement (DAS) requires revision. For each data source used in your study:

a) If the data are freely or publicly available, note this and state the location of the data: within the paper, in Supporting Information files, or in a public repository (include the DOI or accession number).

b) If the data are owned by a third party but freely available upon request, please note this and state the owner of the data set and contact information for data requests (web or email address). Note that a study author cannot be the contact person for the data.

c) If the data are not freely available, please describe briefly the ethical, legal, or contractual restriction that prevents you from sharing it. Please also include an appropriate contact (web or email address) for inquiries (again, this cannot be a study author).

**Response:** All the data source used in our study are applied to a) freely or publicly available and we have stated the location of the data within the paper, and edited in the data availability statement:

*“All code and data used for this research can be accessed at* [*https://github.com/lineliott/gonovaxSG/*](https://github.com/lineliott/gonovaxSG/)*.”*

**Comment:** * Please review your manuscript and edit to ensure compliance with our inclusive language requirements <https://journals.plos.org/plosmedicine/s/human-subjects-research#loc-categorization>

**Response:** We have ensured that our manuscript complies with inclusive language requirements.

**Comment:** Please define all elements of box plots in the figure caption - center line, box limits and whiskers.

**Response:** We have defined all elements of box plots in the figure caption (i.e in Figures 1 to 4).

**Comment:** Please confirm that your title complies with to PLOS Medicine's style. Your title must be nondeclarative and not a question. It should begin with main concept if possible. "Effect of" should be used only if causality can be inferred, i.e., for an RCT. Please place the study design ("A randomized controlled trial," "A retrospective study," "A modelling study," etc.) in the subtitle (ie, after a colon).

**Response:** We have ensured that the title compiles with PLOS Medicine’s style.

*“Potential public health impacts of gonorrhoea vaccination programmes under declining incidences: a modelling study”*

**Comment:** Your abstract exceed word limit of 500 – Editorial Manager system warning.

**Response:** We have amended the abstract section accordingly.

**Background** Gonorrhea is the second most common sexually transmitted infections notified in Singapore in 2023~~, despite historically decreasing incidence.~~ Evidence suggests that the ~~four-component serogroup B meningococcal (~~4CMenB~~)~~ vaccine designed to protect against *Neisseria meningitidis* infection may offer partial cross-protection against gonorrhea. This ~~has~~ generated interest in using 4CMenB for the purpose of staving gonorrhea transmission. ~~Here,~~ We explored the efficacy of potential gonorrhea vaccination strategies in the context of historically declining gonorrhea incidence.

**Methods and Findings** We employed an integrated transmission-dynamic model, calibrated using Bayesian methods to local surveillance data to understand the potential public health impact of 4CMenB in reducing gonorrhea acquisition and transmission in men who have sex with men (MSM) in Singapore. We explored the efficacy of implementing 6 vaccination programmes, (**1**) offering vaccination to all male adolescents in schools (vaccination before entry[VbE]), (**2**) offering vaccination to individuals attending sexual health clinics for testing (vaccination on attendance[VoA]); (**3**) offering vaccination to individuals attending sexual health clinics and who were diagnosed with gonorrhea (vaccination on diagnosis[VoD]); or (**4**) vaccination according to risk(VaR), by offering vaccination to patients who were diagnosed with gonorrhea plus individuals who tested negative but report having more than five sexual partners per year. We further examined how altering (**5**) VoA and (**6**) VoD strategies changed if the strategies only targeted high risk groups (VoA(H),VoD(H)). We assessed efficacy by examining vaccination impact relative to ~~the baseline case of~~ no vaccination and when behavioural parameters ~~(sexual risk behaviour and screening rates)~~ were held constant. We further ascertained the effects of varying vaccine uptake (10%,33%,100%), vaccine efficacy (22%,31%,47%) and duration of protection (1.5,4,7.5 years) on the effectiveness of each vaccination strategy.

For a hypothetical 10-year vaccination programme, VbE had 14.18% of MSM gonorrhea cases averted over the time the programme was implemented. VoA had the highest protective impact on the MSM population with 40.26% averted cases (95%CrI:18.32%-52.57%) but required more vaccine doses than any other strategy~~, whereas~~ VoD had a smaller impact (12.04% averted cases (95%CrI:7.12%-15.00%)) but was three times more efficient than VoA in terms of averted cases per dose. VoA(H) and VoD(H) improved the efficiency of VoA and VoD strategies by increasing averted cases per dose to 0.22 and 0.24 respectively, but conferred similar protective effects as VoA (VoA(H): 40.10% averted cases (95%CrI:18.14%-52.55%)) and VoD (VoD(H): 12.04% averted cases (95%CrI:7.12%-15.00%)) respectively. VaR (40.10% averted cases (95%CrI:18.14%-52.55%)) had almost the same impact as VoA but was more efficient by requiring administration of fewer doses than VoA, with 0.21 (95%CrI:0.12-0.27) averted cases per dose ~~at 0.21 (95% CrI: 0.12-0.27)~~. Sensitivity analyses ~~that varied vaccine duration of protection and efficacy~~ indicated that VaR had the greatest public health impact with the highest number of averted cases per dose for vaccines of any efficacy or duration of protection (or both), although VoD and VoD(H) saved more vaccine resource and had the highest number averted MSM cases per dose for highly protective vaccines of long protection.

**Conclusions** Vaccination of MSM against gonorrhea according to risk in sexual health clinics in Singapore can be considered to reduce gonorrhea acquisition and transmission. Development of gonorrhea-specific vaccines which focuses on protective efficacy and the implementation of efficient vaccination programmes can maximize public health impact.

**Comments from Reviewers:**

**Reviewer #1:**

**Comment:** Thank you to the editors and reviewers, as well as the author for addressing all the comments. I agree that this manuscript has been significantly improved and is now suitable for publication.
— Xuecheng Yin

**Response:** We thank the reviewer for the kind comments

**Reviewer #3:**

**Comment:** I appreciate the authors' comprehensive revisions and the effort to address the concerns raised during the initial review. The manuscript is much improved, with additional sensitivity analyses, clarified justifications, and expanded discussions on key modelling assumptions. The responses were well-detailed and thoughtful. Below are some additional comments that, if addressed, could further strengthen the manuscript.

**Response:** We thank the reviewer for the valuable comments. Below, we address your comments point by point.

**Comment:** While the authors provided a rationale for choosing a linear functional form, it would add value to briefly discuss the implications of alternative forms in the results or limitations section. For example, if exponential decay was tested but found less fitting, highlighting why it was inappropriate would strengthen the justification.

**Response:** We thank the reviewer for suggesting on clarifying the transmission probability formation. We now have added detailed explanation regarding the choice of linear function to justify our study setting in the discussion section:

*“We also characterised the change in transmission probability over the observation period using a linear function, or assumed that they were constant in the future. Our exploration included an alternative exponentially decaying functional form to characterize changes in transmission probabilities, but this did not fit to observed historical trends appropriately.*

**Comment:** The model does not currently account for feedback loops, such as changes in sexual risk behaviour due to perceived protection from vaccination. A discussion on how these unmodeled dynamics might impact real-world outcomes would be valuable.

**Response:** We thank the reviewer for highlighting the matter of potential real-world impact induced by unmodeled dynamics. The change in sexual behaviour risk group over years, which caused by perceiving protection from vaccination, is currently not captured in the model. The incorporation of these data into our transmission model could significantly enhance its predictive capabilities. Unfortunately, we lacked these data in our study setting and instead relied on regional survey data from other sources. We have addressed this limitation, and also provided a discussion on how these unmodeled dynamics can impact real-world outcomes:

*“There was also a lack of community sexual behaviour data, such as information on sexual networks, to characterize behaviors and proportions of key at-risk populations in the study setting. Hence, it was difficult to parameterize changes in sexual behaviours after vaccination over study period. We relied on survey data from a UK MSM demographic survey to parameterize screening rates in high/low sexual activity groups in Singapore, and explored the impact of different screening and sexual behaviour parameters in sensitivity analysis. However, due to recent legislative changes in decriminalizing sex between consenting adult males in the study setting, the practicality of collecting such data in the study setting is increased, and future work should seek to ascertain potential vaccination programme effectiveness using more accurate local data, as well as understand the impact of vaccination on sexual behaviours. We hypothesize that vaccination may be accompanied by a shift towards healthier sexual behavioral practices among the study population, and thereby increase the public health impact of vaccination programmes.”*

**Comment:** Including a discussion on how different public health strategies (e.g., intensified outreach, rapid diagnostics) could interact with vaccination to enhance impact might make the findings more actionable.

**Response:** We thank the reviewer for pointing out to consider about public health strategies that our model has not included. We have now suggested how other public health strategies can interact with the modelled vaccination programmes in the discussion section:

*“Combining modelled vaccination programmes with alternative public health strategies may also amplify the public health impact of the vaccination programmes. These scenarios can be explored in future studies. Utilisation of rapid test kits and intensified outreach can enhance the uptake rate of vaccination programmes in at-risk populations. Mitigation of potential stigma towards gonorrhea through intensified outreach, and increasing diagnosis rates in asymptomatics by roll-out of rapid test can facilitate timely vaccination and testing, and potentially enhance the impact of gonnorhea vaccination programmes.”*

**Comment:** Although the revised legends are improved, further clarity on metrics (e.g., how averted cases per dose are calculated) in the main text would enhance the utility of figures for the reader.

**Response:** We thank the reviewers for the suggestion on improving readability of figure 2. We elaborated the definitions of metrics in the method section and incorporated additional explanation in the legend of figure 2.

*“To quantify the raw public health impact of each vaccination strategy, we reported the*

(1) *Averted number of cases– computed as the difference in the number of treated gonorrhea patients between the baseline case and the respective vaccination strategy.*

*we reported programme efficiency by computing the*

(2) *percentage of averted cases – computed as the percentage difference in the number of treated gonorrhea patients between the baseline case and the respective vaccination strategy.*
(3) *averted cases per dose – computed as the difference in the number of treated gonorrhea patients between the baseline case and the respective vaccination strategy, divided by number of vaccination doses given.*

*We also computed vaccination-strategy specific metrics to understand the resource needs and uptake profile for each strategy:*

(4) *number of vaccinations administered – measured by the number of times vaccination was provided. We assumed that all individuals who accepted vaccination took two doses for primary protection and 1 booster dose for revaccination.*
(5) *primary uptake ratio – which tracked the proportion of individuals who received vaccine protection for the first time given the number of vaccinations.”*

*“Metrics are averted cases by percentage (AP: computed as the percentage difference between number of treated gonorrhoea patients between the baseline case and the respective vaccination strategy), averted cases per dose (AD: computed as the difference between number of treated gonorrhoea patients between the baseline case and the respective vaccination strategy, divided by number of vaccination doses given), number of vaccinations administered (NV: measured by the number of times vaccination was provided. We assumed that all individuals who accepted vaccination took a regiment of doses for primary protection and 1 booster dose for revaccination), and primary uptake ratio (PU: tracked the proportion of individuals who received vaccine protection for the first time given the number of vaccination).”*

**Comment:** While sensitivity analyses were conducted, a summary or visualization of how varying assortativity impacts key outcomes (e.g., averted cases, vaccination efficiency) could make the implications more accessible.

**Response:** We thank the reviewer for the comment. We have now added additional tabular summaries of how varying assortativity impacts key outcomes across varied assortativity values in the supplementary information section:

| **Strategy** | **-50%** | **-25%** | **Main analysis** | **+25%** | **+50%** |
| --- | --- | --- | --- | --- | --- |
| VbE | 618  (0-1813) | 948  (11-1900) | 1648 (942-2120) | 1906  (1067-2530) | 1975  (1084-2661) |
| VoD(H) | 340  (0-1448) | 549  (0-1635) | 1427 (551-2176) | 2426  (713-4395) | 3053  (783-5572) |
| VoD | 340  (0-1449) | 549  (0-1636) | 1427 (551-2176) | 2426  (714-4395) | 3054  (784-5572) |
| VoA | 1549  (0-5335) | 2409  (23-5596) | 4704 (1948-6947) | 6265  (2461-9736) | 7021  (2461-11414) |
| VoA(H) | 1539  (0-5292) | 2395  (23-5561) | 4685 (1938-6922) | 6246  (2461-9716) | 7004  (2461-11395) |
| VaR | 1539  (0-5292) | 2396  (23-5562) | 4685 (1939-6922) | 6246  (2461-9716) | 7004  (2461-11396) |

**Table S21 Summary of averted cases (total)** **for various vaccination programmes from 2024 to 2034, by varying the assortativity parameter (**$\boldsymbol{\epsilon}$**).** We report posterior means and accompanying 95% credible intervals. Estimates presented are relative to the baseline scenario of no intervention, with transmission probabilities from 2024 to 2034 kept stable at 2018 levels.

| **Strategy** | **-50%** | **-25%** | **Main analysis** | **+25%** | **+50%** |
| --- | --- | --- | --- | --- | --- |
| VbE | 16.00  (6.32-21.78) | 17.50  (10.75-21.81) | 14.18  (9.11-17.08) | 11.37  (7.04-15.70) | 9.99  (5.96-15.25) |
| VoD(H) | 4.63  (0.00-12.35) | 7.17  (0.17-12.97) | 12.04  (7.12-15.00) | 13.71  (7.97-17.66) | 14.25  (8.34-18.48) |
| VoD | 4.63  (0.00-12.36) | 7.18  (0.17-12.98) | 12.04  (7.12-15.00) | 13.71  (7.97-17.66) | 14.25  (8.34-18.48) |
| VoA | 37.53  (11.56-54.56) | 42.15  (18.31-54.51) | 40.26  (18.32-52.57) | 36.58  (17.83-49.13) | 34.33  (16.97-47.79) |
| VoA(H) | 37.29  (11.49-54.36) | 41.95  (18.21-54.38) | 40.10  (18.14-52.55) | 36.46  (17.67-48.87) | 34.23  (16.97-47.68) |
| VaR | 37.30  (11.49-54.36) | 41.95  (18.22-54.38) | 40.10  (18.14-52.55) | 36.46  (17.68-48.87) | 34.23  (16.97-47.68) |

**Table S22 Summary of averted cases by percentage** **for various vaccination programmes from 2024 to 2034, by varying the assortativity parameter (**$\boldsymbol{\epsilon}$**).** We report posterior means and accompanying 95% credible intervals. Estimates presented are relative to the baseline scenario of no intervention, with transmission probabilities from 2024 to 2034 kept stable at 2018 levels.

| **Strategy** | **-50%** | **-25%** | **Main analysis** | **+25%** | **+50%** |
| --- | --- | --- | --- | --- | --- |
| VbE | 0.002  (0.000-0.005) | 0.003  (0.000-0.006) | 0.005  (0.003-0.006) | 0.006  (0.003-0.007) | 0.006  (0.003-0.008) |
| VoD(H) | 0.09  (0.00-0.27) | 0.14  (0.00-0.28) | 0.24  (0.14-0.31) | 0.28  (0.15-0.37) | 0.29  (0.16-0.39) |
| VoD | 0.08  (0.00-0.22) | 0.12  (0.00-0.23) | 0.22  (0.12-0.28) | 0.26  (0.14-0.35) | 0.27  (0.14-0.38) |
| VoA | 0.02  (0.00-0.07) | 0.03  (0.00-0.08) | 0.06  (0.02-0.10) | 0.07  (0.03-0.13) | 0.08  (0.03-0.15) |
| VoA(H) | 0.08  (0.00-0.24) | 0.12  (0.00-0.25) | 0.22  (0.12-0.28) | 0.27  (0.15-0.36) | 0.29  (0.15-0.40) |
| VaR | 0.07  (0.00-0.23) | 0.11  (0.00-0.24) | 0.21 (0.12-0.27) | 0.26  (0.14-0.36) | 0.28  (0.15-0.39) |

**Table S23 Summary of averted cases per dose** **for various vaccination programmes from 2024 to 2034, by varying the assortativity parameter (**$\boldsymbol{\epsilon}$**).** We report posterior means and accompanying 95% credible intervals. Estimates presented are relative to the baseline scenario of no intervention, with transmission probabilities from 2024 to 2034 kept stable at 2018 levels.

**Comment:** Ensure the most recent studies on vaccine efficacy (e.g., Abara et al., 2024) are integrated seamlessly into the discussion, particularly in comparison with older studies.

**Response:** We thank the reviewer for the comment. We now have included justification regarding the impact of updating vaccine efficacy on this modelling study in the discussion section.

*“Our analysis may also require constant updates in conjunction with rapidly developing literature surrounding the protective efficacy of 4CMenB against gonorrhea infection. The most recent systematic review reported an pooled efficacy of 32.4% [5], a finding that supported the parameters used in our primary analysis. However, a more recent retrospective cohort study reported a 23% protective effectiveness against gonorrhea [6], highlighting heterogeneities in published protective efficacy estimates.”*

**Reference:**

1.	Whelan J, Kløvstad H, Haugen IL, Holle MRDR van B, Storsaeter J. Ecologic Study of Meningococcal B Vaccine and Neisseria gonorrhoeae Infection, Norway. Emerg Infect Dis. 2016 Jun;22(6):1137–9.

2.	Pérez O, del Campo J, Cuello M, González E, Nuñez N, Osmir C, et al. Mucosal approaches in Neisseria Vaccinology. VacciMonitor. 2009 Jan 1;

3.	Reyes Díaz LM, Lastre González M de SJB, Cuello M, Sierra-González VG, Ramos Pupo R, Lantero MI, et al. VA-MENGOC-BC Vaccination Induces Serum and Mucosal Anti Neisseria gonorrhoeae Immune Responses and Reduces the Incidence of Gonorrhea. Pediatr Infect Dis J. 2021 Apr;40(4):375.

4.	MOH | Communicable Diseases Surveillance in Singapore 2019-2020 [Internet]. [cited 2024 May 15]. Available from: https://www.moh.gov.sg/resources-statistics/reports/communicable-diseases-surveillance-in-singapore-2019-2020

5.	Abara WE, Kirkcaldy RD, Bernstein KT, Galloway E, Learner ER. Effectiveness of MenB-4C Vaccine Against Gonorrhea: A Systematic Review and Meta-analysis. J Infect Dis. 2024 Jul 31;jiae383.

6.	Abara WE, Modaressi S, Fireman B, Klein NP, Layefsky E, Goddard K, et al. Effectiveness of a serogroup B meningococcal vaccine against gonorrhea: A retrospective study. Vaccine. 2024 Dec 2;42(26):126312.
